# Extrasynaptic NMDA receptors in acute and chronic excitotoxicity: implications for preventive treatments of ischemic stroke and late-onset Alzheimer’s disease

**DOI:** 10.1186/s13024-023-00636-1

**Published:** 2023-07-03

**Authors:** Shan P. Yu, Michael Q. Jiang, Seong S. Shim, Soheila Pourkhodadad, Ling Wei

**Affiliations:** 1 Department of Anesthesiology, Emory University School of Medicine, Atlanta, GA 30322 USA; 2 Center for Visual & Neurocognitive Rehabilitation, Atlanta VA Medical Center, Decatur, GA 30033 USA

**Keywords:** Glutamate, NMDA receptors, Excitotoxicity, GluN2B subunit, GluN3A subunit, Extrasynaptic NMDARs, Ca^2+^ homeostasis, Ischemic stroke, Alzheimer’s disease, Memantine

## Abstract

Stroke and late-onset Alzheimer’s disease (AD) are risk factors for each other; the comorbidity of these brain disorders in aging individuals represents a significant challenge in basic research and clinical practice. The similarities and differences between stroke and AD in terms of pathogenesis and pathophysiology, however, have rarely been comparably reviewed. Here, we discuss the research background and recent progresses that are important and informative for the comorbidity of stroke and late-onset AD and related dementia (ADRD). Glutamatergic NMDA receptor (NMDAR) activity and NMDAR-mediated Ca^2+^ influx are essential for neuronal function and cell survival. An ischemic insult, however, can cause rapid increases in glutamate concentration and excessive activation of NMDARs, leading to swift Ca^2+^ overload in neuronal cells and acute excitotoxicity within hours and days. On the other hand, mild upregulation of NMDAR activity, commonly seen in AD animal models and patients, is not immediately cytotoxic. Sustained NMDAR hyperactivity and Ca^2+^ dysregulation lasting from months to years, nevertheless, can be pathogenic for slowly evolving events, i.e. degenerative excitotoxicity, in the development of AD/ADRD. Specifically, Ca^2+^ influx mediated by extrasynaptic NMDARs (eNMDARs) and a downstream pathway mediated by transient receptor potential cation channel subfamily M member (TRPM) are primarily responsible for excitotoxicity. On the other hand, the NMDAR subunit GluN3A plays a “gatekeeper” role in NMDAR activity and a neuroprotective role against both acute and chronic excitotoxicity. Thus, ischemic stroke and AD share an NMDAR- and Ca^2+^-mediated pathogenic mechanism that provides a common receptor target for preventive and possibly disease-modifying therapies. Memantine (MEM) preferentially blocks eNMDARs and was approved by the Federal Drug Administration (FDA) for symptomatic treatment of moderate-to-severe AD with variable efficacy. According to the pathogenic role of eNMDARs, it is conceivable that MEM and other eNMDAR antagonists should be administered much earlier, preferably during the presymptomatic phases of AD/ADRD. This anti-AD treatment could simultaneously serve as a preconditioning strategy against stroke that attacks ≥ 50% of AD patients. Future research on the regulation of NMDARs, enduring control of eNMDARs, Ca^2+^ homeostasis, and downstream events will provide a promising opportunity to understand and treat the comorbidity of AD/ADRD and stroke.

**Supplementary Information:**

The online version contains supplementary material available at 10.1186/s13024-023-00636-1.

## Background

Acute ischemic stroke is a leading cause of death and disability in aging populations [1, 2]. One-third of stroke patients develop post-stroke dementia within 3 years and show pathological alterations resembling those of Alzheimer’s disease (AD), including the development of delayed cell death, cognitive decline, and β-amyloid (Aβ) deposition in the brain [3, 4]. As progressive neurodegenerative diseases, late-onset AD and AD-related dementia (ADRD) account for over 60–80% of dementia cases in people over 65 [5, 6]. These patients have a high risk of stroke; over half of them experience at least one stroke attack and suffer more severe outcomes, including increased mortality rates than non-AD patients [7]. A national longitudinal cohort study (2007–2017) compared 12,629 ischemic stroke patients with dementia to 57,954 matched stroke non-dementia controls. The study identified dementia before stroke as an independent predictor of death. Over time, mortality in patients with dementia remained increased [8]. Even though stroke and late-onset AD/ADRD are interrelated common comorbidities in the same aging/aged individuals [7, 9, 10], the pathophysiology associated with different time courses of disease development and their destructive impacts on each other are not explicitly understood. Historically and currently, stroke and AD have been investigated in separate research fields and are regarded as distinct acute and chronic brain disorders, respectively. Increasing evidence, however, has revealed that stroke and AD share many hallmark pathophysiological alterations, including overactivations of glutamatergic N-methyl-D-aspartate (NMDA) receptors (NMDARs), increases in intracellular free Ca^2+^ ([Ca^2+^]_i_), disruptions of energy metabolism, excitotoxicity-induced neuronal loss, programmed cell death, synaptic/neural network impairments, neurovascular damage, neuroinflammation, Aβ/tau deposition, and progressive psychological/cognitive decline [2, 11–15]. Up to now, there is no effective disease-modifying treatment for either stroke or AD patients. After decades of study in both research fields, many neuroprotective and anti-amyloid treatments have failed clinical translations, showing inconsistent or no functional benefits because of a variety of dilemmas and obstacles [16–18].

Currently, approved treatments for ischemic stroke are limited to recombinant tissue plasminogen activator (rTPA) and endovascular thrombectomy, both of which can be highly effective within narrow therapeutic windows (4.5 and 6 h after stroke onset, respectively). Unfortunately, only a small fraction of stroke patients qualify clinically for these treatments [19]. For AD patients, cholinesterase inhibitors and the NMDAR low-affinity, uncompetitive antagonist memantine (MEM) are among the few drugs approved by FDA as symptomatic treatments for moderate-to-severe AD, albeit with variable efficacy [20]. Aducanumab is a human monoclonal antibody directed against aggregated soluble and insoluble forms of Aβ. Two Phase III clinical trials (EMERGE and ENGAGE) in mild cognitive impairment (MCI) and mild AD patients ended with conflicting results: EMERGE of high dose aducanumab slowed cognitive decline, but ENGAGE observed no clinical benefits and the trial had to be terminated early [21, 22]. Noticeably, among patients treated with high-dose aducanumab, ∼35% of them experienced amyloid-related imaging abnormalities (ARIAs), such as ARIA-related cerebral edema (ARIA-E), and a further ∼20% had ARIA-related microhemorrhages (ARIA-H), among other side effects. Even higher rates (43–65%) of ARIAs were observed in ApoE ε4 carriers [23]. These results were consistent with other clinical studies using anti-Aβ therapies [21, 24, 25]. Moreover, clinical trials with aducanumab and similar compounds have been carried out via FDA “accelerated approval”, their clinical efficacy, adverse effects, and risks including mortality remain to be further validated in peer-reviewed reports and clinical practice. The repeated failures and disruptive side-effects of anti-Aβ treatments based on the mechanism of familial AD (FAD) call for alternative strategies with innovative and out of the box thinking on the pathogenesis of late-onset AD in order to develop clinically effective and safe treatments for most AD cases.

Compelling findings from clinical analyzes reveal that ischemic stroke and AD/ADRD are significant risk factors for each other [8, 26–28]. A better understanding of the relationship and interactions between these two neurological diseases of the central nervous system (CNS) should help the development of treatments that target the shared mechanisms and show efficacy for both disorders in susceptible individuals. As a lesson learned from previous successes and failures, such clinical therapies can only be developed through unbiased, fact-based, and disease-specific mechanism-driven approaches consistent with clinical observations but not by digging further with conventional hypotheses inconsistent with clinical cases. If successful, multidisciplinary research will provide a breakthrough opportunity for the treatment of two neurological disorders that affect millions of people in the US and around the globe.

### Challenges in stroke and AD research and therapy development

There have been several groundbreaking discoveries from decades of neuroscience research that have encouraged cautious optimism for therapy developments of neurological disorders such as stroke and AD. Significant advances may include the identification of glutamate excitotoxicity mediated primarily by overactivation of NMDARs and a downstream TRPM-mediated pathway, of distinct roles of the synaptic and extrasynaptic NMDARs (sNMDARs and eNMDARs, respectively) in synaptic plasticity and neuronal excitotoxicity, and of robust neuroprotection achievable by using NMDAR antagonists as well as the endogenous protective mechanisms elicitable by preconditioning strategies against brain injuries. Unfortunately, none of these advances in basic and preclinical research has been successfully translated into clinical therapies, with MEM as an exception of limited success for advanced AD patients.

The failure of NMDAR antagonists in stroke clinical trials may be attributed to noticeable side effects, narrow therapeutic windows, the lack of vascular protection to restore local blood supply, and the complexity of cellular and molecular injury mechanisms in the human brain [16, 29, 30]. Despite these hurdles in the development of therapies for stroke, continual research confirms NMDAR-mediated excitotoxicity as the primary cell death mechanism [31], and the development of novel and safe NMDAR antagonists remains a top priority in stroke research. Consistent evidence suggests that the undesirable side effects of many NMDAR antagonists are possibly due to the paradoxical actions at synaptic and extrasynaptic NMDARs [32]. Therefore, in addition to the desire of targeting downstream pathways, more selective eNMDAR antagonists have become preferred choices to minimize side effects while enhancing the neuroprotective efficacy.

In basic and clinical AD research, the amyloid hypothesis has been challenged by compelling observations of Aβ-independent pathogenesis, pathophysiology, and pathology in animal models and human patients [33–36]. Commonly used transgenic mouse models generated using FAD genes such as a forced expression of mutant amyloid precursor protein (APP) and/or Aβ cascade genes do not accurately mimic late-onset sporadic AD in multiple aspects, including the trigger, origin, and time course of Aβ production as well as the lack of neuronal loss and tau pathology in some widely used FAD mice [37–39]. In some models with overexpressed APP, widespread Aβ deposition occurs but shows no subsequent cognitive deficits [40]. In a human APP (hAPP) transgenic mouse model of young and old ages (2–24 months of age), there was no evidence of amyloid deposits or neurodegeneration, even though the synaptic disruption was evident [41]. More significantly, while the Aβ pathology has been the diagnostic standard of AD and changes in Aβ deposition/plaques or soluble Aβ have been shown during AD development, many healthy individuals may have significant Aβ plaques and tau tangles in the brain while no signs of cognitive deficits [34, 42–44]. Some studies identified that at least 20–30% of healthy aging individuals showed substantial amyloid deposits in the brain but never developed dementia in their lifetimes [45]. Furthermore, clinical trials of anti-Aβ therapies that can successfully remove amyloid plaques have resulted in few functional improvements [33, 34, 45, 46].

It is now recognized that AD pathophysiology begins many years prior to clinical diagnosis, with various degrees of severity and different time courses of progression [47]. It is recognized that the onset age of Aβ deposition in the human brain is approximately 50 years old [48]. During the aging process before and around this age, the root mechanism triggering abnormal Aβ production in late-onset AD has been unclear except for propositions of genetic influence [48] and vague concepts such as “cognitive reserve” [49]. Aside from extensively delineated mechanisms of amyloid metabolism, there has been little information on the initial trigger(s) and year/decade-long process of endogenous amyloid pathology. Collectively, these inconsistencies and the lack of an endogenous association of Aβ pathology in disease progression suggest that alternative or additional mechanisms may be responsible for neuronal damage and the development of sporadic AD/ADRD [33–36].

As a significant paradigm shift in the understanding of AD progressive pathophysiology, modulations of brain hyperexcitability and the balance of excitatory/inhibitory activities are an emerging research area based on the Ca^2+^ hypothesis in AD pathogenesis [50–54]. For example, the anticonvulsant drug levetiracetam, which modulates glutamate release and neuronal excitatory/inhibitory balance, has been explored as a disease-modifying therapy for AD and has advanced to clinical trials [51, 53, 55, 56]. More evidence suggests that chronic attenuation of neuronal hyperactivity leads to reduced APP/Aβ accumulation, implying that neuronal hyperactivity can be an upstream event in the development of amyloid pathology [57]. However, the causal mechanism of slowly evolved degenerative excitotoxicity and distinctions between acute and chronic forms of excitotoxicity have not been explicitly defined. A better understanding of the causal mechanisms of glutamatergic hyperactivity, especially in both Aβ-dependent and Aβ-independent manners, may shed new light on the root pathogenesis and aid in the development of early treatments for late-onset sporadic AD and ADRD.

### Ionotropic glutamatergic NMDA receptors and subunits

Glutamate is the primary excitatory neurotransmitter in the CNS, and ionotropic glutamate receptors are responsible for neuronal communications crossing excitatory synapses. There are three subfamilies of ionotropic glutamate receptors: α-amino-3-hydroxy-5-methyl-4-isoxazolepropionic acid (AMPA) receptors, kainate receptors, and NMDARs [58–60]. Of these subtypes, NMDARs play major roles in Ca^2+^ homeostasis, neuronal excitability, synaptic plasticity, and excitotoxicity in neurophysiology and neuropathophysiology [61, 62]. NMDARs comprise GluN1 (NR1) subunits, GluN2 (NR2) subunits (GluN2A-2D), and a pair of GluN3 (NR3) subunits (GluN3A and GluN3B) [63, 64].

**Fig. 1:**
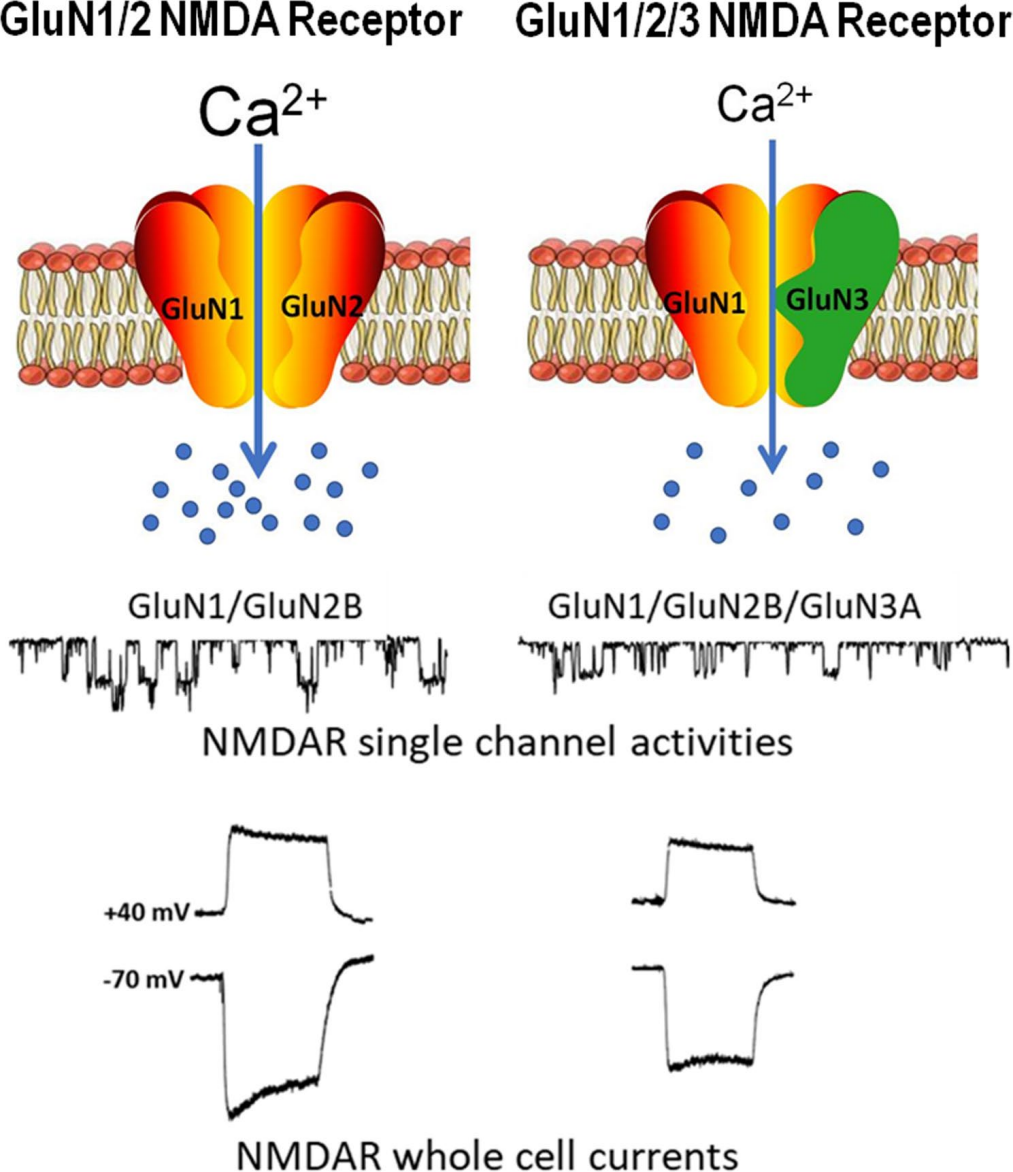
Composition of NMDA receptors and the regulatory role of the GluN3 subunit. Functional NMDA receptors are transmembrane heterotetramers embedded in the phospholipid bilayer of glutamatergic neurons, containing two GluN1 and two GluN2 subunits. The binding of the ligand leads to the opening of the receptor cation channel in an Mg^2+^- and voltage-depolarization manner. The NMDAR activity and its mediated Ca^2+^ influx have significant impacts on synaptic transmission, neuronal plasticity, psychological/cognitive functions, and cell fates. A GluN3 (GluN3A and 3B) subunit can replace one GluN2 in the triheteromeric complex, resulting in restrained single-channel opening activities and smaller whole-cell currents compared to GluN1/GluN2 receptors. The NMDA current traces are our unpublished data, which were recorded in an Mg2^+^-free extracellular solution

Although functional NMDARs can be formed by heterotetramers of two glycine/d-serine-binding GluN1 subunits paired with two glutamate-binding GluN2 subunits [65, 66] (Fig. 1), more recent research established that the majority of native NMDARs are triheteromers composed of two GluN1 and two unique GluN2 or a combination of GluN2 and GluN3 subunits [67, 68]. In contrast to diheteromeric structures, triheteromeric NMDARs display an intermediate sensitivity to glycine and glutamate due to either differences in channel ion conductance, open/close kinetics, or both as a result from the presence of various GluN2/3 subunits [67, 68]. Specifically, GluN3 subunits are differentiated by a positive charge in the pore-lining sequence that confers a unique structural alteration to receptors. NMDARs containing the GluN3 subunit are triheteromers composed of GluN1, GluN2, and GluN3 subunits. These receptors exhibit reduced Ca^2+^ permeability compared to GluN1/GluN2 receptors and attenuated Mg^2+^-block at hyperpolarized membrane potentials. Thus, the GluN3 subunit plays a unique role as a dominant-negative modulator in triheteromeric NMDARs [69–72]. In this event, GluN3 subunits may facilitate NMDA receptor activation resulting from their reduced magnesium sensitivity and reduce their conductance relative to GluN2A or GluN2B subunits. GluN1 and GluN3 can form diheteromeric receptors that are activated by glycine but not by glutamate [73]. Thus, strictly speaking, the GluN1/GluN3 complex is a glycine receptor, but essentially no longer a glutamate receptor.

NMDARs have been identified in non-neuronal cells, including astrocytes, oligodendrocytes, polydendrocytes (i.e. NG2 glial cells), and blood lymphocytes [74–76]. Since glial cells play important physiological and pathophysiological roles in the CNS, NMDAR subunits in these cells may exhibit special characteristics different from those of neurons. Our previous studies revealed GluN3A containing NMDARs even present in conducting cells of the kidney, which may regulate urinary concentrating capacity and play a protective role under ischemic/hypoxic conditions [77, 78]. The physiological and pathological roles of NMDARs in non-neuronal cells are not well understood and require further investigations.

### Functional roles of NMDARs and subunits at synaptic and extrasynaptic sites

**Fig. 2:**
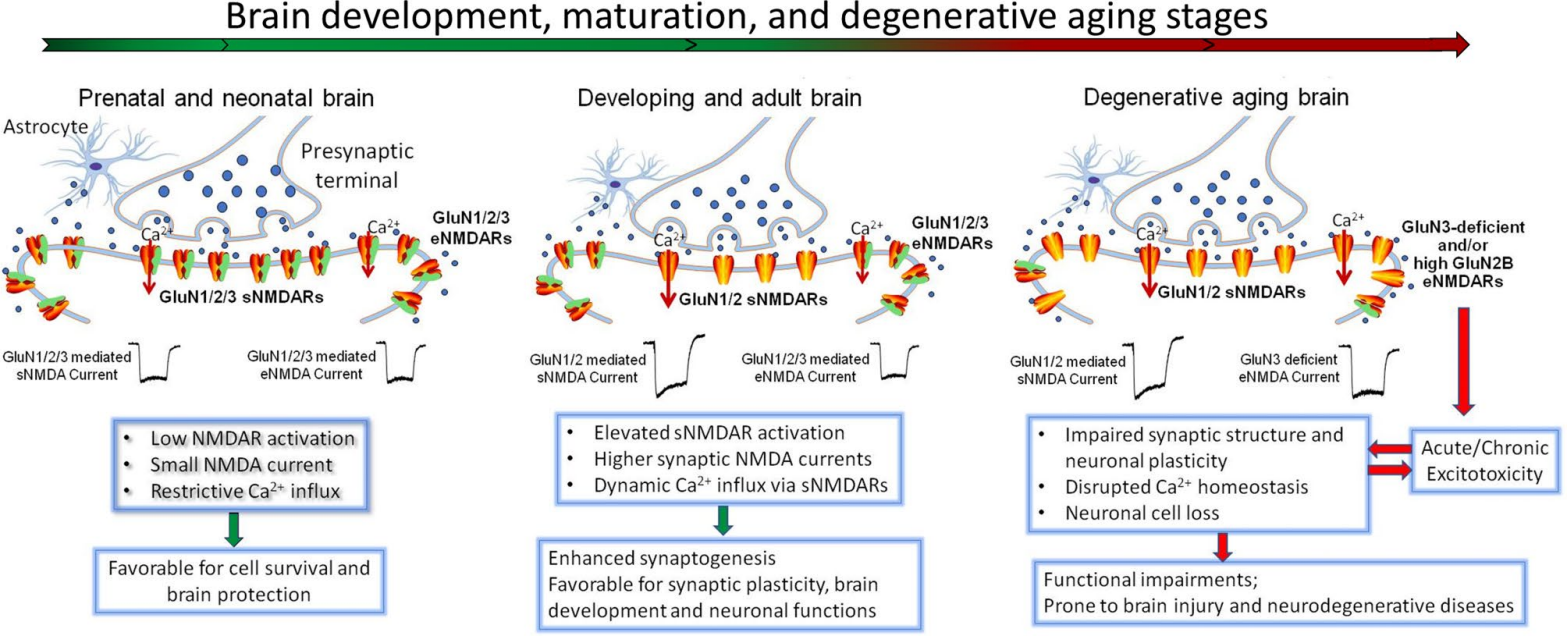
Age-dependent subunit alternations of synaptic and extrasynaptic NMDA receptors and functional consequences. NMDA receptors are mainly located in the post-synaptic membrane inside and outside of the synaptic cleft. Synaptic NMDARs are directly involved in excitatory neurotransmission and synaptic plasticity, while extrasynaptic NMDARs have regulatory roles in these activities. Glutamate concentrations are markedly different between the cleft and extrasynaptic spaces. Glutamate released by astrocytes and microglia (not shown) are likely the main components of extrasynaptic glutamate, together with that spillover from the synaptic cleft. The distribution and topography of NMDARs are subjected to age-dependent alterations. In addition to a developmental switch of increased GluN2A/GluN2B ratio [340, 341], the GluN3 expression also undergoes an age-dependent change, from the initial locations of both synaptic and extrasynaptic sites to extrasynaptic/peri-synaptic site in the mature brain. This developmental change is likely associated with the functional needs of NMDAR regulation at different life stages. For example, high levels of GluN3A in immature brains is neuroprotective; while in the adult brain, the absence of GluN3 in the synaptic site allows synapse maturation and elevated plasticity. In the adult brain, GluN3 remains to exist in the extrasynaptic membrane as an endogenous neuroprotective mechanism against brain damage and neurodegeneration. In the aging/aged or degenerative brain, loss or weakening of this regulatory mechanism due to either increased GluN2B expression or deficiency of GluN3 will lead to enhanced eNMDA activity and Ca^2+^ dysregulation, which aggravate acute and chronic excitotoxicity associated with ischemic stroke and late-onset AD.

NMDARs are located not only at synapses but also at extrasynaptic sites [79, 80] (Fig. 2). Synaptic NMDARs are enriched with GluN2A, while eNMDARs are more likely to contain GluN2B, GluN3A, GluN3B, or GluN2C/2D subunits [81, 82]. GluN3A and 3B are mostly associated with the perisynaptic site of the postsynaptic density (PSD) [83]. There have also been reports of pre-synaptic localizations of GluN1, GluN2, and GluN3B [84]. The control of NMDAR activity and Ca^2+^ influx is critical for the induction of long-term potentiation (LTP) and long-term depression (LTD), which are believed to be closely associated with synaptic plasticity and learning/memory functions [85–87]. Early studies suggested that synaptic GluN2A-containing NMDARs and extrasynaptic GluN2B-containing NMDARs are differentially linked to the generation of LTP and LTD, respectively [88]. More recent evidence implicates that extrasynaptic NMDARs act as regulators of both LTP and LTD [86, 89, 90]. There is also some evidence to support that GluN2C/2D containing eNMDARs can regulate synaptic currents and interneuronal/intrinsic excitability [91–95]. For example, eNMDARs can regulate neuronal and neural network activity in striatal neurons [96]. The current view of the contribution of NMDARs to brain physiology and pathology does not solely rely on a dichotomy between GluN2A- and GluN2B-containing NMDARs, or between synaptic and extrasynaptic NMDARs. Under certain conditions, both sNMDAR and eNMDAR may influence different aspects of synaptic plasticity [97, 98].

In neuronal cultures, a comparable [Ca^2+^]_i_ increase induced by the activation of eNMDARs, but not by sNMDAR activation, leads to mitochondrial dysfunction and cell death [79, 99, 100]. Evidence indicates that eNMDARs containing GluN2B are primarily related to excitotoxicity in stroke and neurogenerative diseases [99, 101, 102]. A few studies showed that GluN2D, likely in eNMDARs, contributes to excitotoxicity in retinal injury [103]. On the other hand, GluN2A-containing sNMDARs may play a role in excitotoxic cell death under certain experimental conditions [104–108]. For example, in hippocampal slices, the excitotoxicity induced by 50 µM NMDA for 30 min was attributed to sNMDARs based on the co-agonist D-serine and other pharmacological tools [87]. The discrepancy may be explained by the proposition that the balance between sNMDAR and eNMDAR activities is important in determining whether stimulation by NMDA or glutamate is neurotoxic or not [109]. It is more likely that drastic overactivation of both sNMDARs and eNMDARs accompanied by massive Ca^2+^ influx in vivo is a typical trigger of acute excitotoxicity commonly seen after ischemic strokes [110, 111]. Intriguingly, it was shown that GluN2C expression increased in hippocampal slices in response to ischemia; knocking out GluN2C exacerbated neuronal death in the CA1 area of the hippocampus and reduced spatial working memory compared to wild-type mice. In vitro and in vivo examinations revealed that GluN2C-expressing hippocampal neurons showed marked resistance to NMDA-induced toxicity and reduced Ca^2+^ influx, which is consistent with the notion that GluN2C-containing NMDARs exhibit a low Ca^2+^ permeability [112].

GluN3 is highly expressed in many subcellular compartments during early development and is particularly concentrated in PSD-associated perisynaptic and extrasynaptic locations [70, 113] (Fig. 2). This is supported by ultrastructural evidence that GluN3A is more abundant at perisynaptic and extrasynaptic sites in both juvenile and adult animals [113, 114]. This pattern of expression appears consistent with the function of GluN3 in constraining eNMDAR activity during early life to limit excessive Ca^2+^ influx, which is favorable for protecting the immature brain. The reduction of GluN3A later on during CNS development relieves the restriction on local Ca^2+^ upregulation and facilitates synaptogenesis, including the modulation of experience-driven synapse refinements [115]. Thus, the downregulation of GluN3A-containing NMDARs provides a developmental switch for activity-dependent maturation and stabilization of selected synapses, which are essential steps in synaptogenesis and memory consolidation [115]. Compared to the level in the neonatal brain, the total expression level of GluN3 is significantly reduced after brain development. Nevertheless, abundant GluN3 expression is still readily detectable in the adult brains of rodents as well as humans [116–120], suggesting that GluN3 has important functional roles in adult physiology and pathophysiology. Being a unique subunit affecting NMDAR activities, GluN3A exhibits functional roles in synaptic as well as extrasynaptic activities [121, 122]. The topography of GluN3 localization and activity are critically important across the lifespan. We propose that GluN3 acts as a tireless gatekeeper to prevent overactivations of eNMDARs and excessive Ca^2+^ influx, which is vital in maintaining Ca^2+^ homeostasis, cell viability, and normal neuronal functions (Fig. 2).

Some reports suggest that sNMDAR activation and eNMDAR activation contribute equally to excitotoxicity [123]. Alternatively, it was proposed that the subunit composition of NMDARs, such as the expression of GluN2B, but not the cellular location, is a determining factor for their effect on neuronal fate [124]. An imbalance between synaptic and extrasynaptic NMDA receptor activity could be a pathogenic factor for neurodegenerative diseases such as AD and Huntington’s disease (HD) [12, 107]. The disrupted balance may result from a malfunction or deficiency of an NMDAR subunit due to genetic mutation, mislocalization, or trafficking deficits of the subunit [125]. Imbalances may also be created by dysregulated glutamate concentrations in the vicinity of NMDARs. In this case, eNMDARs are directly responsible for glutamate excitotoxicity and cell death [79, 99, 100, 123]. For instance, the increased extrasynaptic composition of GluN2B-containing NMDARs or a deficiency of extrasynaptic GluN3A subunit induces enlarged tonic NMDAR currents that are closely associated with chronic excitotoxicity [80, 126, 127].

### Regulation of glutamate concentration in the extrasynaptic space and excitotoxicity

As the primary excitatory neurotransmitter in neuronal communications, the glutamate concentration in the synaptic cleft is highly dynamic and tightly controlled, with rapid rises and falls (in milliseconds) within the µM to mM range to convey neuronal activity [128–131]. Glutamate is not restricted to the synaptic cleft; it also exists in the extrasynaptic space due to the spillover from the presynaptic release and secretion of glutamate by adjacent astrocytes and microglia [132–135] (Fig. 2). According to early assessments, the basal concentration of extrasynaptic glutamate is sustained at low µM levels [131, 136–139]. This might be an overestimation of the resting concentration; improved measurements in acute hippocampus slices have assessed the concentration to be approximately 100 nM [140, 141].

Studies on mixed cell cultures of neurons and astrocytes show that the glutamate concentrations that can lead to acute cell death (in hours to a few days) are in the high µM range (EC_50_ = 205 µM) [142]. This range appears too high for the extrasynaptic glutamate to reach in a persistent manner. On the other hand, sustained glutamatergic hyperactivity and Ca^2+^-associated chronic excitotoxicity are clearly identified in neurodegenerative diseases, such as AD. At present, little is known about the relationship between the glutamate concentration threshold and the duration of elevated extrasynaptic glutamate for inducing the slowly developed degenerative excitotoxicity underlying the prolonged process of neurodegeneration.

The extrasynaptic glutamate level is sensitive to abnormal and pathological conditions. For example, in rodents, stressful stimuli such as body restraint, forced swimming, and hypoxic insults can selectively increase the extrasynaptic glutamate concentration to over 30 µM or higher [143–145]. In stroke and brain injuries, the reduced extracellular volume associated with brain edema elevates the extrasynaptic glutamate concentration as a contributing factor in excitatory neuronal damage [146]. Extrasynaptic glutamate is taken up by neurons and astrocytes mostly via excitatory amino acid transporters (EAATs) and is metabolized in astrocytes to glutamine [147]. Multiple alterations of cellular and subcellular activities may cause increases in extrasynaptic glutamate. Among them, aberrant burst firing of presynaptic inputs and reduced glutamate clearance due to lower EAAT1-3 activity at depolarized membrane potentials can be the primary causes of increased extrasynaptic glutamate [146]. Other sources of increased extrasynaptic glutamate may include reversed operation of glutamate transporters [146].

In AD pathophysiology, deficient glutamate uptake and recycling contribute to elevated levels of extrasynaptic glutamate [148, 149]. Studies in neuronal cell cultures demonstrated that various species of Aβ peptides caused increased glutamate availability via their deteriorating effects on glutamate transporters [150–152]. It was proposed that Aβ-induced excitotoxicity is mediated by increasing extracellular glutamate concentrations due to decreased glutamate uptake from the synaptic cleft, which is correlated with impaired function of EAAT2 in perisynaptic astrocytes. Glutamate release from glial cells such as astrocytes and microglia [153] and decreased recycling of glutamate [154] may also contribute to chronic excitotoxicity.

The role of interplay between astrocytes and neurons has been further strengthened through recent work in human and rodent transcriptome analysis indicating weakened metabolism coordination between these cells under neuropathological conditions. In particular, reactive glia were less evident in TREM2-R47H and TREM2-R62H carriers than in noncarriers, implicating TREM2 and glia-neuron interactions in both mouse and human AD [155, 156]. In AD patients, decreased expression and capacity of glutamate transporters, specifically a selective loss of vesicular glutamate transporter (vGlut), were detected [154, 157]. Moreover, EAAT2 located in perisynaptic astrocytes displayed malfunction in the AD brain [158]. These increasing observations align with the proposition that enhanced glutamate levels in the extrasynaptic space are an important contributor to slowly developed excitotoxicity and neurodegeneration in AD development.

### Glutamatergic hyperactivity in ischemic stroke and AD

Upon an acute ischemic attack, the sudden reduction in cerebral blood flow causes dramatic consequences in the affected region within minutes to a few hours, including a loss of oxygen supply and disrupted mitochondrial function, ATP deficiency, and membrane depolarization, which collectively trigger augmented glutamate release and impaired uptake [146, 159]. The overall increase in glutamate concentration leads to the overactivation of glutamate receptors at synaptic and extrasynaptic sites of excitatory neurons, causing massive Ca^2+^ influx and ionic homeostasis disruption, cell swelling, and cell membrane deterioration, all of which are characteristics of necrotic cell death [31, 160, 161]. Cells surviving the initial ischemic insult may die a few days or weeks later in a “hybrid” form due to the activation of programmed cell death and aberrant autophagy pathways [162].

In addition to its well-documented roles in ischemia/hypoxia, eNMDAR activation has been implicated in the pathogenesis of neurodegenerative disorders, especially morphological and functional deteriorations in AD [153, 163, 164] and HD [109, 165]. Neuronal and NMDAR hyperactivity during the progression of AD and related dementia has regularly been detected in animal AD models as well as in AD patients [50, 51, 166, 167]. This abnormality occurs during the early stages of late-onset AD development [11, 12, 168]. The mechanism of action for the lasting time course and pathogenic effect of NMDAR hyperactivity, however, has been poorly defined. Intriguingly, brain hyperexcitability may not depend on amyloid plaque formation [169]. The sustained trigger of NMDAR hyperactivity at the initial stage of sporadic AD before endogenous Aβ deposition is largely unknown. Moreover, instead of hyperactivity, impaired NMDAR activity and signaling in the cortex and hippocampus can be observed in the aging/aged AD brain [170, 171]. This is a possible consequence of neurodegeneration as a result of chronic excitotoxicity and Aβ aggregation. In AD research, while the Aβ hypothesis has been demonstrated in different amyloid transgenic mouse models, basic research on the triggering and mediating mechanisms of neuronal toxicity has been solely focused on the toxic effects of amyloid and tau pathology. As a result, neuronal hyperactivity and NMDAR overactivation are attributed as consequences of the amyloid cascade both in vitro and in FAD transgenic mice [172–175]. This popular concept, however, has been challenged by compelling preclinical and clinical observations that neuronal and NMDAR hyperactivity can often be observed in the absence of amyloid deposition [33–36, 169].

### Activation of eNMDARs and tonic NMDAR currents

In contrast to the phasic and intensive activation of sNMDARs, eNMDARs are associated with tonic NMDA currents induced by lower concentrations of extracellular glutamate. Consistent with their extrasynaptic locations, eNMDAR-mediated currents are insensitive to tetrodotoxin (TTX), which selectively blocks the synaptic release of glutamate [176, 177]. In line with their nonsynaptic nature, eNMDARs can be constitutively activated, and the activation may persist even in the absence of neuronal and/or synaptic activity [178]. In contrast, sNMDAR activity remains unaffected when tonic NMDA currents are blocked [176]. These characteristics clearly define two populations of NMDARs, one located inside and one located outside of the synaptic cleft.

Compatible with the lower concentrations, extrasynaptic glutamate preferably activates the main population of eNMDARs of higher affinity, e.g., GluN2B-containing NMDARs. GluN2B-containing eNMDARs were proposed to be responsible for ischemia-induced excitotoxicity [179, 180], and extrasynaptic glutamate is a primary contributor to ischemic and traumatic damage in the brain [143, 146, 181]. The activation of eNMDARs, perhaps together with the impaired protective function of sNMDARs, contributes to downstream cascades of necrotic and programmed cell death pathways [179, 182]. The tonic NMDA current is likely triggered by glutamate released mainly from astrocytes, which participate in neuron-glia communications, such as the regulation of neuronal excitability and synaptic strength that subsequently affect learning and memory formation [80, 134, 178]. The cellular properties of excitability and synaptic strength can be a part of neuroendocrine regulation as well as neuromodulatory actions or even sleep homeostasis [147].

Synaptic NMDAR activity is closely coupled to glutamate released from presynaptic vesicles and the clearance of glutamate from the synaptic cleft, while eNMDAR activation is characterized by exposure to chronic agonism by surrounding glutamate [183]. Ca^2+^ influx evoked by intense sNMDAR activation alone may not be harmful, as it can trigger genomic processes that render neurons more resistant to apoptotic and oxidative insults. It was shown, however, that long-term tonic activation of sNMDARs in hippocampal neurons under hypoxic conditions was able to induce excitotoxic cell death [104]. Thus, prolonged sNMDAR activation may also trigger pro-death signaling. In cultured cortical neurons, long-term, but not short-term, treatment with high-dose NMDA or oxygen-glucose deprivation triggered cell death and suppressed prosurvival signaling. The authors proposed that the co-activation of sNMDARs and eNMDARs is needed for excitotoxicity [106]. It is likely that any shift in balance to reduce sNMDAR or enhance eNMDAR signaling could be detrimental to neuronal viability [184, 185]. Thus, the NMDAR signaling inside and around the synapse must be maintained at a proper level so that it is enough to maintain neuronal activity and viability but not enough to become harmful so as to cause excitotoxic neurodegeneration [79, 154, 183].

Taken together, a large body of evidence supports the existence of a critical role for enhanced eNMDAR activity in the pathogenesis of stroke and AD [107, 108]. While the NMDAR localization hypothesis is not universally accepted, the majority of experimental data support that the stimulation of eNMDARs is a common early feature of acute and chronic neurological disorders. Acute and chronic excitotoxicity in stroke and neurodegenerative diseases, however, show distinct features in terms of glutamate intensity, time course, signaling pathways, and cell death mechanisms. Targeting eNMDARs can be a promising target for developing safe and effective therapies for these neurological disorders by decreasing extracellular glutamate spillover/release and tonic eNMDAR activation and ultimately maintaining the balance of synaptic and extrasynaptic NMDAR signaling under stressful and pathological conditions.

### Distinct signaling pathways associated with synaptic and extrasynaptic NMDARs

**Fig. 3:**
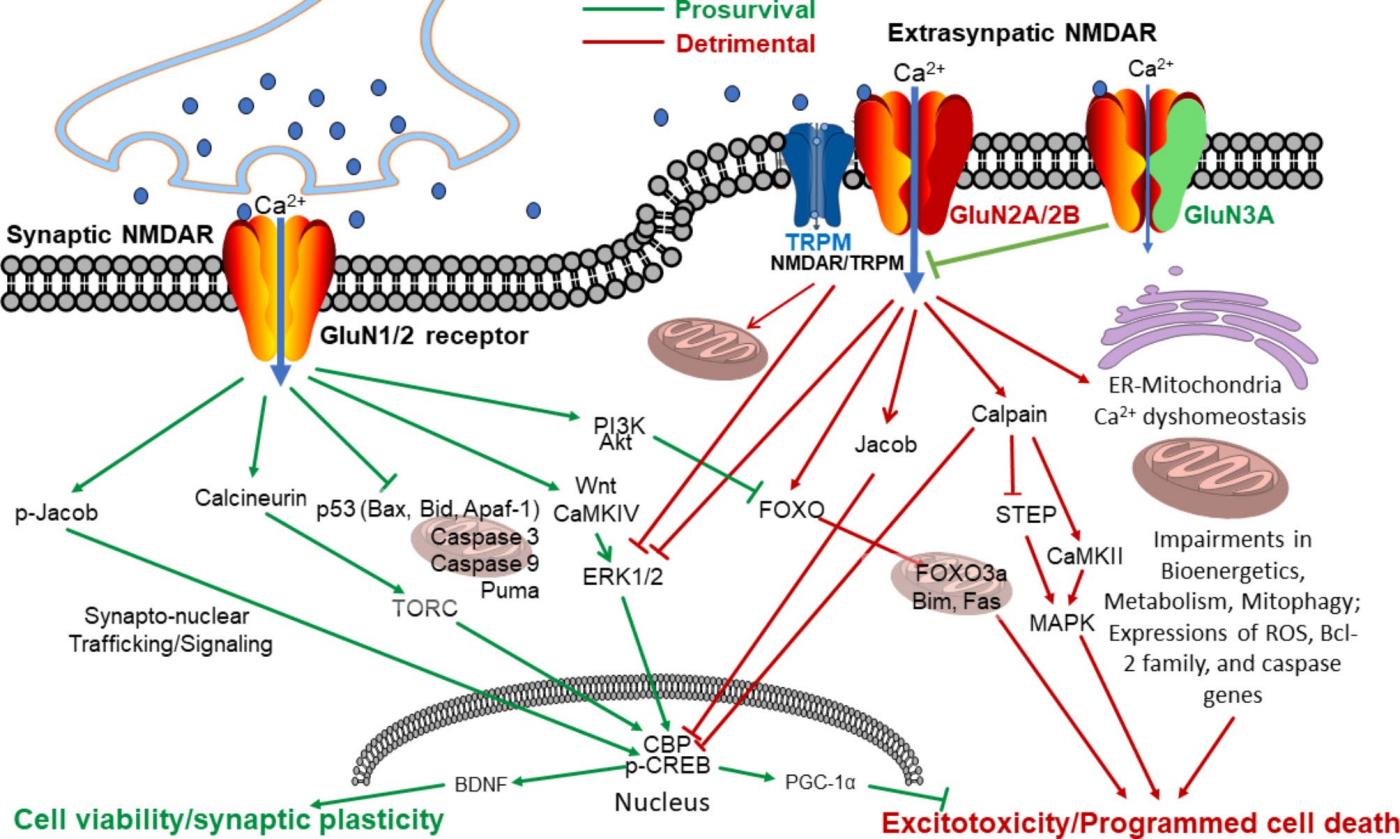
Distinctive Pro- and anti-survival mechanisms downstream from activation of synaptic and extrasynaptic NMDARs. This simplified graph illustrates a few main signaling pathways associated with the activation of synaptic and extrasynaptic NMDA receptors, respectively. Of note that although additional genes not mentioned in the text are shown in the graph, not all related signals can be included in the graph. For example, chronic stress of neuronal hyperactivity and Ca^2+^ elevations induce recurrent inflammation that is not shown here. In general, activation of sNMDARs leads to pro-survival effects beneficial for neuronal viability and synaptic plasticity, while activation of eNMDARs causes detrimental consequences associated with acute and chronic excitotoxicity. It is worth mentioning, however, that many signaling genes such as CaMK and MAPK kinases can play opposite actions most likely in subtype-dependent manners. In the pro-survival mechanism, the Wnt regulation of the expression of CaMKIV is an upstream protective signaling in neurodegenerative conditions [342]. Cyclic-AMP response element binding protein (CREB) plays a key function in medicating sNMDAR activation and expressions of pro-survival genes such as BDNF, MAPK, and Akt. CREB phosphorylation is mediated acutely by CaMKIV while long-term regulation may be controlled upstream by ERK1/2 [343, 344]. Activation of CREB via CaMKIV phosphorylation of CREB binding protein (CBP) requires translocation of transducer of regulated CREB activity (TORC) which is downstream of Ca^2+^ signaling from sNMDAR activation. Jacob and the synapto-nuclear trafficking is a relatively new mechanism linking downstream signaling of sNMDARs. Caldendrin binds to Jacob’s nuclear localization signal in a Ca^2+^-dependent manner [194, 195]. In contrast to these CREB-activating signals of sNMDARs, eNMDARs suppress CREB activity via the inactivation of the Ras-ERK1/2 pathway and the nuclear translocation of Jacob, which promotes CREB dephosphorylation. Calcineurin-dependent dephosphorylation of TORC and subsequent CREB activation is also downstream of sNMDAR transmission [345]. Activation of sNMDARs suppresses apoptotic cascades via suppression of the BH3-only domain gene Puma and p53, thereby limiting cytochrome c release. Downstream effectors of apoptosis including Apaf1, Caspase 3, and Caspase 9 are also suppressed [346, 347]. Contrary to these pro-survival pathways, pro-death pathways are mediated by downstream activities of eNMDARs [189, 348]. Interactions between the pro-survival and pro-death pathways may occur so that the suppression of CREB activity may result from inactivating the ERK1/2 pathway [79, 195]. Another shared pathway between synaptic and extrasynaptic NMDARs is the FOXO pathway. FOXO activity is suppressed by PI3K downstream of sNMDARs while activation of eNMDARs enhances FOXO nuclear import and the consequent transcription of FOXO3α, Bim, and Fas which lead to cell death via multiple mechanisms including excitotoxicity [205, 349]. Synaptic NMDAR activity enhances the transcription of PGC-1α, while excessive expression and activity of eNMDARs suppress CREB-dependent PGC-1α transcription [350]. In general, CaMKII is downstream of eNMDAR activity and acts as a carrier of Ca^2+^-regulated protease calpain to promote apoptotic cell death [351]. Moreover, Ca^2+^ dyshomeostasis resulting from NMDAR subunit composition such as GluN2B and GluN3A expression changes and its interaction with intracellular Ca^2+^ reservoirs in the ER and mitochondria play an important role in the maintenance of cellular bioenergetics, glucose metabolism, and normal mitophagy [352]. Ca^2+^ dyshomeostasis is thus a major trigger of the generation of ROS and increased apoptosis via the imbalance of mitochondria-initiated apoptotic genes including tBid, Bax/Bcl2, Bak/BclxL, Bad, Apaf1, cytochrome *c*, and caspases [353, 354]. The NMDAR-TRPM interaction is a novel cell death mechanism downstream to NMDAR and TRPM activation, which stimulates the formation of the NMDAR/TRPM complex in the extrasynaptic location. Excitotoxicity is then triggered by the complex in a “Ca^2+^-independent” fashion, mediated by mitochondrial dysfunction, reduced activation of ERK1/2, shut-off of the transcription factor CRAB, and cell death [229]

**Fig. 4:**
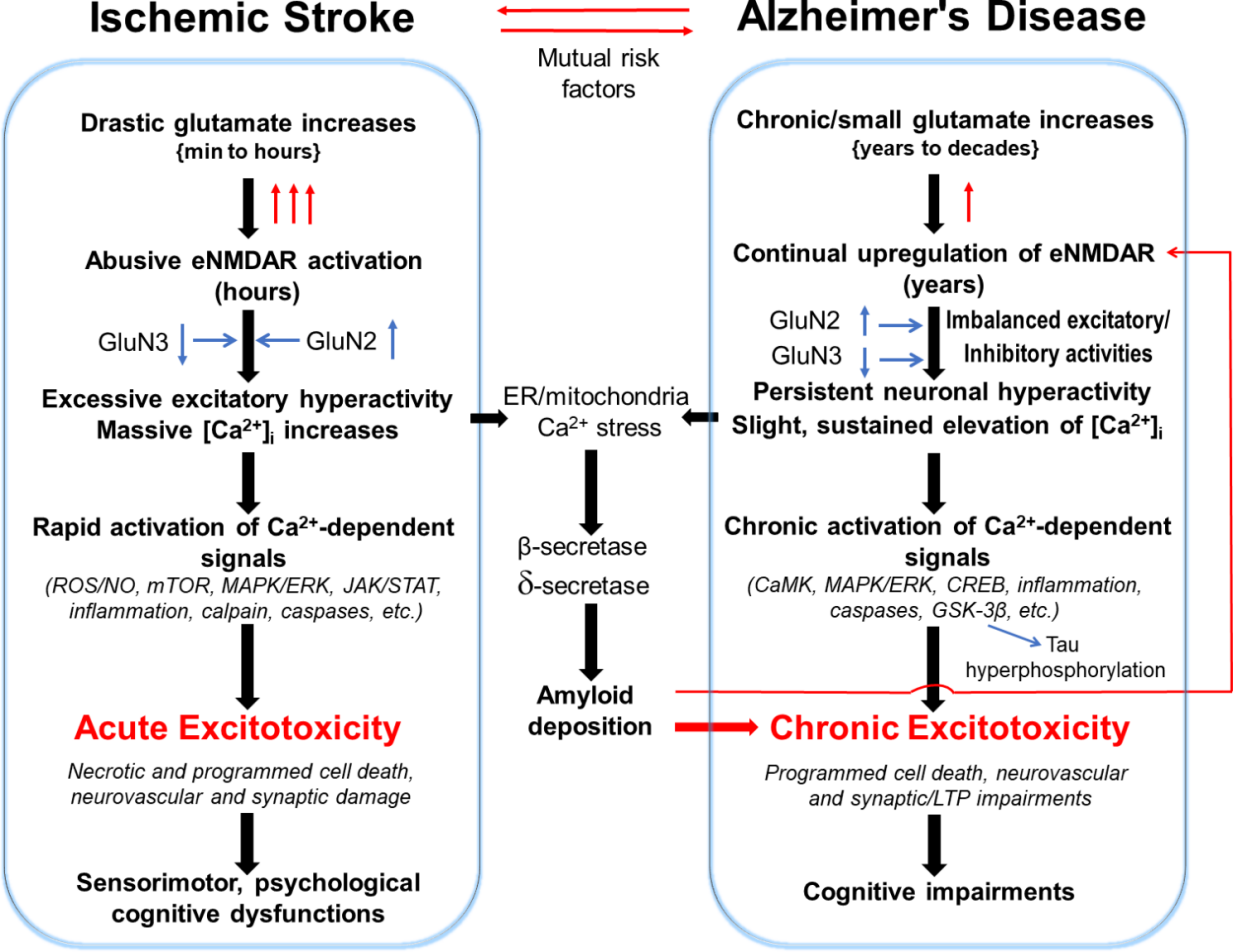
Acute and chronic excitotoxicity and shared mechanisms between ischemic stroke and sporadic AD. The sketch diagram illustrates similarities and differences between ischemic stroke and AD. Both brain disorders suffer from overactivations of eNMDARs that are subjected to regulations by glutamate concentration, expression of NMDAR subunits (e.g. GluN2 and GluN3), and other modulatory mechanisms. The vast and rapid Ca^2+^ influx upon cerebral ischemia and much mild but lasting Ca^2+^ stress in AD trigger distinctive Ca^2+^-dependent signaling pathways, leading to acute and chronic excitotoxicity, respectively. Depending on the severity and region of damage, ischemic stroke causes transient or permanent deficits of locomotor/sensorimotor activities and psychological/psychiatric/cognitive functions. Cerebral ischemia is also known for causing mitochondria dysfunction and ER Ca^2+^ stress that may be responsible for post-stroke AD-like pathology. On the other hand, chronic excitotoxicity in AD is induced by long-lasting small Ca^2+^ increases and deteriorating signaling pathways that lead to synaptic and neural network interruptions in specific regions critical for cognition, followed by Aβ deposition via increased activities of β- and δ-secretases [355]. This chronic excitotoxicity may cause late-onset AD in Aβ-dependent or -independent manner, which remains to be further investigated

The fact that different NMDAR subtypes locate at synapses and extrasynaptic sites raises the question of whether specific NMDAR subtypes and locations are responsible for distinct functions. Indeed, synaptic GluN2A-containing NMDARs are generally associated with cell survival, whereas extrasynaptic GluN2B-containing NMDARs are linked to cell death cascades [186, 187]. The activation of sNMDARs is related to the transcription of pro-survival genes and anti-apoptotic genes, which are favorable for cell survival through the phosphorylation of intracellular factors such as the transcription factor Cyclic-AMP response element binding protein (CREB) [188, 189]. Consistent data suggest that ERK1/2 is activated and inactivated by sNMDARs and eNMDARs, respectively [190]. The Ca^2+^ influx mediated by sNMDARs leads to consequent Ca^2+^ release from internal stores, generating Ca^2+^-activated kinases and transcription factors in the nucleus, such as Ca^2+^-calmodulin kinase IV (CaMKIV) and CREB, while inhibiting the transcription factor FOXO3α [189, 191]. The L-type voltage-gated channel is another main player in mediating the Ca^2+^ elevation in this signaling pathway [192]. The transcription induction results in the expression of brain-derived neurotrophic factor (BDNF) [189]. The activation of sNMDARs also increases Wnt/MAPK/ERK1/2 survival signaling activity [190, 193] and PI3K/Akt activity to promote the inhibition of FOXO [191]. JACOB is a caldendrin binding partner and a synapto-nuclear signaling protein [194]; ERK1/2 and the phosphorylation of JACOB appear to play a major role in communicating the origin of NMDAR activity to the nucleus, referred to as the synapto-nuclear trafficking/signaling [194, 195] (Fig. 3). On the other hand, the malfunction of extrasynaptic glutamate signaling is an important contributor to several pathophysiological conditions, including hyperexcitability, spreading depression, neurodegeneration, neuroinflammation, and demyelination [196]. The activation of eNMDARs leads to cell death by inhibiting survival signaling as well as promoting pro-death mechanisms such as the expression of cleaved caspase-3 [153, 197], the suppression of the CREB, p38 MAPK, and ERK1/2 pathways [190, 198], the activation of the FOXO transcription factor associated with AD neuropathology [191], and calpain-mediated cleavage of striatal-enriched protein tyrosine phosphatase (STEP) [79, 195, 199–201] (Figs. 3 and 4).

Glutamate-induced activation of eNMDARs in cultured neurons is required to disrupt the mitochondrial membrane potential associated with excitotoxic injury [79], which is likely mediated by GluN2B-containing eNMDARs [100]. Mounting data indicate that both NMDA receptor dysfunction and mitochondrial impairment are present in AD patients, animal models, and cell culture models. In neurons, Aβ and altered NMDAR function are linked with mitochondrial dysfunction through the dyshomeostasis of mitochondrial Ca^2+^ following Ca^2+^ influx mediated by GluN2B-NMDARs [202]. NMDAR-related mitochondrial dysfunction leads to increased production of reactive oxygen species (ROS), altered Ca^2+^ homeostasis, and decreased ATP production, providing a pathological link between eNMDARs, metabolism, and neurodegeneration [154, 203, 204] (Figs. 3 and 4). On the other hand, sNMDAR activity is necessary to boost intrinsic antioxidant defenses, which may further explain its neuroprotective effect against the progression of pathological processes associated with oxidative damage [205].

### Ca^2+^-associated excitotoxicity in ischemic stroke and AD

Ca^2+^-induced excitotoxicity was initially described in neuronal cultures and animal models of ischemic stroke in investigations of glutamate-induced neuronal cell death [111, 206–208]. This type of cell death was characterized by drastic activation of glutamate receptors, mainly NMDARs, by excessive amounts of extracellular glutamate due to augmented synaptic release and impaired uptake mechanisms [209, 210]. The overstimulation of NMDARs results in massive Ca^2+^ influx and [Ca^2+^]_i_ overload [211, 212], and acute neuronal injury featured by necrotic cell death [110, 117, 213] (Fig. 3). Consistent evidence has shown that Ca^2+^ entry through NMDARs was particularly more effective at killing neurons than entry through other receptors and channels [111, 146, 159, 160, 207, 208, 214]. Continual investigations also identified that, in conjunction with necrotic damage, programmed cell cascades such as apoptosis, aberrant autophagy, and the activation of several other cellular death mechanisms could take place concurrently or consequently as a part or consequence of excitotoxicity [162, 215–217].

**Fig. 5:**
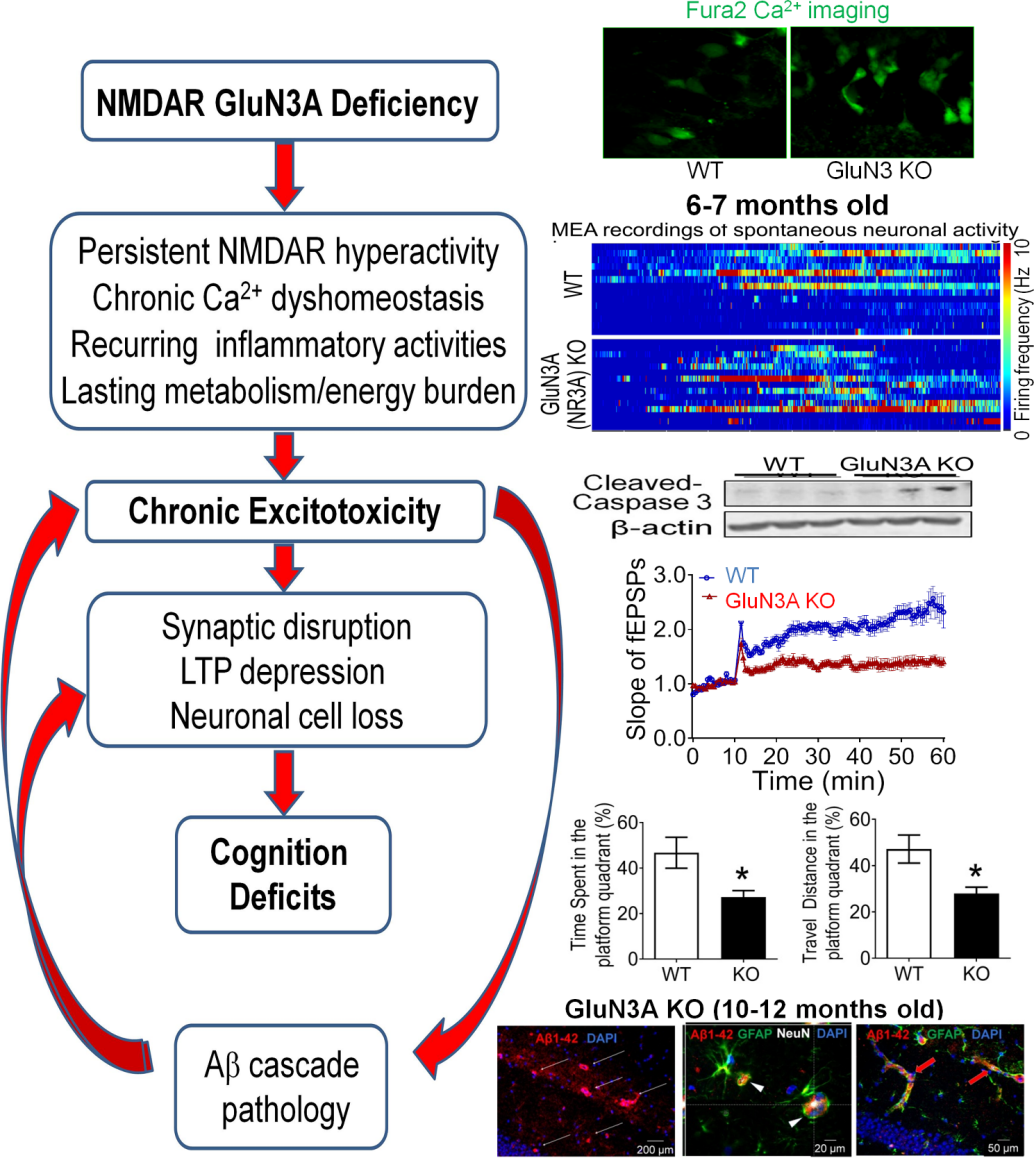
NMDAR GluN3A deficiency induced sporadic AD. The graph shows age-dependent events and corresponding experimental evidence in the NMDAR GluN3A knockout mouse. A GluN3A deficiency caused by genetic mutation or functional dysfunction can result in slight but persistent neuronal hyperactivity and [Ca^2+^]_i_ elevations, subsequently leading to chronic inflammation, metabolism burden, and slowly evolved degenerative excitotoxicity. The synaptic impairment and programmed neuronal cell death in the hippocampus and cortex are correlated to progressive cognitive decline. Interestingly and important to note that significant endogenous Aβ plague formation in neurons and blood vessels occurs after, but not before, cognition decline and other functional deficits [127]

In neurogenerative diseases such as AD and HD, no detectable acute excitotoxicity exists, while delayed and continuous neuronal loss are the hallmarks of neurodegeneration in those patients’ brains [218]. To explain the pathophysiology of chronic neuronal degeneration, the Ca^2+^ hypothesis proposed that moderate yet persistent [Ca^2+^]_i_ increases can cause excitotoxic neuronal damage in neurodegenerative diseases [127, 219–221]. Accumulating evidence supports that AD pathophysiology includes a chronic “calciumopathy” caused by NMDAR overactivation and Ca^2+^-induced excitotoxicity [11, 12, 168]. It was proposed that small but sustained increases in [Ca^2+^]_i_ activate Ca^2+^-dependent deleterious signals as key events or even causal factors of AD development [11, 12, 168, 222] (Figs. 4 and 5). The long-lasting (months in rodents and years/decades in humans) excitatory stresses are associated with not only increased Ca^2+^-activated signaling pathways but also chronic metabolic and inflammatory burdens, slowly progressing neuronal loss, and ultimately morphological and functional deterioration, including cognitive symptoms (Figs. 4 and 5). In line with this cascade, CREB phosphorylation at serine 133, which is required for its transcriptional activity and cell survival, is decreased (“shut-off” of the CREB signaling) after eNMDAR stimulation and in AD [79, 200]. At present, how NMDAR activity is persistently upregulated in the seemingly normal brain and its precise link to AD progression are poorly understood. Current research in this area has exclusively examined the NMDAR GluN1 and GluN2 subunits. For example, an increase in GluN2B expression was attributed to Aβ-induced NMDA hyperactivity [223].

Our recent investigation demonstrated for the first time that the deficiency of GluN3A plays a life-long pathogenic role in causing mild but persistent activation of eNMDARs, chronic Ca^2+^ dyshomeostasis, and degenerative excitotoxicity [127], as seen in clinical late-onset AD/ADRD patients [127, 224] (Fig. 5). GluN3A knockout (KO) mice age-dependently developed virtually all pathophysiological features of neurodegeneration and AD pathology, including programmed cell death signaling activation, chronic neuronal loss, synaptic disruption, LTP suppression, and early symptoms of olfactory deficits followed by progressive psychological and cognitive deficits [127]. In contrast to current FAD models and support an Aβ-independent AD mechanism, endogenous amyloid deposition and tau hyperphosphorylation spontaneously emerged in the GluN3A KO brain after, but not before, cognitive decline [127] (Fig. 5). Additionally, our recent study verified that selective knockout of GluN3A in the cortex and hippocampus of young adult mice (3 months old) using the CRISPR/Cas9 method resulted in similar AD-like morphological and functional alterations 3–6 months later [225]. The expression (knock-in) of GluN3A in the global GluN3A KO brain effectively prevented AD progression [225]. We also showed that early MEM treatment in GluN3A KO mice of 3-month old before cognition decline and Aβ deposition significantly prevented the age-dependent AD development [127]. Based on observations in this novel late-onset sporadic AD model, we propose that neuronal hyperactivity mediated by chronic up-regulation of eNMDAR activity, slight but persistent [Ca^2+^]_i_ increases and resulted chronic excitotoxicity in the cortex and hippocampus are a causal pathogenic mechanism of AD/ADRD (Fig. 5).

### A novel mediatory pathway downstream to NMDAR activation and Ca^2+^ increases

Transient receptor potential cation channel subfamily M members (TRPMs) have been identified as key modulators of numerous Ca^2+^-dependent mechanisms such as the immune response, insulin secretion, myogenic tone of the cerebral artery, capillary fragmentation, and respiratory rhythm regulation [226]. Among them, TRPM4 is a Ca^2+^-activated monovalent cation channel [227, 228]. More recently, an interaction between NMDARs and TRPM4 was identified in NMDA-induced acute excitotoxicity. It is proposed that excitotoxicity requires the physical interaction of NMDARs and TRPM4, via intracellular domains in the near-membrane portions of the receptors [229]. The disruption of the NMDAR/TRPM4 complex does not affect NMDAR-mediated [Ca^2+^]_i_ increases, suggesting that the NMDAR-TRPM4 complex does not affect NMDAR activity or Ca^2+^ permeability, rather it influences downstream events following NMDAR activation. Meanwhile, blocking NMDAR-TRPM4 interactions reduces NMDA toxicity and mitochondrial dysfunction, activates CREB and ERK1/2, boosts gene induction, and reduces neuronal loss in mouse models of stroke and retinal degeneration [229]. Interestingly, the NMDAR-TRPM2 coupling promotes the surface expression of eNMDARs, ultimately leading to increased neuronal death [230], while MEM treatment modulates TRPM2-induced excessive [Ca^2+^]_i_, hypoxia-mediated ROS production, and apoptosis [231]. It was also proposed that multiple TRPM2-mediated cellular and molecular mechanisms cause Aβ and/or oxidative damage in AD pathologies [232]. The novel mechanism of neuronal death shines a light on a regulatory pathway downstream NMDAR activation, providing a possible neuroprotective strategy against excitotoxicity without directly blocking NMDARs [230].

### Interactions between NMDAR and Aβ: alternative consequences of pathogenic events

According to the amyloid hypothesis of AD, Aβ-triggered neuronal hyperactivity and NMDAR abnormalities have been extensively investigated for many years. For example, Aβ1–42 increased NMDAR-mediated Ca^2+^ influx [233, 234], and extracellular Aβ oligomers can bind to NMDARs containing the GluN2B subunit and mGluR1, consequently leading to synaptic disruptions [235, 236]. The Aβ effect was mediated by interaction with NMDARs, either directly or via synaptic proteins such as PSD95 [154, 237–239]. Aβ can increase NMDAR activity, Ca^2+^ influx, and Aβ-associated synaptic loss [240–242]. In transgenic AD animal models, NMDAR hyperactivity occurs following Aβ accumulation [243]. Aβ may directly activate GluN2A-containing NMDARs [244], and Aβ oligomers can evoke [Ca^2+^]_i_ rise through activated NMDARs in cortical neurons [233]. Numerous studies have shown that soluble Aβ causes a reduction in synaptic glutamatergic transmission and inhibits synaptic plasticity. For example, applying Aβ_1–42_ in cultured cortical neurons leads to the internalization of sNMDARs and the depression of NMDAR-mediated currents [245]. Aβ can also stimulate glutamate release from glial cells and activate eNMDARs [153]; glutamate release from cultured microglia and astrocytes were significantly greater in Aβ-treated cultures [246]. Detailed information about the effects of Aβ on NMDARs and neuronal activity can be found in several excellent reviews [54, 244, 247–249].

Extrasynaptic NMDAR activity promotes tau protein overexpression in neuronal cultures, and tau ablation is protective against cell death mediated by eNMDARs [250]. The promoting effect on tau phosphorylation is mediated by various kinases linked to augmented tau toxicity [153, 251]. Glycogen synthase kinase 3β (GSK-3β) is a tau kinase that is activated by Aβ [252] and contributes to Aβ-induced tau phosphorylation and toxicity [253]. Exacerbated tau toxicity associated with Aβ-induced GSK-3β activation can be prevented by inhibiting GluN2B-containing NMDARs [253]. Consistently, the Aβ impairment of axonal transport is significantly attenuated by NMDAR antagonists or by GSK-3β inhibition [254], supporting a functional link between NMDARs and GSK-3β activation. Interestingly, a recent study in tau knockout mice revealed that the deletion of tau decreased the eNMDA current in hippocampal neurons [255], which is consistent with the pro-degenerative roles of tau and eNMDARs via a similar receptor mechanism in AD.

More recent evidence suggests that sustained eNMDAR activation acts as an upstream event to Aβ production and secretion [241, 242] (Fig. 5). For example, overall NMDAR activation by bath NMDA application to cortical neuronal cultures increased the production and secretion of Aβ by upregulating the expression of APPs [256]. Synaptic activity and vascular exocytosis in the hippocampus drive the release of Aβ into the extracellular space [257], though the work did not identify involved NMDAR subtypes. In primary cultures of wild-type cortical neurons, prolonged stimulation of eNMDARs, but not sNMDARs, significantly increased the neuronal synthesis and release of Aβ [163]. This effect was preceded by a shift from APP695 (the neuronal isoform of APP) to KPI-APPs, which are isoforms exhibiting important amyloidogenic potential. The authors suggested that the eNMDAR pool is associated with APP and Aβ metabolism. Supporting this idea, there are significant overlaps between the signaling molecules implicated in AD and those influenced by eNMDAR stimulation. The effect of Aβ on JACOB translocation is entirely blocked by the GluN2B-specific antagonist ifenprodil, implying a mediating role of eNMDARs [258]. BDNF release from the implanted cells can attenuate cognitive deficits in AD mice, suggesting that BDNF deficiency may play an essential role in AD pathophysiology [259]. Furthermore, it was shown that BDNF induction is suppressed by eNMDAR activity [200]. Additionally, BDNF release from astrocytes is known to regulate the hyperactivation of neuronal populations via TrkB [260]. Importantly, this functional benefit of BDNF is achieved without improvement in either Aβ or tau pathology [259], suggesting a BDNF-dependent action downstream of the Aβ and tau cascade or an AD/tau independent mechanism. Endorsing these possibilities, viral delivery of CREB-binding protein (CBP) increases BDNF expression and improves cognitive function in an AD model without affecting Aβ or tau pathology [261].

As an underlying mechanism, sNMDAR activity increases α-secretase-mediated nonamyloidogenic processing of APP [262], while Ca^2+^ influx via persistent activation of eNMDARs leads to intranuclear CaMKIV activation and, via a series of signaling cascades, results in a shift from α-secretase to β-secretase-mediated APP processing and thereafter an increase in Aβ production [101]. It was further postulated that eNMDAR-promoted production of Aβ creates a toxic positive feedback loop in which Aβ enhances eNMDAR activity and stimulates Aβ production and secretion [107]. Collectively, accumulating evidence implicates that eNMDAR activation and imbalanced sNMDAR/eNMDAR activity tone are possible causal factors acting upstream of late-onset AD pathophysiology, including Aβ and tau pathology (Figs. 4 and 5).

### MEM is an eNMDAR antagonist with neuroprotective properties and few side effects

For many years, the NMDAR contribution to excitotoxicity has represented attractive therapeutic targets for various CNS disorders. However, NMDAR antagonists that show promise in blocking excitotoxicity also disrupt normal synaptic functions, resulting in unacceptable side effects [30, 32, 263]. For instance, conventional NMDAR antagonists MK-801, phencyclidine (PCP), and ketamine induce some deteriorating actions such as schizophrenia-like symptoms in humans [264–266].

MEM is an uncompetitive low-affinity and use-dependent NMDAR antagonist with unique voltage and Mg^2+^ dependency that acts only at moderate depolarization [20, 52, 267, 268]. More importantly, unlike classic NMDAR antagonists, MEM preferentially acts on extrasynaptic GluN2B and GluN2C/2D containing NMDARs over synaptic NMDARs [99, 183, 269]. This is true, especially at therapeutic doses (1–10 μm in vitro and 1–30 mg/kg in vivo). Using Ca^2+^ imaging, it was demonstrated in primary cortical cultures that MEM significantly blocked the [Ca^2+^]_i_ increases mediated by eNMDARs and attenuated NMDA-induced neuronal cell death [99]. These unique pharmacological features of MEM imply minimal influence on the physiological activity of sNMDARs with effective suppression of overactivated eNMDARs.

MEM shows neuroprotective and neuroplasticity effects when it is administered acutely and chronically in stroke animals. In experimental acute treatments after permanent or transient ischemic stroke, MEM showed neuroprotective effects around 10–30 mg/kg (i.p., i.v. or oral) [270–273]. MEM can reduce ischemia-induced infarct formation and neuronal cell death acutely after an ischemic attack [271, 274]. In middle cerebral artery occlusion-reperfusion rats, MEM significantly prevented neuronal death by suppressing the activation of the calpain-caspase-3 pathway and apoptosis, consequently attenuating brain damage and neurological deficits [273]. In transient (60 min) ischemic stroke mice, low-dose MEM (0.2 mg/kg/day) started 24 h before stroke and continued for a 48-hour recovery period significantly reduced lesion volume by 30–50% and improved behavioral outcomes [275]. On the other hand, higher doses of MEM (20 mg/kg/day in this report) increased injury. The neuroprotective effect of MEM was also confirmed in stroke models using multiple species. In a rabbit multiple infarct embolic ischemia model, bolus injections of MEM at 25 mg/kg were lethal. However, slowly infused MEM was more tolerable and had substantial therapeutic benefits after acute ischemic stroke [276]. Like other NMDAR antagonists, delayed MEM treatments, e.g., 30–60 min after stroke, showed little neuroprotective effects [274, 276]. Unfortunately, pretreatment of MEM before stroke attacks is generally unpractical and hardly justifiable to apparently normal individuals.

In vivo studies verified that chronic oral MEM at clinical doses (1–30 mg/kg/day, for months to years) is well tolerated without significant neuronal or neurological abnormalities [20, 277–281]. Clinical trials with MEM have consistently demonstrated its safety for short- and long-term use, with an adverse event profile “similar to that of placebo” [282–286]. MEM may cause a few side effects that have been clinically well characterized; the most common adverse reactions include dizziness, headache, confusion, diarrhea, and constipation [287–289]. Other possible less common side effects include fatigue, pain, hypertension, weight gain, hallucination, confusion, aggression, vomiting, and urinary incontinence. These reactions are not life-threatening, and symptoms are treatable and reversible. However, as seen with most drug therapies, continual use of high doses of MEM (e.g., ≥ 30 mg/kg) may block synaptic and extrasynaptic NMDARs and can show side effects of neuronal loss and functional impairments [109].

**Fig. 6:**
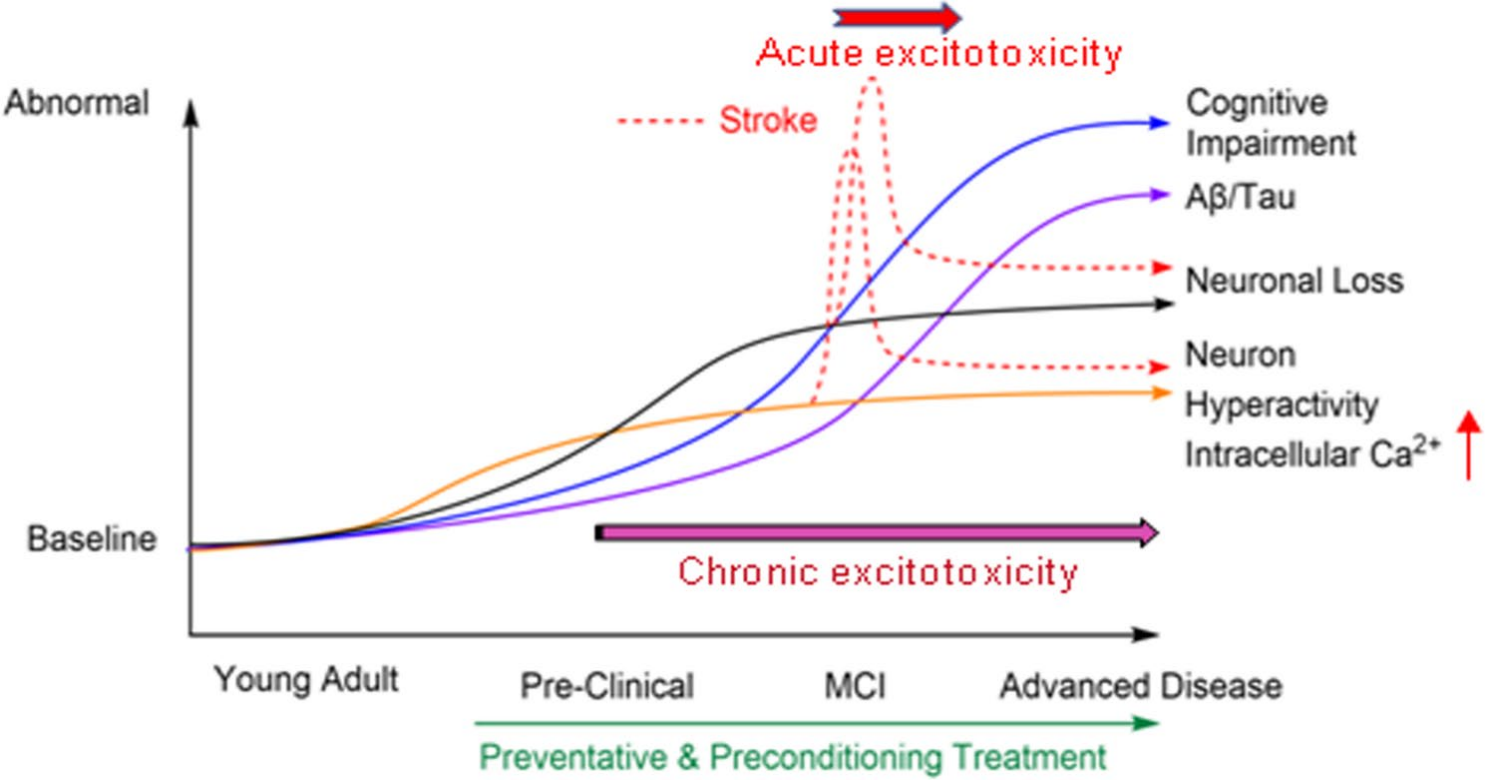
Hypothetic timelines of late-onset AD and common comorbidity of stroke. Late-onset AD is a slow and progressive disease; its early pathophysiological cascades cultivate years to decades before clinical diagnosis and likely precede significant Aβ deposition which is a pathological event emerging in patients’ brains of around 50 years old [48]. Different from the most popular diagram showing the events after Aβ deposition [356], this graph emphasizes possible triggering mechanisms before Aβ and tau pathology. In this hypothetic model, neuronal hyperactivity and Ca^2+^-associated chronic excitotoxicity exist well before neuronal loss, functional deficits, increased APP processing, and Aβ/tau pathology. Meanwhile, these underlying mechanisms significantly increase the risk of stroke attacks accompanied by acute excitotoxicity. Accordingly, a preventive disease-modifying intervention such as MEM treatment is necessary in the preclinical phase, which can also serve as a preconditioning therapy against stroke that strikes more than 50% of AD patients

MEM is thus far the only clinically approved NMDAR antagonist for the treatment of moderate-to-severe AD [282–286] (Table 1). The rationale for using MEM as a symptomatic treatment for advanced AD patients, but not as a disease-modifying early treatment, is in line with the previous judgment that NMDAR abnormalities are merely a consequence of Aβ/tau pathology. This justification, however, is noticeably inconsistent with mounting evidence from basic and clinical observations that NMDAR and neuronal hyperactivity are early and pathogenic mechanisms of AD development. According to the modified Ca^2+^ hypothesis of AD and recent evidence from our group and others that NMDAR overactivation and chronic Ca^2+^ dyshomeostasis are upstream events of AD pathology, including Aβ/tau alterations [127], it can be reasonably assumed that the marginal results of current MEM treatment in advanced AD patients are largely due to improper timing of the delayed treatment, which misses the pathogenic phase of neuronal hyperactivity ongoing for years during the prodromal/preclinical period of disease progression. A game-changing approach should be considered to start MEM treatment much earlier in individuals who show persistent signs of neuronal hyperactivities, Ca^2+^ dyshomeostasis, and other risk factors/early biomarkers of AD/ADRD (Fig. 6).

**Table 1:** Clinical trials of NMDAR antagonists showing positive and promising potentials for the treatment of ischemic stroke or AD/ADRD

Drug name	Primary disease target	Trial phase	Year of study or report	Participates	Finding or outcome measurement	Comments	References
***AD, MCI and Dementia Trials***							
Memantine (MEM)	Moderate and severe AD	Phase 3	2003	369	MEM showed significant cognition benefits in moderate and severe AD patients	Received FDA approval in 2003 as a symptomatic treatment for advanced AD	[355–358]
MEM	Mild to moderate Vascular dementia	Phase 3	2002	288–321	MEM 10 mg/day X 28 weeks improved cognition with no deterioration in global functioning and behavior.	A multicenter trial in France	[298]
MEM	Mild to moderate AD	?	2007	403	MEM 10 mg twice/day X 24 weeks improved language and some aspects of memory (ADAS-cog scores)	A post hoc analysis of a randomized, double-blind, placebo-controlled trial in France	[297]
MEM	Mild to moderate AD	?	2008	470	MEM 20 mg/day X 24 weeks was safe and tolerable; MEM significantly improved ADAS-cog and CIBIC-plus scores at weeks 12 and 18, and numerical superiority at week 24 on both scales.	A double-blind, placebo-controlled trial in Europe	[300]
MEM	Moderate AD	Pilot trial	2007	36	MEM 20 mg/day X 52 weeks showed less decline in glucose metabolism in all brain areas than patients on placebo. The loss of hippocampal volume was substantially smaller (2.4% vs. 4.0%).	A randomized, double-blind, placebo-controlled pilot study	[294]
MEM	Mild to moderate AD	?	2006–2008	167	MEM 10 mg twice/day X 24 weeks reduced agitation as measured by the NPI compared to donepezil.	Post hoc analysis of a multi-sited, double-blind clinical trial in China	[296]
MEM	Mild cognitive impairment (MCI)	?	2017	75	MEM 5–20 mg/day X 48 weeks improved cognitive and other behavioral performance in multiple tests. Single-photon emission computed tomography (SPECT) scan showed correlations with functional performance.	The initial MEM dose was 5 mg once daily and increased weekly by 5 mg/day in divided doses to a total dosage of 20 mg/day.	[299]
MN-08 (Memantine nitrate derivative)	Healthy adults	Phase 1	2023	16	To assess the safety and tolerability of MN-08 for 6.5 consecutive days in healthy subjects	Ongoing	ClinialTrials.gov; ID: NCT05660863
MN-08 (Memantine nitrate derivative)	Alzheimer’s disease	Phase 2	2023	?	Potential functional benefits of improving cognition	Ongoing	[359]
Lithium	MCI	Phase 2	2007	80	Long-term (2-year) lithium attenuated cognitive and functional decline in amnestic MCI, and modified AD-related CSF biomarkers; well tolerable and showed no impairment to renal function	See published reports for more information	• [360–362]
Lithium	MCI	Phase 4	2017–2024	80	To delineate whether lithium has anti-dementia properties in older adults who have mild cognitive impairment and are at risk of becoming demented.	Ongoing	ClinicalTrials.gov ID:NCT03185208
***Stroke Trials***							
MEM	Chronic poststroke aphasia	Phase 3	2009	28	Significantly short and long-term functional improvements and even greater effects combined with constraint-induced aphasia therapy (CIAT).	See published report for more information	[363]
MEM	Stroke	Phase 1	2014	20	Safety, drop-out rates, feasibility, and establishment of effect sizes	No publication identified	Clinicaltrials.gov; ID: NCT02144584
MEM	Mild to moderate cerebral thromboembolic stroke	Phase 3	2014	53	Combined with standard treatment resulted in a remarkably improved neurological function.	See published report for more information	[364]
MEM	Intracerebral Hemorrhage (ICH) stroke	?	2015	64	Early administration of memantine to ICH patients resulted significant improvement of long-term motor function and functional independence.	See published report for more information	[365]
MEM	Ischemic stroke	Phase 3	2015	47	Functional outcomes including NIHSS and mRS 7–21 days after ischemic stroke	No publication identified	ClinicalTrials.gov; ID: NCT02535611
MEM	Ischemic stroke	?	2021	77	Reduction of serum MMP-9; significantly improved NIHSS and BI during and after inpatient hospital care	See published report for more information	[366]
NEU2000 GluN2B selective NMDAR antagonist	Ischemic stroke; for patients subjected to endovascular thrombectomy less than 8 h from symptom onset	Phase 2	2018	?	The trial is to examine the safety and efficacy on tissue damage and clinical outcomes	Multicenter, randomized, double-blinded, placebo-controlled trial (high and low doses)	[367]; The report describes the trial protocol, no trial outcome reported yet.
Ketamine	Ischemic stroke	Phase 1 and 2	2015–2018	50	To determine neuroprotection of ketamine combined with tPA in acute ischemic stroke	No publication identified	ClinicalTrials.gov; ID: NCT02258204
Ketamine	Subarachnoid Hemorrhage	Phase 2 and 3	2016–2021	50	To test the efficacy of ketamine in protecting the brain following aneurysm repair operation	No publication identified	ClinicalTrials.gov; ID: NCT02636218
Ketamine	Ischemic stroke/depression	Phase 1	2020–2022	21	To assess the effect and safety of transmucosal ketamine in treatment of post-stroke depression	No publication identified	ClinicalTrials.gov; ID: NCT04876066
Ketamine	Ischemic stroke	Phase 2 and 3	2023–2025	120	To assess the efficacy and side effects of intravenous ketamine against excitotoxicity	No publication identified	ClinicalTrials.gov; ID: NCT03223220
Lithium	Stroke risk in bipolar patients	Retrograde Meta-analysis	2001–2011	1,250	Chronic lithium treatments significantly reduced risk of stroke in patients with bipolar disorder.	Completed	[368, 369]

Many MCI/AD clinical trials tested combined therapies of MEM plus a second treatment; these trials are not listed here because of their different focus. In stroke trials, MEM was administered minutes to hours after the onset of stroke symptoms [370, 371]. No MEM preconditioning was tested. The question mark in the Table indicates a lack of or unclear information about the phase or subject number of the trial. Lithium is a broad-spectrum drug with an anti-NMDAR activity. Due to its multifaceted actions, lithium is not the focus of this Table. In most trials, mild-to-moderate AD was defined by the Mini-Mental State Examination (MMSE) score of 10–20/22

There have been over hundreds of clinical trials with NMDAR antagonists for the treatment of stroke and AD, respectively, most trials failed to show functional benefits in patients due to a variety of issues and dilemmas. This table was generated to briefly summarize promising NMDAR antagonists, focusing on recent trials of memantine and ketamine rather than providing a comprehensive list of trials. There has been no clinical trial of dual therapy against the comorbidity of stroke and AD/ADRD or MCI.

### Early MEM treatment as a disease-modifying therapy for AD and related dementia

MEM treatments at early phase of incubation period is essential for a disease-modifying or preventive therapy in order to maintain year-long normal NMDAR activity and physiological Ca^2+^ levels in individuals vulnerable to AD/ADRD. In the GluN3A KO mouse, we demonstrated that presymptomatic MEM (10–20 mg/day) treatment from 3-month of age for 3 months prevented or attenuated AD brain neuropathology, Aβ production/aggregation, and cognation decline [127, 290]. Audrain et al. examined preventive treatment in a late-onset AD rat model. The early MEM (20 mg/daily) administration for 6 months started at asymptomatic phase of 4 months old promoted non-amyloidogenic cleavage of APP followed by a decrease in soluble Aβ42. MEM also prevented impairments of LTP and cognitive decline in control AD rats, although tau hyperphosphorylation was unaffected [291].

Thanks to the identification of risk factors and associated mutant genes in human genome research [292–295], early and presymptomatic treatments become possible. Supporting this idea, clinical trials of MEM in mild cognitive impairment (MCI)/mild AD patients showed significant therapeutic benefits, such as maintained cognitive function and improved brain imaging findings [296–302] (Table 1); even so, MEM in these trials might not have been given early enough from presymptomatic phases. In fact, MEM has been frequently prescribed to MCI and mild AD/dementia patients based on many physicians’ own clinical experience [303]. Despite the emerging evidence, a meta-analysis review of data involving early MEM approaches concluded that early MEM treatments did not provide significant benefits for patients in trials analyzed. However, the authors also recognized that “Prospective trials are needed to further assess the potential for efficacy of memantine” [304]. Specifically, considering many failures of Aβ clearing therapies in clinical trials and documented benefits of MEM in MCI/AD patients, it is necessary to verify the effect of early preventive treatment using MEM and other eNMDAR antagonists in both preclinical and clinical investigations [298–302] (Fig. 6) (Table 1).

Ketamine is a non-competitive NMDAR antagonist, initially developed as an anesthetic drug. Besides multimodal analgesic actions, ketamine can induce a wide range of pharmacological effects, including neuroprotection, anti-inflammatory, anti-cancer, anti-depression/suicidal attempts, and status epilepticus [305–307]. In dose-dependent and brain status-dependent manners, ketamine displays neuroprotective or neurotoxic properties. At anesthetic doses applied during neurodevelopment, ketamine contributes to inflammation, autophagy, apoptosis, and enhances levels of reactive oxygen species [308]. On the other hand, a subanesthetic dose ketamine triggers multiple neurotrophic and neuroprotective effects mediated by NMDAR-dependent and -independent mechanisms. Regarding its anti-depression action, recent studies explored using ketamine to treat AD-related depression [309–311]. Esketamine, which is ketamine formulated as a nasal spray, was approved by the FDA as an adjuvant drug to be used for treatment-resistant depression (TRD) [312].

Being an NMDAR antagonist, ketamine has been tested in several stroke trials for safety and neuroprotective efficacy while no official reports are available up to now (Table 1). Despite potential ketamine’s cognitive effects, few clinical trials have examined its cognition benefits in AD patients. There has been no clinical trial to test ketamine as an anti-AD drug, mostly likely due to the current focus on the Aβ mechanism and concerns about possible side effects caused by prolonged use of ketamine.

### Early preventive neuroprotective treatments for AD and stroke in the same individuals

Although multiple factors and shortcomings in preclinical and clinical research may contribute to the failure of NMDAR antagonists in stroke trials, one critical dilemma is the narrow therapeutic window. An NMDA receptor antagonist, even if it has few side effects, must be administered before or soon after (within a couple/few hours) the onset of ischemic attack to show protective effects in animal stroke models, which is generally impractical in clinical settings [313]. Aside from acute neuroprotective treatment, mounting evidence demonstrated that the brain and neuronal cells can be preconditioned using sublethal ischemia/hypoxia or a variety of chemicals/drugs to substantially enhance the tolerance to severe upcoming ischemic insults. This preconditioning strategy and its potent and broad cytoprotection have been confirmed in different animal models and human studies [314–316]. Like NMDAR antagonists, preconditioning treatment faces the same hurdle in that it requires pre-application well before the onset of an ischemic insult, and it is unfeasible to predict when somebody, even if he or she is known to be susceptible to stroke, will experience an ischemic attack in daily life.

Considering that stroke and AD share some key pathophysiological mechanisms with different time courses, a continual prophylactic pretreatment targeting common underlying NMDAR-related mechanisms at the early stages of AD is expected to be preventive for both stroke and AD, i.e., slowing AD progression while simultaneously priming the same brain against ischemic attacks that might strike at any time in the same aging individual (Fig. 6). There has been no such ideal treatment targeting both stroke and AD because it has been believed for a long time that the pathogenesis of AD is solely due to Aβ and tau pathology, which is fundamentally different from the cerebral ischemia that causes acute brain neurovascular damage.

To this end, we have performed the first investigation to test the preventive anti-stroke and anti-AD effects of MEM in the novel sporadic AD model of GluN3A KO mice as well as conventional 5XFAD mice [317]. Memantine (10 mg/kg/day in drinking water) was administered during the prodromal/preclinical stage to 3-month-old mice when olfactory deficits and neuronal hyperactivity were detectable but no cognitive dysfunction was present. After 3 months of treatment, significant benefits were observed in mice in the MEM group, showing a slowing of AD neuropathology and functional deterioration. Focal ischemic stroke was then induced in AD mice with and without MEM treatments to mimic the strokes commonly occurring in over 50% of AD patients. Compared to the vehicle group, the infarct volume and neuronal loss in AD mice that received 3 months of MEM treatment were significantly reduced 3 days after stroke. Continual monitoring and inspection of these animals 3 months later revealed less neurodegeneration and fewer cognitive deficits in the AD-stroke mice that received long-term MEM treatments. This ongoing investigation provides the first supporting evidence that early MEM treatment can be a preventive/preconditioning therapy for aging individuals susceptible to stroke and AD. Based on previous and our investigations, this innovative early approach is mechanistically justified, clinically feasible, and bears great clinical significance. More basic and preclinical research studies will help to reveal the detailed mechanisms of the dual effects of MEM against acute and chronic excitotoxicity. Clinical trials using MEM as a preventive therapy at the prodromal and mild MCI/AD stages should be carried out, and the effect of MEM on the prevalence and severity of stroke in AD patients should be explicitly analyzed and compared with that in patients not taking MEM. Meanwhile, the interaction between NMDARs and TRPMs as a downstream pathway to excitotoxicity provides another target for producing effective therapies against stroke and AD/ADRD. A better understanding of its physiological functions will help to predict the safety and efficacy of this approach.

### Development of selective eNMDAR antagonists for enhanced therapeutic benefits

There has been increasing enthusiasm for the development of eNMDAR antagonists as potential treatments for stroke or neurodegenerative diseases such as AD and HD [107, 108]. Most of these compounds were MEM derivatives, such as MN-08 (a MEM nitrate) [153, 268, 318], fluoroethylnormemantine (FENM) [319, 320], and NitroSynapsin [153]. In vitro experiments with MN-08 demonstrated its anti-NMDAR effect and reduced Ca^2+^ influx, regulation of the ERK and PI3K/Akt/GSK3β pathways, and attenuation of glutamate-induced neuronal loss. In APP/PS1 transgenic mice and 3xTG-AD mice, several months of MN-08 daily treatments attenuated Aβ accumulation, neuronal and dendritic spine loss, and cognitive deficits. In addition, MN-08 had favorable pharmacokinetics, blood-brain barrier penetration, and safety profiles in rats and beagle dogs. These findings suggest that the novel memantine nitrate MN-08 may be a useful therapeutic agent for AD [321]. To improve the therapeutic potential and benefits of MEM, the Lipton group generated a series of drugs known as NitroMemantines, including the derivative of MEM-designated NitroSynapsin [153, 268, 318]. NitroSynapsin is a chemical adduct between an aminoadamantane moiety and a nitro group. Unlike MEM, NitroSynapsin acts as a dual-allosteric antagonist of eNMDARs, with aminoadamantane serving to target the nitro group to redox-modulatory/inhibitory sites on the extracellular surface of the receptor via S-nitrosylation. The pharmacological and therapeutic properties of NitroSynapsin have been examined and compared with those of MEM through in vitro and in vivo experiments. Patch clamp single-channel recordings confirmed that, like MEM, NitroSynapsin is a selective eNMDAR antagonist and can antagonize α-synuclein-induced synaptic damage and neuronal loss [322]. Human iPS cells (hiPSCs) and organoids bearing familial AD mutations exhibit aberrant electrical activity manifested as increased spontaneous action potentials, slow oscillatory events, and hypersynchronous network activity. NitroSynapsin, but not MEM, abrogated this hyperactivity [323].

To improve the specificity of the action of MEM on eNMDARs, a bioengineering approach was taken to design a hybrid nano-compound (AuM) with MEM attached via polymer linkers to a gold nanoparticle, the size of which is larger than the synaptic cleft [324]. AuM efficiently and selectively inhibited eNMDARs without inhibiting sNMDARs, and in comparison to MEM, AuM exhibited superior neuroprotective properties against NMDA-induced excitotoxicity and Aβ oligomer-induced dendritic spine loss [324]. This interesting drug design may represent a novel rational strategy for a new class of neuroprotective drugs with enhanced selectivity for eNMDARs that are effective in the treatment of stroke and neurodegenerative diseases.

Neramexane, a noncompetitive moderate open channel NMDAR antagonist as well as an inhibitor of cholinergic nicotinic receptors, has been shown to be efficient in enhancing long-term memory in adult rats and well tolerated in humans, suggesting potential therapeutic applications [325, 326]. In a clinical trial of the treatment of tinnitus, four weeks of 50 mg/day neramexane significantly improved functional scores compared to placebo [327]. Some early phase II/III clinical trials with neramexane for moderate-to-severe AD, however, showed contradictory results [326], which may be a reflection of the broader effects of neramexane and the timing of the drug administration, as discussed in this review.

Ifenprodil, a specific GluN2B receptor antagonist, prevents Aβ-induced endoplasmic reticulum (ER) stress, hippocampal dysfunction, and microtubule deregulation as well as Ca^2+^ rise [233]. Ifenprodil also prevents Aβ-induced inhibition of LTP, impairment of synaptic transmission, and retraction of synaptic contacts [328]. In acute hippocampal slices, the selective GluN2B antagonists ifenprodil and Ro25-6981 efficiently rescued LTP inhibition caused by soluble Aβ [328]. These results suggest that targeting the GluN2B subunit of NMDARs may be a promising way to prevent AD progression.

The compound 4-(5-(4-bromophenyl)-3-(6-methyl-2-oxo-4-phenyl-1,2-dihydroquinolin-3-yl)-4,5-dihydro-1 H-pyrazol-1-yl)-4-oxobutanoic acid (DQP-1105) is a representative member of a new class of NMDAR antagonists and shows a preferred effect on GluN2C/2D subunits [329]. DQP-1105 was more potent for blocking currents evoked by bath-applied NMDA than for blocking synaptic NMDA currents. Thus, DQP-1105, like MEM, seems to have the potential to provide efficacy in therapeutic treatment while displaying minimal side effects.

With high clinical feasibility, the clinical drug lithium, which has been used for treating bipolar disease and depression, has drawn increasing attention for its multifaceted neuroprotective and regenerative mechanisms in the treatment of neurodegenerative diseases [330, 331]. Among its effects on cellular and molecular signaling pathways, lithium has been shown to reduce free radical-induced neurotoxicity and stabilize aberrant Ca^2+^ dyshomeostasis by an inhibitory action at NMDARs [332–334]. Lithium prevents intracellular Ca^2+^ overload by suppressing IP3R-mediated ER Ca^2+^ release, subsequently attenuating Aβ accumulation and tau hyperphosphorylation and rescuing impaired hippocampal synaptic plasticity [335, 336]. Lithium is also a GSK3β inhibitor [337], which can be an underlying mechanism to attenuate Aβ-induced tau phosphorylation and toxicity [253]. Lithium is a tolerable drug, and its anti-excitotoxicity and anti-AD properties merit further investigation.

## Conclusion

Excitatory hyperactivity associated with the imbalance of the excitatory/inhibitory activity and overactivation of NMDARs, especially eNMDARs, and increased [Ca^2+^]_i_ cause the acute and chronic excitotoxicity of brain injuries such as ischemic stroke and neurodegenerative diseases such as AD [185, 338]. These decisive roles of NMDARs position them as major molecular and cellular players in critical brain functions and pertinent potential therapeutic targets for neurological disorders [61]. However, the cause of degenerative excitotoxicity and distinctions between acute and chronic forms of excitotoxicity has not been explicitly defined. Retrograde assessments regarding stroke prevalence and outcomes in MCI/AD/ADRD patients who were and were not prescribed chronic MEM treatments may provide indicative evidence for the dual efficacy of MEM; this clinical analysis, however, has so far not been performed.

A growing consensus considers that Aβ pathology is unlikely the initial pathogenic mechanism, at least not the only pathogenesis, for late-onset AD/ADRD. A better understanding of the causal mechanism of Aβ deposition, glutamatergic hyperactivity and downstream cascades, either in Aβ-dependent or -independent manners, will shine a new light onto the initial pathogenesis and aid in the development of early, preventive/preconditioning drug therapies with dual effects against late-onset AD/ADRD and ischemic stroke that often occur in the same individuals.

## Electronic supplementary material

Below is the link to the electronic supplementary material.

Supplementary Material 1

Supplementary Material 2

## Data Availability

All data and material reviewed in this article are available from the PubMed online search.

## List of abbreviations

AD: Alzheimer’s disease
AMPA: α-amino-3-hydroxy-5-methyl-4-isoxazolepropionic acid
APP: amyloid precursor protein
ARIA: amyloid-related imaging abnormality
ARIA-E: amyloid-related imaging abnormalities (ARIAs)-cerebral edema
ARIA-H: ARIA-related microhemorrhages
Aβ: β-amyloid
BDNF: brain-derived neurotrophic factor
[Ca^2+^]_i_: intracellular free Ca^2+^
CaMKII: Ca^2+^-calmodulin kinase II
CaMKIV: Ca^2+^-calmodulin kinase IV
CBP: CREB-binding protein
CREB: Cyclic-AMP response element binding protein
CNS: central nervous system
DQP-1105: 4-(5-(4-bromophenyl)-3-(6-methyl-2-oxo-4-phenyl-1,2-dihydroquinolin-3-yl)-4,5-dihydro-1 H-pyrazol-1-yl)-4-oxobutanoic acid
EAATs: excitatory amino acid transporters
eNMDARs: extrasynaptic NMDARs
FDA: Federal Drug Administration
GSK-3β: glycogen synthase kinase 3β
FAD: familial AD
FENM: fluoroethylnormemantine
HD: Huntington’s disease
hiPSCs: human iPS cells
KO: knockout
LTD: long-term depression
LTP: long-term potentiation
MEM: memantine
MCI: mild cognitive impairment
NMDARs: N-methyl-D-aspartate receptors
PCP: phencyclidine
PSD: postsynaptic density
PSD: post-stroke dementia
rTPA: recombinant tissue plasminogen activator
ROS: reactive oxygen species
sNMDAR: synaptic NMDARs
STEP: striatal-enriched protein tyrosine phosphatase
TRD: treatment-resistant depression
TRPM: transient receptor potential melastatin
TTX: tetrodotoxin
vGlut: vesicular glutamate transporter
