# Supplementary material for: Extrasynaptic NMDA receptors in acute and chronic excitotoxicity: implications for preventive treatments of ischemic stroke and late-onset Alzheimer’s disease
Source: Mol Neurodegener. 2023 Jul 3;18:43. doi: 10.1186/s13024-023-00636-1 (PMC10318843; doi:10.1186/s13024-023-00636-1)
Supplement: Supplementary file 1 — Supplementary Material 1 [file 13024_2023_636_MOESM1_ESM.docx]

**Extrasynaptic NMDA Receptors in Acute and Chronic Excitotoxicity:**

**Implications for Preventive Treatments Against Comorbidity of Ischemic Stroke and Late-onset Alzheimer’s Disease**

Shan P. Yu*^1,2^, Michael Q. Jiang^1,2^, Seong S. Shim^2^, Soheila Pourkhodadad^1,2^, and Ling Wei*^1^

^1^Department of Anesthesiology, Emory University School of Medicine, Atlanta, GA 30322, USA

^2^Center for Visual & Neurocognitive Rehabilitation, Atlanta VA Medical Center, Decatur, GA 30033, USA

* Corresponding authors

Shan Ping Yu

Ling Wei

101 Woodruff Circle

Woodruff Memorial Research Building

Suite 617A and 617B

Emory University School of Medicine

Atlanta, GA 30322

Email: [spyu@emory.edu](mailto:spyu@emory.edu) and [lwei7@emory.edu](mailto:lwei7@emory.edu)

**Abstract**

Stroke and late-onset Alzheimer’s disease (AD) are risk factors for each other; the comorbidity of these brain disorders in aging individuals represents a significant challenge in basic research and clinical practice. The similarities and differences between stroke and AD in terms of pathogenesis and pathophysiology, however, have rarely been comparably reviewed. Here, we discuss the related research background and the recent progress that are important and informative for addressing the comorbidity of stroke and late-onset AD and related dementia (ADRD). Glutamatergic NMDA receptor (NMDAR) activity and NMDAR-mediated Ca^2+^ influx are essential for neuronal function and cell survival. An ischemic insult, however, can cause rapid increases in glutamate concentration and excessive activation of NMDARs, leading to swift Ca^2+^ overload in neuronal cells and acute excitotoxicity within hours and days. On the other hand, mild upregulation of NMDAR activity, commonly seen in AD animal models and patients, is not immediately cytotoxic. Sustained NMDAR hyperactivity and Ca^2+^ dysregulation lasting from months to years, nevertheless, can be pathogenic for slowly evolved excitotoxicity in the development of AD/ADRD. Specifically, Ca^2+^ influx mediated by extrasynaptic NMDARs (eNMDARs) and a downstream pathway mediated by transient receptor potential cation channel subfamily M member (TRPM) are primarily responsible for excitotoxicity. On the other hand, the NMDAR subunit GluN3A plays a “gatekeeper” role in NMDAR activity and a neuroprotective role against both acute and chronic excitotoxicity. Thus, ischemic stroke and AD share an NMDAR- and Ca^2+^-mediated pathogenic mechanism that provides a common receptor target for preventive and possibly disease-modifying therapies. Memantine (MEM) preferentially blocks eNMDARs and was approved by the Federal Drug Administration (FDA) for symptomatic treatment of moderate-to-severe AD with variable efficacy. According to the pathogenic role of eNMDARs, it is conceivable that MEM and other eNMDAR antagonists should be administered much earlier, preferably during the presymptomatic phases of AD/ADRD. This anti-AD treatment could simultaneously serve as a preconditioning strategy against stroke that attacks ≥50% of AD patients. Future research on the regulation of NMDARs, enduring control of eNMDARs, Ca^2+^ homeostasis, and downstream events will provide a promising opportunity to understand and treat the comorbidity of AD/ADRD and stroke.

**Keywords:** Glutamate, NMDA receptors, excitotoxicity, GluN2B subunit, GluN3A subunit, extrasynaptic NMDARs, Ca^2+^ homeostasis, ischemic stroke, Alzheimer’s disease, memantine

**Background**

Acute ischemic stroke is a leading cause of death and disability in senior populations [1, 2]. One-third of stroke patients develop post-stroke dementia within 3 years and show pathological alterations resembling those of Alzheimer’s disease (AD), including the development of delayed cell death, cognitive decline, and β-amyloid (Aβ) deposition in the brain [3, 4]. As progressive neurodegenerative diseases, late-onset AD and AD-related dementia (ADRD) account for over 60-80% of dementia cases in people over 65 [5, 6]. These patients have a high risk of stroke; over half of them experience at least one stroke attack and suffer more severe outcomes including increased mortality rates than non-AD patients [7]. A national longitudinal cohort study (2007–2017) compared 12,629 ischemic stroke patients with dementia to 57,954 matched stroke non-dementia controls. The study identified dementia before stroke as an independent predictor of death. Over time, mortality in patients with dementia remained increased [8]. Even though stroke and late-onset AD/ADRD are interrelated common comorbidities in the same aging/aged individuals [7, 9, 10], the pathophysiology associated with different time courses of disease development and their destructive impacts on each other are not explicitly understood. Historically and currently, stroke and AD have been investigated in separate research fields and are regarded as distinct acute and chronic brain disorders, respectively. Increasing evidence, however, has revealed that stroke and AD share many hallmark pathophysiological alterations, including overactivations of glutamatergic N-methyl-D-aspartate (NMDA) receptors (NMDARs), increases in intracellular free Ca^2+^ ([Ca^2+^]_i_), disruptions of energy metabolism, excitotoxicity-induced neuronal loss, programmed cell death, synaptic/neural network impairments, neurovascular damage, neuroinflammation, Aβ/tau deposition, and progressive psychological/cognitive decline [2, 11-15]. Up to now, there is no effective disease-modifying treatment for either stroke or AD patients. After decades of study in both research fields, many neuroprotective and anti-amyloid treatments have failed clinical translations, showing inconsistent or no functional benefits because of a variety of dilemmas and obstacles [16-18].

Currently, approved treatments for ischemic stroke are limited to recombinant tissue plasminogen activator (rTPA) and endovascular thrombectomy, both of which can be highly effective within narrow therapeutic windows (4.5 and 6 hours after stroke onset, respectively). Unfortunately, only a small fraction of stroke patients qualify clinically for these treatments [19]. For AD patients, cholinesterase inhibitors and the NMDAR low-affinity, uncompetitive antagonist memantine (MEM) are among the few drugs approved by FDA as symptomatic treatments for moderate-to-severe AD, albeit with variable efficacy [20]. Aducanumab is a human monoclonal antibody directed against aggregated soluble and insoluble forms of Aβ. Two Phase III clinical trials (EMERGE and ENGAGE) in mild cognitive impairment (MCI) and mild AD patients ended with conflicting results: EMERGE of high dose aducanumab slowed cognitive decline, but ENGAGE observed no clinical benefits and the trial had to be terminated early [21, 22]. Noticeably, among patients treated with high-dose aducanumab, ∼35% of them experienced amyloid-related imaging abnormalities (ARIAs), such as ARIA-related cerebral edema (ARIA-E), and a further ∼20% had ARIA-related microhemorrhages (ARIA-H), among other side effects. Even higher rates (43-65%) of ARIAs were observed in ApoE ε4 carriers [23]. These results were consistent with other clinical studies using anti-Aβ therapies [21, 24, 25]. Moreover, clinical trials with aducanumab and similar compounds have been carried out via FDA “accelerated approval”, their clinical efficacy and risks remain to be further validated in peer-reviewed reports and clinical practice. The repeated failures and disruptive side-effects of anti-Aβ treatments based on the mechanism of familial AD (FAD) call for alternative strategies with innovative and out of the box thinking on the pathogenesis of late-onset AD in order to develop clinically effective treatments for most AD cases.

Compelling findings from clinical analyzes reveal that ischemic stroke and AD/ADRD are significant risk factors for each other [8, 26-28]. A better understanding of the relationship and interactions between these two neurological diseases of the central nervous system (CNS) should help the development of treatments that target the shared mechanisms and show efficacy for both disorders in susceptible individuals. As a lesson learned from previous successes and failures, such clinical therapies can only be developed through unbiased, fact-based, and disease-specific mechanism-driven approaches consistent with clinical observations but not by digging further with conventional hypotheses inconsistent with clinical cases. If successful, multidisciplinary research will provide a breakthrough opportunity for the treatment of two neurological disorders that affect millions of people in the US and around the globe.

***Challenges in stroke and AD research and therapy development***

There have been several groundbreaking discoveries from decades of neuroscience research that have encouraged cautious optimism for therapy developments of neurological disorders such as stroke and AD. Significant advances may include the identification of glutamate excitotoxicity mediated primarily by overactivation of NMDARs and a downstream TRPM-mediated pathway, of distinct roles of the synaptic and extrasynaptic NMDARs (sNMDARs and eNMDARs, respectively) in synaptic plasticity and neuronal excitotoxicity, and of robust neuroprotection achievable by using NMDAR antagonists as well as endogenous protective mechanisms elicitable by preconditioning strategies against brain injuries. Unfortunately, none of these advances in basic and preclinical research has been successfully translated into clinical therapies, with MEM as an exception of limited success for advanced AD patients.

The failure of NMDAR antagonists in stroke clinical trials may be attributed to noticeable side effects, narrow therapeutic windows, the lack of vascular protection to restore local blood supply, and the complexity of cellular and molecular injury mechanisms in the human brain [16, 29, 30]. Despite these hurdles in the development of therapies for stroke, continual research confirms NMDAR-mediated excitotoxicity as the primary cell death mechanism [31], and the development of novel and safe NMDAR antagonists remains a top priority in stroke research. Consistent evidence suggests that the undesirable side effects of many NMDAR antagonists are possibly due to the paradoxical actions at synaptic and extrasynaptic NMDARs [32]. Therefore, in addition to the desire of targeting downstream pathways, more selective eNMDAR antagonists have become preferred choices to minimize side effects while enhancing the neuroprotective efficacy.

In basic and clinical AD research, the amyloid hypothesis has been challenged by compelling observations of Aβ-independent pathogenesis, pathophysiology, and pathology in animal models and human patients [33-36]. Commonly used transgenic mouse models generated using FAD genes such as a forced expression of mutant amyloid precursor protein (APP) and/or Aβ genes do not accurately mimic late-onset sporadic AD in multiple aspects, including the trigger, origin, and time course of Aβ production as well as the lack of neuronal loss and tau pathology in some widely used FAD mice [37-39]. In some models of overexpressing APP, widespread Aβ deposition occurs with no subsequent cognitive deficits [40]. In a human APP (hAPP) transgenic mouse model of young and old ages (2-24 months of age), there was no evidence of amyloid deposits or neurodegeneration, even though the synaptic disruption was evident [41]. More significantly, while the Aβ cascade pathology has been the diagnostic standard of AD and changes in Aβ deposition/plaques or soluble Aβ have been shown during AD development, many healthy individuals may have significant Aβ plaques and tau tangles in the brain but develop no signs of cognitive deficits [34, 42-44]. Some studies identified that at least 20-30% of healthy aging individuals showed substantial amyloid deposits in the brain but never developed dementia in their lifetimes [45]. Furthermore, clinical trials of anti-Aβ therapies that can successfully remove amyloid plaques have resulted in few functional improvements [33, 34, 45, 46].

It is now recognized that AD pathophysiology begins many years prior to clinical diagnosis, with various degrees of severity and different time courses of progression [47]. It is recognized that the onset age of Aβ deposition in the human brain is approximately 50 years old [48]. During the aging process before and around this age, the root mechanism triggering abnormal Aβ production in late-onset AD has been unclear except for propositions of genetic influence [48] and vague concepts such as “cognitive reserve” [49]. Aside from extensively delineated mechanisms of amyloid metabolism, there has been little information on the initial trigger(s) and year/decade-long process of endogenous amyloid pathology. Collectively, these inconsistencies and the lack of an endogenous association of Aβ pathology in disease progression suggest that alternative or additional mechanisms may be responsible for neuronal damage and the development of sporadic AD/ADRD [33-36].

As a significant paradigm shift in the understanding of AD progressive pathophysiology, modulations of brain hyperexcitability and the balance of excitatory/inhibitory activities are an emerging research area based on the Ca^2+^ hypothesis in AD pathogenesis [50-54]. For example, the anticonvulsant drug levetiracetam, which modulates glutamate release and neuronal excitatory/inhibitory balance, has been explored as a disease-modifying therapy for AD and has advanced to clinical trials [51, 53, 55, 56]. More evidence suggests that chronic attenuation of neuronal hyperactivity leads to reduced APP/Aβ accumulation, implying that neuronal hyperactivity can be an upstream event in the development of amyloid pathology [57]. However, the causal mechanism of slowly evolved degenerative excitotoxicity and distinctions between acute and chronic forms of excitotoxicity have not been explicitly defined. A better understanding of the causal mechanisms of glutamatergic hyperactivity, especially in both Aβ-dependent and Aβ-independent manners, may shed new light on the root pathogenesis and aid in the development of early treatments for late-onset sporadic AD and ADRD.

***Ionotropic glutamatergic NMDA receptors and subunits***

Glutamate is the primary excitatory neurotransmitter in the CNS, and ionotropic glutamate receptors are responsible for neuronal communications crossing excitatory synapses. There are three subfamilies of ionotropic glutamate receptors: α-amino-3-hydroxy-5-methyl-4-isoxazolepropionic acid (AMPA) receptors, kainate receptors, and NMDARs [58-60]. Of these subtypes, NMDARs play major roles in Ca^2+^ homeostasis, neuronal excitability, synaptic plasticity, and excitotoxicity in neurophysiology and neuropathophysiology [61, 62]. NMDARs consist of GluN1 (NR1) subunits, GluN2 (NR2) subunits (GluN2A-2D), and a pair of GluN3 (NR3) subunits (GluN3A and GluN3B) [63, 64].

Although functional NMDARs can be formed by heterotetramers of two glycine/d-serine-binding GluN1 subunits paired with two glutamate-binding GluN2 subunits [65, 66] (Fig. 1), more recent research established that the majority of native NMDARs are triheteromers composed of two GluN1 and two unique GluN2 or a combination of GluN2 and GluN3 subunits [67, 68]. In contrast to diheteromeric structures, triheteromeric NMDARs display an intermediate sensitivity to glycine and glutamate due to either differences in channel ion conductance, open/close kinetics, or both as a result from the presence of various GluN2/3 subunits [67, 68]. Specifically, GluN3 subunits are differentiated by a positive charge in the pore-lining sequence that confers a unique structural alteration to receptors. NMDARs containing the GluN3 subunit are triheteromers composed of GluN1, GluN2, and GluN3 subunits. These receptors exhibit reduced Ca^2+^ permeability compared to GluN1/GluN2 receptors and attenuated Mg^2+^-block at hyperpolarized membrane potentials. Thus, the GluN3 subunit plays an unique role as a dominant-negative modulator in triheteromeric NMDARs [69-72]. In this event, GluN3 subunits may facilitate NMDA receptor activation due to their reduced magnesium sensitivity and reduce their conductance relative to GluN2A or GluN2B subunits. GluN1 and GluN3 can form diheteromeric receptors that are activated by glycine but not by glutamate [73]. Thus, strictly speaking, the GluN1/GluN3 complex is a glycine receptor, but essentially no longer a glutamate receptor.

NMDARs have been identified in non-neuronal cells, including astrocytes, oligodendrocytes, polydendrocytes (i.e. NG2 glial cells), and blood lymphocytes [74-76]. Since glial cells play important physiological and pathophysiological roles in the CNS, NMDAR subunits in these cells may exhibit special characteristics different from those of neurons. Our previous studies revealed GluN3A containing NMDARs present in conducting cells of the kidney, which may regulate urinary concentrating capacity and play a protective role under ischemic/hypoxic conditions [77, 78]. The physiological and pathological roles of NMDARs in non-neuronal cells are not well understood and require further investigations.

***Functional roles of NMDARs and subunits at synaptic and extrasynaptic sites***

NMDARs are located not only at synapses but also at extrasynaptic sites [79, 80] (Fig. 2). Synaptic NMDARs are enriched with GluN2A, while eNMDARs are more likely to contain GluN2B, GluN3A, GluN3B, or GluN2C/2D subunits [81, 82]. GluN3A and 3B are mostly associated with the perisynaptic site of the postsynaptic density (PSD) [83]. There have also been reports of pre-synaptic localizations of GluN1, GluN2, and GluN3B [84]. The control of NMDAR activity and Ca^2+^ influx is critical for the induction of long-term potentiation (LTP) and long-term depression (LTD), which are believed to be closely associated with synaptic plasticity and learning/memory functions [85-87]. Early studies suggested that synaptic GluN2A-containing NMDARs and extrasynaptic GluN2B-containing NMDARs are differentially linked to the generation of LTP and LTD, respectively [88]. More recent evidence implicates that extrasynaptic NMDARs act as regulators of both LTP and LTD [86, 89, 90]. There is also some evidence to support that GluN2C/2D containing eNMDARs can regulate synaptic currents and interneuronal/intrinsic excitability [91-95].  For example, eNMDARs can regulate neuronal and neural network activity in striatal neurons [96]. The current view of the contribution of NMDARs to brain physiology and pathology does not solely rely on a dichotomy between GluN2A- and GluN2B-containing NMDARs, or between synaptic and extrasynaptic NMDARs. Under certain conditions, both sNMDAR and eNMDAR may influence different aspects of synaptic plasticity [97, 98].

In neuronal cultures, a comparable [Ca^2+^]_i_ increase induced by the activation of eNMDARs, but not by sNMDAR activation, leads to mitochondrial dysfunction and cell death [79, 99, 100]. Available evidence indicates that eNMDARs containing GluN2B are primarily related to excitotoxicity in stroke and neurogenerative diseases [99, 101, 102]. A few studies showed that GluN2D, likely in eNMDARs, contributes to excitotoxicity in retinal injury [103]. On the other hand, GluN2A-containing sNMDARs may play a role in excitotoxic cell death under certain experimental conditions [104-108]. For example, in hippocampal slices, the excitotoxicity induced by 50 µM NMDA for 30 min was attributed to sNMDARs based on the co-agonist D-serine and other pharmacological tools [87]. The discrepancy may be explained by the proposition that the balance between sNMDAR and eNMDAR activities is important in determining whether stimulation by NMDA or glutamate is neurotoxic or not [109]. It is more likely that drastic overactivation of both sNMDARs and eNMDARs accompanied by massive Ca^2+^ influx occurs *in vivo* and is a typical trigger of acute excitotoxicity commonly seen after ischemic strokes [110, 111]. Intriguingly, it was shown that GluN2C expression increased in hippocampal slices in response to ischemia; knocking out GluN2C exacerbated neuronal death in the CA1 area of the hippocampus and reduced spatial working memory compared to wild-type mice. *In vitro* and *in vivo* examinations revealed that GluN2C-expressing hippocampal neurons showed marked resistance to NMDA-induced toxicity and reduced Ca^2+^ influx, which is consistent with the notion that GluN2C-containing NMDARs exhibit a low Ca^2+^ permeability [112].

 GluN3 is highly expressed in many subcellular compartments during early development and is particularly concentrated in PSD-associated perisynaptic and extrasynaptic locations [70, 113] (Fig. 2). This is supported by ultrastructural evidence that GluN3A is more abundant at perisynaptic and extrasynaptic sites in both juvenile and adult animals [113, 114]. This pattern of expression appears consistent with the function of GluN3 in constraining eNMDAR activity during early life to limit excessive Ca^2+^ influx, which is favorable for protecting the immature brain. The removal of GluN3A later on during CNS development relieves the restriction on local Ca^2+^ upregulation and facilitates synaptogenesis, including the modulation of experience-driven synapse refinements [115]. Thus, the downregulation of GluN3A-containing NMDARs provides a developmental switch for activity-dependent maturation and stabilization of selected synapses, which are essential steps in synaptogenesis and memory consolidation [115]. Compared to the level in the neonatal brain, the total expression level of GluN3 is significantly reduced after brain development. Nevertheless, abundant GluN3 expression is still readily detectable in the adult brains of rodents as well as humans [116-120], suggesting that GluN3 has important functional roles in adult physiology and pathophysiology. Being a unique subunit affecting NMDAR activities, GluN3A exhibits functional roles in synaptic as well as extrasynaptic activities [121, 122]. The topography of GluN3 localization and activity are critically important across the lifespan. We propose that GluN3 acts as a tireless gatekeeper to prevent overactivations of eNMDARs and excessive Ca^2+^ influx, which is vital in maintaining Ca^2+^ homeostasis, cell viability, and normal neuronal functions (Fig. 2).

Some reports suggest that sNMDAR activation and eNMDAR activation contribute equally to excitotoxicity [123]. Alternatively, it was proposed that the subunit composition of NMDARs, such as the expression of GluN2B, but not the cellular location, is a determining factor for their effect on neuronal fate [124]. An imbalance between synaptic and extrasynaptic NMDA receptor activity could be a pathogenic factor for neurodegenerative diseases such as AD and Huntington’s disease (HD) [12, 107]. The disrupted balance may result from a malfunction or deficiency of an NMDAR subunit due to genetic mutation, mislocalization, or trafficking deficits of the subunit [125]. Imbalances may also be created by dysregulated glutamate concentrations in the vicinity of NMDARs. In this case, eNMDARs are directly responsible for glutamate excitotoxicity and cell death [79, 99, 100, 123]. For instance, the increased extrasynaptic composition of GluN2B-containing NMDARs or a deficiency of extrasynaptic GluN3A subunit induces enlarged tonic NMDAR currents that are closely associated with chronic excitotoxicity [80, 126, 127].

***Regulation of glutamate concentration in the extrasynaptic space and excitotoxicity***

As the primary excitatory neurotransmitter in neuronal communications, the glutamate concentration in the synaptic cleft is highly dynamic and tightly controlled, with rapid rises and falls (in milliseconds) within the µM to mM range to convey neuronal activity [128-131]. Glutamate is not restricted to the synaptic cleft; it also exists in the extrasynaptic space due to the spillover from the presynaptic release and secretion of glutamate by adjacent astrocytes and microglia [132-135] (Fig. 2). According to early assessments, the basal concentration of extrasynaptic glutamate is sustained at low μM levels [131, 136-139]. This might be an overestimation of the resting concentration; improved measurements in acute hippocampus slices have assessed the concentration to be approximately 100 nM [140, 141].

Studies on mixed cell cultures of neurons and astrocytes show that the glutamate concentrations that can lead to acute cell death (in hours to a few days) are in the high µM range (EC_50_ = 205 µM) [142]. This range appears too high for the extrasynaptic glutamate to reach in a persistent manner. On the other hand, sustained glutamatergic hyperactivity and Ca^2+^-associated chronic excitotoxicity are clearly identified in neurodegenerative diseases, such as AD. At present, little is known about the relationship between the glutamate concentration threshold and the duration of elevated extrasynaptic glutamate for inducing the slowly developed degenerative excitotoxicity underlying the prolonged process of neurodegeneration.

The extrasynaptic glutamate level is sensitive to abnormal and pathological conditions. For example, in rodents, stressful stimuli such as body restraint, forced swimming, and hypoxic insults can selectively increase the extrasynaptic glutamate concentration to over 30 µM or higher [143-145]. In stroke and brain injuries, the reduced extracellular volume associated with brain edema elevates the extrasynaptic glutamate concentration as a contributing factor in excitatory neuronal damage [146]. Extrasynaptic glutamate is taken up by neurons and astrocytes mostly via excitatory amino acid transporters (EAATs) and is metabolized in astrocytes to glutamine [147]. Multiple alterations of cellular and subcellular activities may cause increases in extrasynaptic glutamate. Among them, aberrant burst firing of presynaptic inputs and reduced glutamate clearance due to lower EAAT1-3 activity at depolarized membrane potentials can be the primary causes of increased extrasynaptic glutamate [146]. Other sources of increased extrasynaptic glutamate may include reversed operation of glutamate transporters [146].

In AD pathophysiology, deficient glutamate uptake and recycling contribute to elevated levels of extrasynaptic glutamate [148, 149]. Studies in neuronal cell cultures demonstrated that various species of Aβ peptides caused increased glutamate availability via their deteriorating effects on glutamate transporters [150-152]. It was proposed that Aβ-induced excitotoxicity is mediated by increasing extracellular glutamate concentrations due to decreased glutamate uptake from the synaptic cleft, which is correlated with impaired function of EAAT2 in perisynaptic astrocytes. Glutamate release from glial cells such as astrocytes and microglia [153] and decreased recycling of glutamate [154] may also contribute to chronic excitotoxicity.

The role of interplay between astrocytes and neurons has been further strengthened through recent work in human and rodent transcriptome analysis indicating weakened metabolism coordination between these cells under neuropathological conditions. In particular, reactive glia were less evident in TREM2-R47H and TREM2-R62H carriers than in noncarriers, implicating TREM2 and glia-neuron interactions in both mouse and human AD [155, 156]. In AD patients, decreased expression and capacity of glutamate transporters, specifically a selective loss of vesicular glutamate transporter (vGlut), were detected [154, 157]. Moreover, EAAT2 located in perisynaptic astrocytes displayed malfunction in the AD brain [158]. These increasing observations align with the proposition that enhanced glutamate levels in the extrasynaptic space are an important contributor to slowly developed excitotoxicity and neurodegeneration in AD development.

***Glutamatergic hyperactivity in ischemic stroke and AD***

Upon an acute ischemic attack, the sudden reduction in cerebral blood flow causes dramatic consequences in the affected region within minutes to a few hours, including a loss of oxygen supply and disrupted mitochondrial function, ATP depletion, and membrane depolarization, which collectively trigger augmented glutamate release and impaired uptake [146, 159]. The overall increase in glutamate concentration leads to the overactivation of glutamate receptors at synaptic and extrasynaptic sites of excitatory neurons, causing massive Ca^2+^ influx and ionic homeostasis disruption, cell swelling, and cell membrane deterioration, all of which are characteristics of necrotic cell death [31, 160, 161]. Cells surviving the initial ischemic insult may die a few days or weeks later in a “hybrid” form due to the activation of programmed cell death and aberrant autophagy pathways [162].

In addition to its well-documented roles in ischemia/hypoxia, eNMDAR activation has been implicated in the pathogenesis of neurodegenerative disorders, especially morphological and functional deteriorations in AD [153, 163, 164] and HD [109, 165]. Neuronal and NMDAR hyperactivity during the progression of AD and related dementia has regularly been detected in animal AD models as well as in AD patients [50, 51, 166, 167]. This abnormality occurs during the early stages of late-onset AD development [11, 12, 168]. The mechanism of action for the lasting time course and pathogenic effect of NMDAR hyperactivity, however, has been poorly defined. Intriguingly, brain hyperexcitability may not depend on amyloid plaque formation [169]. The sustained trigger of NMDAR hyperactivity at the initial stage of sporadic AD before endogenous Aβ deposition is largely unknown. Moreover, instead of hyperactivity, impaired NMDAR activity and signaling in the cortex and hippocampus can be observed in the aging/aged AD brain [170, 171]. This is a possible consequence of neurodegeneration as a result of chronic excitotoxicity and Aβ aggregation. In AD research, while the Aβ hypothesis has been demonstrated in different amyloid transgenic mouse models, basic research on the triggering and mediating mechanisms of neuronal toxicity have been solely focused on the toxic effects of amyloid and tau pathology. As a result, neuronal hyperactivity and NMDAR overactivation are attributed as consequences of the amyloid cascade both *in vitro* and in FAD transgenic mice [172-175]. This popular concept, however, has been challenged by compelling preclinical and clinical observations that neuronal and NMDAR hyperactivity can often be observed in the absence of amyloid deposition [33-36, 169].

***Activation of eNMDARs and tonic NMDAR currents***

In contrast to the phasic and intensive activation of sNMDARs, eNMDARs are associated with tonic NMDA currents induced by lower concentrations of extracellular glutamate. Consistent with their extrasynaptic locations, eNMDAR-mediated currents are insensitive to tetrodotoxin (TTX), which selectively blocks the synaptic release of glutamate [176, 177]. In line with their nonsynaptic nature, eNMDARs can be constitutively activated, and the activation may persist even in the absence of neuronal and/or synaptic activity [178]. In contrast, sNMDAR activity remains unaffected when tonic NMDA currents are blocked [176]. These characteristics clearly define two populations of NMDARs, one located inside and one located outside of the synaptic cleft.

Compatible with the lower concentrations, extrasynaptic glutamate preferably activates the main population of eNMDARs of higher affinity, e.g., GluN2B-containing NMDARs. GluN2B-containing eNMDARs were proposed to be responsible for ischemia-induced excitotoxicity [179, 180], and extrasynaptic glutamate is a primary contributor to ischemic and traumatic damage in the brain [143, 146, 181]. The activation of eNMDARs, perhaps together with the impaired protective function of sNMDARs, contributes to downstream cascades of necrotic and programmed cell death pathways [179, 182]. The tonic NMDA current is likely triggered by glutamate released mainly from astrocytes, which participate in neuron-glia communications, such as the regulation of neuronal excitability and synaptic strength that subsequently affect learning and memory formation [80, 134, 178]. The cellular properties of excitability and synaptic strength can be a part of neuroendocrine regulation as well as neuromodulatory actions or even sleep homeostasis [147].

Synaptic NMDAR activity is closely coupled to glutamate released from presynaptic vesicles and the clearance of glutamate from the synaptic cleft, while eNMDAR activation is characterized by exposure to chronic agonism by surrounding glutamate [183]. Ca^2+^ influx evoked by intense sNMDAR activation alone may not be harmful, as it can trigger genomic processes that render neurons more resistant to apoptotic and oxidative insults. It was shown, however, that long-term tonic activation of sNMDARs in hippocampal neurons under hypoxic conditions was able to induce excitotoxic cell death [104]. Thus, prolonged sNMDAR activation may also trigger pro-death signaling. In cultured cortical neurons, long-term, but not short-term, treatment with high-dose NMDA or oxygen-glucose deprivation triggered cell death and suppressed prosurvival signaling. The authors proposed that the co-activation of sNMDARs and eNMDARs is needed for excitotoxicity [106]. It is likely that any shift in balance to reduce sNMDAR or enhance eNMDAR signaling could be detrimental to neuronal viability [184, 185]. Thus, the NMDAR signaling inside and around the synapse must be maintained at a proper level so that it is enough to maintain neuronal activity and viability but not enough to become harmful so as to cause excitotoxic neurodegeneration [79, 154, 183].

Taken together, a large body of evidence supports the existence of a critical role for enhanced eNMDAR activity in the pathogenesis of stroke and AD [107, 108]. While the NMDAR localization hypothesis is not universally accepted, the majority of experimental data support that the stimulation of eNMDARs is a common early feature of acute and chronic neurological disorders. Acute and chronic excitotoxicity in stroke and neurodegenerative diseases, however, show distinct features in terms of glutamate intensity, time course, signaling pathways, and cell death mechanisms. Targeting eNMDARs can be a promising target for developing safe and effective therapies for these neurological disorders by decreasing extracellular glutamate spillover/release and tonic eNMDAR activation and ultimately maintaining the balance of synaptic and extrasynaptic NMDAR signaling under stressful and pathological conditions.

***Distinct signaling pathways associated with synaptic and extrasynaptic NMDARs***

The fact that different NMDAR subtypes locate at synapses and extrasynaptic sites raises the question of whether specific NMDAR subtypes and locations are responsible for distinct functions. Indeed, synaptic GluN2A-containing NMDARs are generally associated with cell survival, whereas extrasynaptic GluN2B-containing NMDARs are linked to cell death cascades [186, 187]. The activation of sNMDARs is related to the transcription of pro-survival genes and anti-apoptotic genes, which are favorable for cell survival through the phosphorylation of intracellular factors such as the transcription factor Cyclic-AMP response element binding protein (CREB) [188, 189]. Consistent data suggest that ERK1/2 is activated and inactivated by sNMDARs and eNMDARs, respectively [190]. The Ca^2+^ influx mediated by sNMDARs leads to consequent Ca^2+^ release from internal stores, generating Ca^2+^-activated kinases and transcription factors in the nucleus, such as Ca^2+^-calmodulin kinase IV (CaMKIV) and CREB, while inhibiting the transcription factor FOXO3α [189, 191]. The L-type voltage-gated channel is another main player in mediating the Ca^2+^ elevation in this signaling pathway [192]. The transcription induction results in the expression of brain-derived neurotrophic factor (BDNF) [189]. The activation of sNMDARs also increases Wnt/MAPK/ERK1/2 survival signaling activity [190, 193] and PI3K/Akt activity to promote the inhibition of FOXO [191]. JACOB is a caldendrin binding partner and a synapto-nuclear signaling protein [194]; ERK1/2 and the phosphorylation of JACOB appear to play a major role in communicating the origin of NMDAR activity to the nucleus, referred to as the synapto-nuclear trafficking/signaling [194, 195] (Fig. 3). On the other hand, the malfunction of extrasynaptic glutamate signaling is an important contributor to several pathophysiological conditions, including hyperexcitability, spreading depression, neurodegeneration, neuroinflammation, and demyelination [196]. The activation of eNMDARs leads to cell death by inhibiting survival signaling as well as promoting pro-death mechanisms such as the expression of cleaved caspase-3 [153, 197], the suppression of the CREB, p38 MAPK, and ERK1/2 pathways [190, 198], the activation of the FOXO transcription factor associated with AD neuropathology [191], and calpain-mediated cleavage of striatal-enriched protein tyrosine phosphatase (STEP) [79, 195, 199-201] (Fig. 3 and 4).

Glutamate-induced activation of eNMDARs in cultured neurons is required to disrupt the mitochondrial membrane potential associated with excitotoxic injury [79], which is likely mediated by GluN2B-containing eNMDARs [100]. Mounting data indicate that both NMDA receptor dysfunction and mitochondrial impairment are present in AD patients, animal models, and cell culture models. In neurons, Aβ and altered NMDAR function are linked with mitochondrial dysfunction through the dyshomeostasis of mitochondrial Ca^2+^ following Ca^2+^ influx mediated by GluN2B-NMDARs [202]. NMDAR-related mitochondrial dysfunction leads to increased production of reactive oxygen species (ROS), altered Ca^2+^ homeostasis, and decreased ATP production, providing a pathological link between eNMDARs, metabolism, and neurodegeneration [154, 203, 204] (Figs. 3 and 4). On the other hand, sNMDAR activity is necessary to boost intrinsic antioxidant defenses, which may further explain its neuroprotective effect against the progression of pathological processes associated with oxidative damage [205].

***Ca^2+^-associated excitotoxicity in ischemic stroke and AD***

Ca^2+^-induced excitotoxicity was initially described in neuronal cultures and animal models of ischemic stroke in investigations of glutamate-induced neuronal cell death [111, 206-208]. This type of cell death was characterized by drastic activation of glutamate receptors, mainly NMDARs, by excessive amounts of extracellular glutamate due to augmented synaptic release and impaired uptake mechanisms [209, 210]. The overstimulation of NMDARs results in massive Ca^2+^ influx and [Ca^2+^]_i_ overload [211, 212], and acute neuronal injury due to necrotic cell death [110, 117, 213] (Figs. 3). Consistent evidence has shown that Ca^2+^ entry through NMDARs was particularly more effective at killing neurons than entry through other receptors and channels [111, 146, 159, 160, 207, 208, 214]. Continual investigations also identified that, in conjunction with necrotic damage, programmed cell cascades such as apoptosis, aberrant autophagy, and the activation of several other cellular death mechanisms could take place concurrently or consequently as a part or consequence of excitotoxicity [162, 215-217].

In neurogenerative diseases such as AD and HD, no detectable acute excitotoxicity exists, while delayed and continuous neuronal loss are the hallmarks of neurodegeneration in those patients’ brains [218]. To explain the pathophysiology of chronic neuronal degeneration, the Ca^2+^ hypothesis proposed that moderate yet persistent [Ca^2+^]_i_ increases can cause excitotoxic neuronal damage in neurodegenerative diseases [127, 219-221]. Accumulating evidence supports that AD pathophysiology includes a chronic “calciumopathy” caused by NMDAR overactivation and Ca^2+^-induced excitotoxicity [11, 12, 168]. It was proposed that small but sustained increases in [Ca^2+^]_i_ activate Ca^2+^-dependent deleterious signals as key events or even causal factors of AD development [11, 12, 168, 222] (Figs. 4 and 5). The long-lasting (months in rodents and years/decades in humans) excitatory stresses are associated with not only increased Ca^2+^-activated signaling pathways but also chronic metabolic and inflammatory burdens, slowly progressing neuronal loss, and ultimately morphological and functional deterioration, including cognitive symptoms (Figs. 4 and 5). In line with this cascade, CREB phosphorylation at serine 133, which is required for its transcriptional activity and cell survival, is decreased (“shut-off” of the CREB signaling) after eNMDAR stimulation and in AD [79, 200]. At present, how NMDAR activity is persistently upregulated in the seemingly normal brain and its precise link to AD progression are poorly understood. Current research in this area has exclusively examined the NMDAR GluN1 and GluN2 subunits. For example, an increase in GluN2B expression was attributed to Aβ-induced NMDA hyperactivity [223].

Our recent investigation demonstrated for the first time that the deficiency of GluN3A plays a life-long pathogenic role in causing mild but persistent activation of eNMDARs, chronic Ca^2+^ dyshomeostasis, and degenerative excitotoxicity [127], as seen in clinical late-onset AD/ADRD patients [127, 224] (Fig. 5). GluN3A knockout (KO) mice age-dependently developed virtually all pathophysiological features of neurodegeneration and AD pathology, including programmed cell death signaling activation, chronic neuronal loss, synaptic disruption, LTP suppression, and early symptoms of olfactory deficits followed by progressive psychological and cognitive deficits [127]. In contrast to current FAD models and support an Aβ-independent AD mechanism, endogenous amyloid deposition and tau hyperphosphorylation spontaneously emerged in the GluN3A KO brain after, but not before, cognitive decline [127] (Fig. 5). Additionally, our recent study verified that selective knockout of GluN3A in the cortex and hippocampus of young adult mice (3 months old) using the CRISPR/Cas9 method resulted in similar AD-like morphological and functional alterations 3-6 months later [225]. The expression (knock-in) of GluN3A in the global GluN3A KO brain effectively prevented AD progression [225]. We also showed that early MEM treatment in GluN3A KO mice of 3-month old before cognition decline and Aβ deposition significantly prevented the age-dependent AD development [127]. Based on observations in this novel late-onset sporadic AD model, we propose that neuronal hyperactivity mediated by chronic up-regulation of eNMDAR activity, slight but persistent [Ca^2+^]_i_ increases and resulted chronic excitotoxicity in the cortex and hippocampus are a causal pathogenic mechanism of AD/ADRD (Fig. 5).

***A novel mediatory pathway downstream to NMDAR activation and Ca^2+^ increases***

Transient receptor potential cation channel subfamily M members (TRPMs) have been identified as key modulators of numerous Ca^2+^-dependent mechanisms such as the immune response, insulin secretion, myogenic tone of the cerebral artery, capillary fragmentation, and respiratory rhythm regulation [226]. Among them, TRPM4 is a Ca^2+^-activated monovalent cation channel [227, 228]. More recently, an interaction between NMDARs and TRPM4 was identified in NMDA-induced acute excitotoxicity. It is proposed that excitotoxicity requires the physical interaction of NMDARs and TRPM4, via intracellular domains in the near-membrane portions of the receptors [229]. The disruption of the NMDAR/TRPM4 complex does not affect NMDAR-mediated [Ca^2+^]_i_ increases, suggesting that the NMDAR-TRPM4 complex does not affect NMDAR activity or Ca^2+^ permeability, rather it influences downstream events following NMDAR activation. [228]Meanwhile, blocking NMDAR-TRPM4 interactions reduces NMDA toxicity and mitochondrial dysfunction, activates CREB and ERK1/2, boosts gene induction, and reduces neuronal loss in mouse models of stroke and retinal degeneration [229]. Interestingly, the NMDAR-TRPM2 coupling promotes the surface expression of eNMDARs, ultimately leading to increased neuronal death [230], while MEM treatment modulates TRPM2-induced excessive [Ca^2+^]_i_, hypoxia-mediated ROS production, and apoptosis [231]. It was also proposed that multiple TRPM2-mediated cellular and molecular mechanisms cause Aβ and/or oxidative damage in AD pathologies [232]. The novel mechanism of neuronal death shines a light on a regulatory pathway downstream to NMDAR activation, providing a possible neuroprotective strategy against excitotoxicity without directly blocking NMDARs [230]. [233][234]

***Interactions between NMDAR and Aβ: Alternative consequences of pathogenic events***

According to the amyloid hypothesis of AD, Aβ-triggered neuronal hyperactivity and NMDAR abnormalities have been extensively investigated for many years. For example, Aβ1-42 increased NMDAR-mediated Ca^2+^ influx [233, 234], and extracellular Aβ oligomers can bind to NMDARs containing the GluN2B subunit and mGluR1, consequently leading to synaptic disruptions [235, 236]. The Aβ effect was mediated by interaction with NMDARs, either directly or via synaptic proteins such as PSD95 [154, 237-239]. Aβ can increase NMDAR activity, Ca^2+^ influx, and Aβ-associated synaptic loss [240-242]. In transgenic AD animal models, NMDAR hyperactivity occurs following Aβ accumulation [243]. Aβ may directly activate GluN2A-containing NMDARs [244], and Aβ oligomers can evoke [Ca^2+^]_i_ rise through activated NMDARs in cortical neurons [233]. Numerous studies have shown that soluble Aβ causes a reduction in synaptic glutamatergic transmission and inhibits synaptic plasticity. For example, applying Aβ_1–42_ in cultured cortical neurons leads to the internalization of sNMDARs and the depression of NMDAR-mediated currents [245]. Aβ can also stimulate glutamate release from glial cells and activate eNMDARs [153]; glutamate release from cultured microglia and astrocytes were significantly greater in Aβ-treated cultures [246]. Detailed information about the effects of Aβ on NMDARs and neuronal activity can be found in several excellent reviews [54, 244, 247-249].

Extrasynaptic NMDAR activity promotes tau protein overexpression in neuronal cultures, and tau ablation is protective against cell death mediated by eNMDARs [250]. The promoting effect on tau phosphorylation is mediated by various kinases linked to augmented tau toxicity [153, 251]. Glycogen synthase kinase 3β (GSK-3β) is a tau kinase that is activated by Aβ [252] and contributes to Aβ-induced tau phosphorylation and toxicity [253]. Exacerbated tau toxicity associated with Aβ-induced GSK-3β activation can be prevented by inhibiting GluN2B-containing NMDARs [253]. Consistently, the Aβ impairment of axonal transport is significantly attenuated by NMDAR antagonists or by GSK-3β inhibition [254], supporting a functional link between NMDARs and GSK-3β activation. Interestingly, a recent study in tau knockout mice revealed that the deletion of tau decreased the eNMDA current in hippocampal neurons [255], which is consistent with the pro-degenerative roles of tau and eNMDARs via a similar receptor mechanism in AD.

More recent evidence suggests that sustained eNMDAR activation acts as an upstream event to Aβ production and secretion [241, 242] (Fig. 5). For example, overall NMDAR activation by bath NMDA application to cortical neuronal cultures increased the production and secretion of Aβ by upregulating the expression of APPs [256]. Synaptic activity and vascular exocytosis in the hippocampus drive the release of Aβ into the extracellular space [257], though the work did not identify involved NMDAR subtypes. In primary cultures of wild-type cortical neurons, prolonged stimulation of eNMDARs, but not sNMDARs, significantly increased the neuronal synthesis and release of Aβ [163]. This effect was preceded by a shift from APP695 (the neuronal isoform of APP) to KPI-APPs, which are isoforms exhibiting important amyloidogenic potential. The authors suggested that the eNMDAR pool is associated with APP and Aβ metabolism. Supporting this idea, there are significant overlaps between the signaling molecules implicated in AD and those influenced by eNMDAR stimulation. The effect of Aβ on JACOB translocation is entirely blocked by the GluN2B-specific antagonist ifenprodil, implying a mediating role of eNMDARs [258]. BDNF release from the implanted cells can attenuate cognitive deficits in AD mice, suggesting that BDNF deficiency may play an essential role in AD pathophysiology [259]. Furthermore, it was shown that BDNF induction is suppressed by eNMDAR activity [200]. Additionally, BDNF release from astrocytes is known to regulate the hyperactivation of neuronal populations via TrkB [260]. Importantly, this functional benefit of BDNF is achieved without improvement in either Aβ or tau pathology [259], suggesting a BDNF-dependent action downstream of the Aβ and tau cascade or an AD/tau independent mechanism. Endorsing these possibilities, viral delivery of CREB-binding protein (CBP) increases BDNF expression and improves cognitive function in an AD model without affecting Aβ or tau pathology [261].

As an underlying mechanism, sNMDAR activity increases α-secretase-mediated nonamyloidogenic processing of APP [262], while Ca^2+^ influx via persistent activation of eNMDARs leads to intranuclear CaMKIV activation and, via a series of signaling cascades, results in a shift from α-secretase to β-secretase-mediated APP processing and thereafter an increase in Aβ production [101]. It was further postulated that eNMDAR-promoted production of Aβ creates a toxic positive feedback loop in which Aβ enhances eNMDAR activity and stimulates Aβ production and secretion [107]. Collectively, accumulating evidence implicates that eNMDAR activation and imbalanced sNMDAR/eNMDAR activity tone are possible causal factors acting upstream of late-onset AD pathophysiology, including Aβ and tau pathology (Figs. 4 and 5).

***MEM is an eNMDAR antagonist with neuroprotective properties and few side effects***

For many years, the NMDAR contribution to excitotoxicity has represented attractive therapeutic targets for various CNS disorders. However, NMDAR antagonists that show promise in blocking excitotoxicity also disrupt normal synaptic functions, resulting in unacceptable side effects [30, 32, 263]. For instance, conventional NMDAR antagonists MK-801, phencyclidine (PCP), and ketamine induce some deteriorating actions such as schizophrenia-like symptoms in humans [264-266].

MEM is an uncompetitive low-affinity and use-dependent NMDAR antagonist with unique voltage and Mg^2+^ dependency that acts only at moderate depolarization [20, 52, 267, 268]. More importantly, unlike classic NMDAR antagonists, MEM preferentially acts on extrasynaptic GluN2B and GluN2C/2D containing NMDARs over synaptic NMDARs [99, 183, 269]. This is true, especially at therapeutic doses (1-10 µm *in vitro* and 1-30 mg/kg *in vivo*). Using Ca^2+^ imaging, it was demonstrated in primary cortical cultures that MEM significantly blocked the [Ca^2+^]_i_ increases mediated by eNMDARs and attenuated NMDA-induced neuronal cell death [99]. These unique pharmacological features of MEM imply minimal influence on the physiological activity of sNMDARs with effective suppression of overactivated eNMDARs.

MEM shows neuroprotective and neuroplasticity effects when it is administered acutely and chronically in stroke animals. In experimental acute treatments after permanent or transient ischemic stroke, MEM showed neuroprotective effects around 10-30 mg/kg (i.p., i.v. or oral) [270-273]. MEM can reduce ischemia-induced infarct formation and neuronal cell death acutely after an ischemic attack [271, 274]. In middle cerebral artery occlusion-reperfusion rats, MEM significantly prevented neuronal death by suppressing the activation of the calpain-caspase-3 pathway and apoptosis, consequently attenuating brain damage and neurological deficits [273]. In transient (60 min) ischemic stroke mice, low-dose MEM (0.2 mg/kg/day) started 24 hrs before stroke and continued for a 48-hour recovery period significantly reduced lesion volume by 30% to 50% and improved behavioral outcomes [275]. On the other hand, higher doses of MEM (20 mg/kg/day in this report) increased injury. The neuroprotective effect of MEM was also confirmed in stroke models using multiple species. In a rabbit multiple infarct embolic ischemia model, bolus injections of MEM at 25 mg/kg were lethal. However, slowly infused MEM was more tolerable and had substantial therapeutic benefits after acute ischemic stroke [276]. Like other NMDAR antagonists, delayed MEM treatments, e.g., 30-60 min after stroke, showed little neuroprotective effects [274, 276]. Unfortunately, pretreatment of MEM before stroke attacks is generally unpractical and hardly justifiable to apparently normal individuals.

*In vivo* studies verified that chronic oral MEM at clinical doses (1-30 mg/kg/day, for months to years) is well tolerated without significant neuronal or neurological abnormalities [20, 277-281]. Clinical trials with MEM have consistently demonstrated its safety for short- and long-term use, with an adverse event profile “similar to that of placebo” [282-286]. MEM may cause a few side effects that have been clinically well characterized; the most common adverse reactions include dizziness, headache, confusion, diarrhea, and constipation [287-289]. Other possible less common side effects include fatigue, pain, hypertension, weight gain, hallucination, confusion, aggression, vomiting, and urinary incontinence. These reactions are not life-threatening, and symptoms are treatable and reversible. However, as seen with most drug therapies, continual use of high doses of MEM (e.g., ≥30 mg/kg) may block synaptic and extrasynaptic NMDARs and can show side effects of neuronal loss and functional impairments [109].

MEM is thus far the only clinically approved NMDAR antagonist for the treatment of moderate-to-severe AD [282-286] (Table 1). The rationale for using MEM as a symptomatic treatment for advanced AD patients, but not as a disease-modifying early treatment, is in line with the previous judgment that NMDAR abnormalities are merely a consequence of Aβ/tau pathology. This justification, however, is noticeably inconsistent with mounting evidence from basic and clinical observations that NMDAR and neuronal hyperactivity are early and pathogenic mechanisms of AD development. According to the modified Ca^2+^ hypothesis of AD and recent evidence from our group and others that NMDAR overactivation and chronic Ca^2+^ dyshomeostasis are upstream events of AD pathology, including Aβ/tau alterations [127], it can be reasonably assumed that the marginal results of current MEM treatment in advanced AD patients are largely due to improper timing of the delayed treatment, which misses the pathogenic phase of neuronal hyperactivity ongoing for years during the prodromal/preclinical period of disease progression. A game-changing approach should be considered to start MEM treatment much earlier in individuals who show persistent signs of neuronal hyperactivities, Ca^2+^ dyshomeostasis, and other risk factors/early biomarkers of AD/ADRD (Fig. 6).

***Early MEM treatment as a disease-modifying therapy for AD and related dementia***

MEM treatments at early phase of incubation period is essential for a disease-modifying or preventive therapy in order to maintain year-long normal NMDAR activity and physiological Ca^2+^ levels in individuals vulnerable to AD/ADRD. In the GluN3A KO mouse, we demonstrated that presymptomatic MEM (10-20 mg/day) treatment from 3-month of age for 3 months prevented or attenuated AD brain neuropathology, Aβ production/aggregation, and cognation decline [127, 290]. Audrain et al. examined preventive treatment in a late-onset AD rat model. The early MEM (20 mg/daily) administration for 6 months started at asymptomatic phase of 4 months old promoted non-amyloidogenic cleavage of APP followed by a decrease in soluble Aβ42. MEM also prevented impairments of LTP and cognitive decline occurred in control AD rats, although tau hyperphosphorylation was unaffected [291]**.**

Thanks to the identification of risk factors and associated mutant genes in the human genome research [292-295], early and presymptomatic treatments become possible. Supporting this idea, clinical trials of MEM in mild cognitive impairment (MCI)/mild AD patients showed significant therapeutic benefits, such as maintained cognitive function and improved brain imaging findings [296-302] (Table 1); even so, MEM in these trials might not have been given early enough from presymptomatic phases. In fact, MEM has been frequently prescribed to MCI and mild AD/dementia patients based on many physicians’ own clinical experience [303]. Despite the emerging evidence, a meta-analysis review of data involving early MEM approaches concluded that early MEM treatments did not provide significant benefits for patients in trials analyzed. However, the authors also recognized that “Prospective trials are needed to further assess the potential for efficacy of memantine” [304]. Specifically, considering many failures of Aβ clearing therapies in clinical trials and documented benefits of MEM in MCI/AD patients, it is necessary to verify the effect of early preventive treatment using MEM and other eNMDAR antagonists in both preclinical and clinical investigations [298-302] (Fig. 6) (Table 1).

Ketamine is a non-competitive NMDAR antagonist, initially developed as an anesthetic drug. Besides multimodal analgesic actions, ketamine can induce a wide range of pharmacological effects, including neuroprotection, anti-inflammatory, anti-cancer, anti-depression/suicidal attempts, and status epilepticus [305-307]. In dose-dependent and brain status-dependent manners, ketamine displays neuroprotective or neurotoxic properties. At anesthetic doses applied during neurodevelopment, ketamine contributes to inflammation, autophagy, apoptosis, and enhances levels of reactive oxygen species [308]. On the other hand, a subanesthetic dose ketamine triggers multiple neurotrophic and neuroprotective effects mediated by NMDAR-dependent and -independent mechanisms. Regarding its anti-depression action, recent studies explored using ketamine to treat AD-related depression [309-311]. Esketamine, which is ketamine formulated as a nasal spray, was approved by the FDA as an adjuvant drug to be used for treatment-resistant depression (TRD) [312].

Being an NMDAR antagonist, ketamine has been tested in several stroke trials for safety and neuroprotective efficacy while no official reports are available up to now (Table 1). Despite potential ketamine’s cognitive effects, few clinical trials have examined its cognition benefits in AD patients. There has been no clinical trial to test ketamine as an anti-AD drug, mostly likely due to the current focus on the Aβ mechanism and concerns on possible side effects caused by prolonged use of ketamine.

***Early preventive neuroprotective treatments for AD and stroke in the same individuals***

Although multiple factors and shortcomings in preclinical and clinical research may contribute to the failure of NMDAR antagonists in stroke trials, one critical dilemma is the narrow therapeutic window. An NMDA receptor antagonist, even if it has few side effects, must be administered before or soon after (within a couple/few hours) the onset of ischemic attack to show protective effects in animal stroke models, which is generally impractical in clinical settings [313]. Aside from acute neuroprotective treatment, mounting evidence demonstrated that the brain and neuronal cells can be preconditioned using sublethal ischemia/hypoxia or a variety of chemicals/drugs to substantially enhance the tolerance to severe upcoming ischemic insults. This preconditioning strategy and its potent and broad cytoprotection have been confirmed in different animal models and human studies [314-316]. Like NMDAR antagonists, preconditioning treatment faces the same hurdle in that it requires pre-application well before the onset of an ischemic insult, and it is unfeasible to predict when somebody, even if he or she is known to be susceptible to stroke, will experience an ischemic attack in daily life.

Considering that stroke and AD share some key pathophysiological mechanisms with different time courses, a continual prophylactic pretreatment targeting common underlying NMDAR-related mechanisms at the early stages of AD is expected to be preventive for both stroke and AD, i.e., slowing AD progression while simultaneously priming the same brain against ischemic attacks that might strike at any time in the same aging individual (Fig. 6). There has been no such ideal treatment targeting both stroke and AD because it has been believed for a long time that the pathogenesis of AD is solely due to Aβ and tau pathology, which is fundamentally different from the cerebral ischemia that causes acute brain neurovascular damage.

To this end, we have performed the first investigation to test the preventive anti-stroke and anti-AD effects of MEM in the novel sporadic AD model of GluN3A KO mice as well as conventional 5XFAD mice [317]. Memantine (10 mg/kg/day in drinking water) was administered during the prodromal/preclinical stage to 3-month-old mice when olfactory deficits and neuronal hyperactivity were detectable but no cognitive dysfunction was present. After 3 months of treatment, significant benefits were observed in mice in the MEM group, showing a slowing of AD neuropathology and functional deterioration. Focal ischemic stroke was then induced in AD mice with and without MEM treatments to mimic the strokes commonly occurring in over 50% of AD patients. Compared to the vehicle group, the infarct volume and neuronal loss in AD mice that received 3 months of MEM treatment were significantly reduced 3 days after stroke. Continual monitoring and inspection of these animals 3 months later revealed less neurodegeneration and fewer cognitive deficits in the AD-stroke mice that received long-term MEM treatments. This ongoing investigation provides the first supporting evidence that early MEM treatment can be a preventive/preconditioning therapy for aging individuals susceptible to stroke and AD. Based on previous and our investigations, this innovative early approach is mechanistically justified, clinically feasible, and bears great clinical significance. More basic and preclinical research studies will help to reveal the detailed mechanisms of the dual effects of MEM against acute and chronic excitotoxicity. Clinical trials using MEM as a preventive therapy at the prodromal and mild MCI/AD stages should be carried out, and the effect of MEM on the prevalence and severity of stroke in AD patients should be explicitly analyzed and compared with that in patients not taking MEM. Meanwhile, the interaction between NMDARs and TRPMs as a downstream pathway to excitotoxicity provides another target for producing effective therapies against stroke and AD/ADRD. A better understanding of its physiological functions will help to predict the safety and efficacy of this approach.

***Development of selective eNMDAR antagonists for enhanced therapeutic benefits***

There has been increasing enthusiasm for the development of eNMDAR antagonists as potential treatments for stroke or neurodegenerative diseases such as AD and HD [107, 108]. Most of these compounds were MEM derivatives, such as MN-08 (a MEM nitrate) [153, 268, 318], fluoroethylnormemantine (FENM) [319, 320], and NitroSynapsin [153]. *In vitro* experiments with MN-08 demonstrated its anti-NMDAR effect and reduced Ca^2+^ influx, regulation of the ERK and PI3K/Akt/GSK3β pathways, and attenuation of glutamate-induced neuronal loss. In APP/PS1 transgenic mice and 3xTG-AD mice, several months of MN-08 daily treatments attenuated Aβ accumulation, neuronal and dendritic spine loss, and cognitive deficits. In addition, MN-08 had favorable pharmacokinetics, blood-brain barrier penetration, and safety profiles in rats and beagle dogs. These findings suggest that the novel memantine nitrate MN-08 may be a useful therapeutic agent for AD [321]. To improve the therapeutic potential and benefits of MEM, the Lipton group generated a series of drugs known as NitroMemantines, including the derivative of MEM-designated NitroSynapsin [153, 268, 318]. NitroSynapsin is a chemical adduct between an aminoadamantane moiety and a nitro group. Unlike MEM, NitroSynapsin acts as a dual-allosteric antagonist of eNMDARs, with aminoadamantane serving to target the nitro group to redox-modulatory/inhibitory sites on the extracellular surface of the receptor via S-nitrosylation. The pharmacological and therapeutic properties of NitroSynapsin have been examined and compared with those of MEM through *in vitro* and *in vivo* experiments. Patch clamp single-channel recordings confirmed that, like MEM, NitroSynapsin is a selective eNMDAR antagonist and can antagonize α-synuclein-induced synaptic damage and neuronal loss [322]. Human iPS cells (hiPSCs) and organoids bearing familial AD mutations exhibit aberrant electrical activity manifested as increased spontaneous action potentials, slow oscillatory events, and hypersynchronous network activity. NitroSynapsin, but not MEM, abrogated this hyperactivity [323].

To improve the specificity of the action of MEM on eNMDARs, a bioengineering approach was taken to design a hybrid nano-compound (AuM) with MEM attached via polymer linkers to a gold nanoparticle, the size of which is larger than the synaptic cleft [324]**.** AuM efficiently and selectively inhibited eNMDARs without inhibiting sNMDARs, and in comparison to MEM, AuM exhibited superior neuroprotective properties against NMDA-induced excitotoxicity and Aβ oligomer-induced dendritic spine loss [324]. This interesting drug design may represent a novel rational strategy for a new class of neuroprotective drugs with enhanced selectivity for eNMDARs that are effective in the treatment of stroke and neurodegenerative diseases.

Neramexane, a noncompetitive moderate open channel NMDAR antagonist as well as an inhibitor of cholinergic nicotinic receptors, has been shown to be efficient in enhancing long-term memory in adult rats and well tolerated in humans, suggesting potential therapeutic applications [325, 326]. In a clinical trial of the treatment of tinnitus, four weeks of 50 mg/day neramexane significantly improved functional scores compared to placebo [327]. Some early phase II/III clinical trials with neramexane for moderate-to-severe AD, however, showed contradictory results [326], which may be a reflection of the broader effects of neramexane and the timing of the drug administration, as discussed in this review.

Ifenprodil, a specific GluN2B receptor antagonist, prevents Aβ-induced endoplasmic reticulum (ER) stress, hippocampal dysfunction, and microtubule deregulation as well as Ca^2+^ rise [233]. Ifenprodil also prevents Aβ-induced inhibition of LTP, impairment of synaptic transmission, and retraction of synaptic contacts [328]. In acute hippocampal slices, the selective GluN2B antagonists ifenprodil and Ro25-6981 efficiently rescued LTP inhibition caused by soluble Aβ [328]. These results suggest that targeting the GluN2B subunit of NMDARs may be a promising way to prevent AD progression.

The compound 4-(5-(4-bromophenyl)-3-(6-methyl-2-oxo-4-phenyl-1,2-dihydroquinolin-3-yl)-4,5-dihydro-1H-pyrazol-1-yl)-4-oxobutanoic acid (DQP-1105) is a representative member of a new class of NMDAR antagonists and shows a preferred effect on GluN2C/2D subunits [329]. DQP-1105 was more potent for blocking currents evoked by bath-applied NMDA than for blocking synaptic NMDA currents. Thus, DQP-1105, like MEM, seems to have the potential to provide efficacy in therapeutic treatment while displaying minimal side effects.

With high clinical feasibility, the clinical drug lithium, which has been used for treating bipolar disease and depression, has drawn increasing attention for its multifaceted neuroprotective and regenerative mechanisms in the treatment of neurodegenerative diseases [330, 331]. Among its effects on cellular and molecular signaling pathways, lithium has been shown to reduce free radical-induced neurotoxicity and stabilize aberrant Ca^2+^ dyshomeostasis by an inhibitory action at NMDARs [332-334]^.^ Lithium prevents intracellular Ca^2+^ overload by suppressing IP3R-mediated ER Ca^2+^ release, subsequently attenuating Aβ accumulation and tau hyperphosphorylation and rescuing impaired hippocampal synaptic plasticity [335, 336]. Lithium is also a GSK3β inhibitor [337], which can be an underlying mechanism to attenuate Aβ-induced tau phosphorylation and toxicity [253]. Lithium is a tolerable drug, and its anti-excitotoxicity and anti-AD properties merit further investigation.

**Conclusion:**

Excitatory hyperactivity associated with the imbalance of the excitatory/inhibitory activity and overactivation of NMDARs, especially eNMDARs, and increased [Ca^2+^]_i_ cause the acute and chronic excitotoxicity of brain injuries such as ischemic stroke and neurodegenerative diseases such as AD [185, 338]. These decisive roles of NMDARs position them as major molecular and cellular players in critical brain functions and pertinent potential therapeutic targets for neurological disorders [61]. However, the cause of degenerative excitotoxicity and distinctions between acute and chronic forms of excitotoxicity has not been explicitly defined. Retrograde assessments regarding stroke prevalence and outcomes in MCI/AD/ADRD patients who were and were not prescribed chronic MEM treatments may provide indicative evidence for the dual efficacy of MEM; this clinical analysis, however, has so far not been performed.

A growing consensus considers that Aβ pathology is unlikely the initial pathogenic mechanism, at least not the only pathogenesis, for late-onset AD/ADRD. A better understanding of the causal mechanism of Aβ deposition, glutamatergic hyperactivity and downstream cascades, either in Aβ-dependent or -independent manners, will shine a new light onto the initial pathogenesis and aid in the development of early, preventive/preconditioning drug therapies with dual effects against late-onset AD/ADRD and ischemic stroke that often occur in the same individuals.

**List of abbreviations**

AD: Alzheimer’s disease

AMPA: α-amino-3-hydroxy-5-methyl-4-isoxazolepropionic acid

APP: amyloid precursor protein

ARIA: amyloid-related imaging abnormality

ARIA-E: amyloid-related imaging abnormalities (ARIAs)-cerebral edema

ARIA-H: ARIA-related microhemorrhages

Aβ: β-amyloid

BDNF: brain-derived neurotrophic factor

[Ca^2+^]_i_ : intracellular free Ca^2+^

CaMKII: Ca^2+^-calmodulin kinase II

CaMKIV: Ca^2+^-calmodulin kinase IV

CBP: CREB-binding protein

CREB: Cyclic-AMP response element binding protein

CNS: central nervous system

DQP-1105: 4-(5-(4-bromophenyl)-3-(6-methyl-2-oxo-4-phenyl-1,2-dihydroquinolin-3-yl)-4,5-dihydro-1H-pyrazol-1-yl)-4-oxobutanoic acid

EAATs: excitatory amino acid transporters

eNMDARs: extrasynaptic NMDARs

FDA: Federal Drug Administration

GSK-3β: glycogen synthase kinase 3β

FAD: familial AD

FENM: fluoroethylnormemantine

HD: Huntington’s disease

hiPSCs: human iPS cells

KO: knockout

LTD: long-term depression

LTP: long-term potentiation

MEM: memantine

MCI: mild cognitive impairment

NMDARs: N-methyl-D-aspartate receptors

PCP: phencyclidine

PSD: postsynaptic density

PSD: post-stroke dementia

rTPA: recombinant tissue plasminogen activator

ROS: reactive oxygen species

sNMDAR: synaptic NMDARs

STEP: striatal-enriched protein tyrosine phosphatase

TRD: treatment-resistant depression

TRPM: transient receptor potential melastatin

TTX: tetrodotoxin

vGlut: vesicular glutamate transporter

**Declarations**

- Ethics approval and consent to participate

Not applicable

- Consent for publication

Not applicable

- Availability of data and materials

All data and material reviewed in this article are available from the PubMed online search.

- Competing interests

The authors declare that they have no competing interests

- Funding

This publication was made possible by support from NIH/NINDS under award numbers R21AG067473, R01 NS114221, and from the Department of Veterans Affairs under award numbers RX001473 and RX003865.

- Authors' contributions

SY initiated, wrote, and revised the text and figures, and approved the submission of this review article; MJ participated in editing the text and contributed to making figures and revision of the paper; SS participated in editing and revision of the paper; SP generated Table 1, participated in editing and revision of the paper and figures; LW participated in editing, revision, and approved submission of the paper. All authors have read and approved the final manuscript.

- Acknowledgments

Not applicable

- Authors' information

The corresponding authors SY and LW are Endowed Professors at Emory University. MJ and SP are Postdoctoral Fellows at Emory University, SS is a Psychiatrist and Research Scientist at Atlanta VA Medical Center. Being the first and corresponding author, SY made primary contributions in preparing and writing this review. He is experienced in the basic and translational research on the mechanism and innovative therapy of stroke and Alzheimer’s disease. His previous investigations contributed to the identification of critical roles of ionic mechanisms involving potassium channels and NMDA receptors in excitotoxicity, programmed cell death, and neurodegeneration. His longstanding interest in NMDA receptor regulation has inspired examinations of the long-term pathophysiological features and age-dependent functional deficits of the GluN3A knockout mouse. The 2013 paper in *Journal of Physiology* [118] was the first report to show GluN3A (NR3A) is a pivotal regulator of cognitive function in adult mice. In the following papers, his group revealed the neuroprotective effect of GluN3A against ischemic stroke in adult animals [117], and a key role of the GluN3A expression in the olfactory and psychological functions associated with neurodegenerative diseases [116, 339]. Their most recent paper reported that a GluN3A deficiency induced spontaneously developed AD-like pathophysiology, psychological and cognitive declines, followed, but not preceded, by endogenous Aβ and tau pathology [127]. The mouse model is regarded as a lively support to the Ca^2+^ hypothesis of AD with the modification that the Ca^2+^-induced chronic excitotoxicity is closely associated with neurodegeneration and a pathogenic Aβ-independent mechanism of late-onset AD and related dementia.

**Figure legends**

***Figure 1. Composition of NMDA receptors and the regulatory role of the GluN3 subunit***

Functional NMDA receptors are transmembrane heterotetramers embedded in the phospholipid bilayer of glutamatergic neurons, containing two GluN1 and two GluN2 subunits. The binding of the ligand leads to the opening of the receptor cation channel in an Mg^2+^- and voltage-depolarization manner. The NMDAR activity and its mediated Ca^2+^ influx have significant impacts on synaptic transmission, neuronal plasticity, psychological/cognitive functions, and cell fates. A GluN3 (GluN3A and 3B) subunit can replace one GluN2 in the triheteromeric complex, resulting in restrained single-channel opening activities and smaller whole-cell currents compared to GluN1/GluN2 receptors. The NMDA current traces are our unpublished data, which were recorded in an Mg2^+^-free extracellular solution.

***Figure 2. Age-dependent subunit alternations of synaptic and extrasynaptic NMDA receptors and functional consequences***

NMDA receptors are mainly located in the post-synaptic membrane inside and outside of the synaptic cleft. Synaptic NMDARs are directly involved in excitatory neurotransmission and synaptic plasticity, while extrasynaptic NMDARs have regulatory roles in these activities. Glutamate concentrations are markedly different between the cleft and extrasynaptic spaces. Glutamate released by astrocytes and microglia (not shown) are likely the main components of extrasynaptic glutamate, together with that spillover from the synaptic cleft. The distribution and topography of NMDARs are subjected to age-dependent alterations. In addition to a developmental switch of increased GluN2A/GluN2B ratio [340, 341], the GluN3 expression also undergoes an age-dependent change, from the initial locations of both synaptic and extrasynaptic sites to extrasynaptic/peri-synaptic site in the mature brain. This developmental change is likely associated with the functional needs of NMDAR regulation at different life stages. For example, high levels of GluN3A in immature brains is neuroprotective; while in the adult brain, the absence of GluN3 in the synaptic site allows synapse maturation and elevated plasticity. In the adult brain, GluN3 remains to exist in the extrasynaptic membrane as an endogenous neuroprotective mechanism against brain damage and neurodegeneration. In the aging/aged or degenerative brain, loss or weakening of this regulatory mechanism due to either increased GluN2B expression or deficiency of GluN3 will lead to enhanced eNMDA activity and Ca^2+^ dysregulation, which aggravate acute and chronic excitotoxicity associated with ischemic stroke and late-onset AD.

**Figure 3. Distinctive Pro- and anti-survival mechanisms downstream from activation of synaptic and extrasynaptic NMDARs**

This simplified graph illustrates a few main signaling pathways associated with the activation of synaptic and extrasynaptic NMDA receptors, respectively. Of note that although additional genes not mentioned in the text are shown in the graph, not all related signals can be included in the graph. For example, chronic stress of neuronal hyperactivity and Ca^2+^ elevations induce recurrent inflammation that is not shown here. In general, activation of sNMDARs leads to pro-survival effects beneficial for neuronal viability and synaptic plasticity, while activation of eNMDARs causes detrimental consequences associated with acute and chronic excitotoxicity. It is worth mentioning, however, that many signaling genes such as CaMK and MAPK kinases can play opposite actions most likely in subtype-dependent manners. In the pro-survival mechanism, the Wnt regulation of the expression of CaMKIV is an upstream protective signaling in neurodegenerative conditions [342]. Cyclic-AMP response element binding protein (CREB) plays a key function in medicating sNMDAR activation and expressions of pro-survival genes such as BDNF, MAPK, and Akt. CREB phosphorylation is mediated acutely by CaMKIV while long-term regulation may be controlled upstream by ERK1/2 [343, 344]. Activation of CREB via CaMKIV phosphorylation of CREB binding protein (CBP) requires translocation of transducer of regulated CREB activity (TORC) which is downstream of Ca^2+^ signaling from sNMDAR activation. Jacob and the synapto-nuclear trafficking is a relatively new mechanism linking downstream signaling of sNMDARs. Caldendrin binds to Jacob's nuclear localization signal in a Ca^2+^-dependent manner [194, 195]. In contrast to these CREB-activating signals of sNMDARs, eNMDARs suppress CREB activity via the inactivation of the Ras-ERK1/2 pathway and the nuclear translocation of Jacob, which promotes CREB dephosphorylation. Calcineurin-dependent dephosphorylation of TORC and subsequent CREB activation is also downstream of sNMDAR transmission [345]. Activation of sNMDARs suppresses apoptotic cascades via suppression of the BH3-only domain gene Puma and p53, thereby limiting cytochrome c release. Downstream effectors of apoptosis including Apaf1, Caspase 3, and Caspase 9 are also suppressed [346, 347]. Contrary to these pro-survival pathways, pro-death pathways are mediated by downstream activities of eNMDARs [189, 348]. Interactions between the pro-survival and pro-death pathways may occur so that the suppression of CREB activity may result from inactivating the ERK1/2 pathway [79, 195]. Another shared pathway between synaptic and extrasynaptic NMDARs is the FOXO pathway. FOXO activity is suppressed by PI3K downstream of sNMDARs while activation of eNMDARs enhances FOXO nuclear import and the consequent transcription of FOXO3α, Bim, and Fas which lead to cell death via multiple mechanisms including excitotoxicity [205, 349]. Synaptic NMDAR activity enhances the transcription of PGC-1α, while excessive expression and activity of eNMDARs suppress CREB-dependent PGC-1α transcription [350]. In general, CaMKII is downstream of eNMDAR activity and acts as a carrier of Ca^2+^-regulated protease calpain to promote apoptotic cell death [351]. Moreover, Ca^2+^ dyshomeostasis resulting from NMDAR subunit composition such as GluN2B and GluN3A expression changes and its interaction with intracellular Ca^2+^ reservoirs in the ER and mitochondria play an important role in the maintenance of cellular bioenergetics, glucose metabolism, and normal mitophagy [352]. Ca^2+^ dyshomeostasis is thus a major trigger of the generation of ROS and increased apoptosis via the imbalance of mitochondria-initiated apoptotic genes including tBid, Bax/Bcl2, Bak/BclxL, Bad, Apaf1, cytochrome *c*, and caspases [353, 354]. The NMDAR-TRPM interaction is a novel cell death mechanism downstream to NMDAR and TRPM activation, which stimulates the formation of the NMDAR/TRPM complex in the extrasynaptic location. Excitotoxicity is then triggered by the complex in a “Ca^2+^-independent” fashion, mediated by mitochondrial dysfunction, reduced activation of ERK1/2, shut-off of the transcription factor CRAB, and cell death [229].

***Figure 4. Acute and chronic excitotoxicity and shared mechanisms between ischemic stroke and sporadic AD***

The sketch diagram illustrates similarities and differences between ischemic stroke and AD. Both brain disorders suffer from overactivations of eNMDARs that are subjected to regulations by glutamate concentration, expression of NMDAR subunits (e.g. GluN2 and GluN3), and other modulatory mechanisms. The vast and rapid Ca^2+^ influx upon cerebral ischemia and much mild but lasting Ca^2+^ stress in AD trigger distinctive Ca^2+^-dependent signaling pathways, leading to acute and chronic excitotoxicity, respectively. Depending on the severity and region of damage, ischemic stroke causes transient or permanent deficits of locomotor/sensorimotor activities and psychological/psychiatric/cognitive functions. Cerebral ischemia is also known for causing mitochondria dysfunction and ER Ca^2+^ stress that may be responsible for post-stroke AD-like pathology. On the other hand, chronic excitotoxicity in AD is induced by long-lasting small Ca^2+^ increases and deteriorating signaling pathways that lead to synaptic and neural network interruptions in specific regions critical for cognition, followed by Aβ deposition via increased activities of β- and δ-secretases [355]. This chronic excitotoxicity may cause late-onset AD in Aβ-dependent or -independent manner, which remains to be further investigated.

***Figure 5. NMDAR GluN3A deficiency induced sporadic AD***

The graph shows age-dependent events and corresponding experimental evidence in the NMDAR GluN3A knockout mouse. A GluN3A deficiency caused by genetic mutation or functional dysfunction can result in slight but persistent neuronal hyperactivity and [Ca^2+^]_i_ elevations, subsequently leading to chronic inflammation, metabolism burden, and slowly evolved degenerative excitotoxicity. The synaptic impairment and programmed neuronal cell death in the hippocampus and cortex are correlated to progressive cognitive decline. Interestingly and important to note that significant endogenous Aβ plague formation in neurons and blood vessels occurs after, but not before, cognition decline and other functional deficits [127].

***Figure 6. Hypothetic timelines of late-onset AD and common comorbidity of stroke***

Late-onset AD is a slow and progressive disease; its early pathophysiological cascades cultivate years to decades before clinical diagnosis and likely precede significant Aβ deposition which is a pathological event emerging in patients' brains of around 50 years old [48]. Different from the most popular diagram showing the events after Aβ deposition [356], this graph emphasizes possible triggering mechanisms before Aβ and tau pathology. In this hypothetic model, neuronal hyperactivity and Ca^2+^-associated chronic excitotoxicity exist well before neuronal loss, functional deficits, increased APP processing, and Aβ/tau pathology. Meanwhile, these underlying mechanisms significantly increase the risk of stroke attacks accompanied by acute excitotoxicity. Accordingly, a preventive disease-modifying intervention such as MEM treatment is necessary in the preclinical phase, which can also serve as a preconditioning therapy against stroke that strikes more than 50% of AD patients.

**Table Legend**

***Table 1. Clinical trials of NMDAR antagonists showing positive and promising potential for the treatment of ischemic stroke and AD/ADRD***

There have been over hundreds of clinical trials with NMDAR antagonists for the treatment of stroke and AD, respectively, most trials failed to show functional benefits in patients due to a variety of issues and dilemmas. This table was generated to briefly summarize promising NMDAR antagonists, focusing on recent trials of memantine and ketamine rather than providing a comprehensive list of trials. There has been no clinical trial of dual therapy against the comorbidity of stroke and AD/ADRD or MCI.

**References**:

1. Imran TF, Posner D, Honerlaw J, Vassy JL, Song RJ, Ho YL, Kittner SJ, Liao KP, Cai T, O'Donnell CJ, et al: **A phenotyping algorithm to identify acute ischemic stroke accurately from a national biobank: the Million Veteran Program.** *Clin Epidemiol* 2018, **10:**1509-1521.

2. Vijayan M, Reddy PH: **Stroke, Vascular Dementia, and Alzheimer's Disease: Molecular Links.** *J Alzheimers Dis* 2016, **54:**427-443.

3. Mijajlovic MD, Pavlovic A, Brainin M, Heiss WD, Quinn TJ, Ihle-Hansen HB, Hermann DM, Assayag EB, Richard E, Thiel A, et al: **Post-stroke dementia - a comprehensive review.** *BMC Med* 2017, **15:**11.

4. Henon H, Pasquier F, Leys D: **Poststroke dementia.** *Cerebrovasc Dis* 2006, **22:**61-70.

5. Association A: **2020 Alzheimer's disease facts and figures.** *Alzheimers Dement* 2020,

PMID: 32157811.

6. Kempuraj D, Ahmed ME, Selvakumar GP, Thangavel R, Raikwar SP, Zaheer SA, Iyer SS, Burton C, James D, Zaheer A: **Psychological Stress-Induced Immune Response and Risk of Alzheimer's Disease in Veterans from Operation Enduring Freedom and Operation Iraqi Freedom.** *Clin Ther* 2020, **42:**974-982.

7. Kalaria RN, Akinyemi R, Ihara M: **Stroke injury, cognitive impairment and vascular dementia.** *Biochim Biophys Acta* 2016, **1862:**915-925.

8. Zupanic E, von Euler M, Winblad B, Xu H, Secnik J, Kramberger MG, Religa D, Norrving B, Garcia-Ptacek S: **Mortality After Ischemic Stroke in Patients with Alzheimer's Disease Dementia and Other Dementia Disorders.** *J Alzheimers Dis* 2021, **81:**1253-1261.

9. Klomparens K, Ding Y: **Updates on the association of brain injury and Alzheimer's disease.** *Brain Circ* 2020, **6:**65-69.

10. Cechetto DF, Hachinski V, Whitehead SN: **Vascular risk factors and Alzheimer's disease.** *Expert Rev Neurother* 2008, **8:**743-750.

11. Mattson MP, Rydel RE, Lieberburg I, Smith-Swintosky VL: **Altered calcium signaling and neuronal injury: stroke and Alzheimer's disease as examples.** *Ann N Y Acad Sci* 1993, **679:**1-21.

12. Olney JW, Wozniak DF, Farber NB: **Excitotoxic neurodegeneration in Alzheimer disease. New hypothesis and new therapeutic strategies.** *Arch Neurol* 1997, **54:**1234-1240.

13. Howe MD, Atadja LA, Furr JW, Maniskas ME, Zhu L, McCullough LD, Urayama A: **Fibronectin induces the perivascular deposition of cerebrospinal fluid-derived amyloid-beta in aging and after stroke.** *Neurobiol Aging* 2018, **72:**1-13.

14. Shen MY, Chen FY, Hsu JF, Fu RH, Chang CM, Chang CT, Liu CH, Wu JR, Lee AS, Chan HC, et al: **Plasma L5 levels are elevated in ischemic stroke patients and enhance platelet aggregation.** *Blood* 2016, **127:**1336-1345.

15. Nguyen TV, Hayes M, Zbesko JC, Frye JB, Congrove NR, Belichenko NP, McKay BS, Longo FM, Doyle KP: **Alzheimer's associated amyloid and tau deposition co-localizes with a homeostatic myelin repair pathway in two mouse models of post-stroke mixed dementia.** *Acta Neuropathol Commun* 2018, **6:**100.

16. Hoyte L, Barber PA, Buchan AM, Hill MD: **The rise and fall of NMDA antagonists for ischemic stroke.** *Curr Mol Med* 2004, **4:**131-136.

17. Dai Y, Lei C, Zhang Z, Qi Y, Lao K, Gou X: **Amyloid-beta targeted therapeutic approaches for Alzheimer's disease: long road ahead.** *Curr Drug Targets* 2022, **23:**1040-1056.

18. Kim CK, Lee YR, Ong L, Gold M, Kalali A, Sarkar J: **Alzheimer's Disease: Key Insights from Two Decades of Clinical Trial Failures.** *J Alzheimers Dis* 2022, **87:**83-100.

19. Mangal R, Ding Y: **Mini review: Prospective therapeutic targets of Alzheimer's disease.** *Brain Circ* 2022, **8:**1-5.

20. Rogawski MA, Wenk GL: **The neuropharmacological basis for the use of memantine in the treatment of Alzheimer's disease.** *CNS Drug Rev* 2003, **9:**275-308.

21. Fleck LM: **Alzheimer's and Aducanumab: Unjust Profits and False Hopes.** *Hastings Cent Rep* 2021, **51:**9-11.

22. Yuksel JM, Noviasky J, Britton S: **Aducanumab for Alzheimer's Disease: Summarized Data From EMERGE, ENGAGE, and PRIME Studies.** *Sr Care Pharm* 2022, **37:**329-334.

23. Budd Haeberlein S, Aisen PS, Barkhof F, Chalkias S, Chen T, Cohen S, Dent G, Hansson O, Harrison K, von Hehn C, et al: **Two Randomized Phase 3 Studies of Aducanumab in Early Alzheimer's Disease.** *J Prev Alzheimers Dis* 2022, **9:**197-210.

24. Knopman DS, Jones DT, Greicius MD: **Failure to demonstrate efficacy of aducanumab: An analysis of the EMERGE and ENGAGE trials as reported by Biogen, December 2019.** *Alzheimers Dement* 2021, **17:**696-701.

25. Kuller LH, Lopez OL: **ENGAGE and EMERGE: Truth and consequences?** *Alzheimers Dement* 2021, **17:**692-695.

26. Waziry R, Chibnik LB, Bos D, Ikram MK, Hofman A: **Risk of hemorrhagic and ischemic stroke in patients with Alzheimer disease: A synthesis of the literature.** *Neurology* 2020, **94:**265-272.

27. Chi NF, Chien LN, Ku HL, Hu CJ, Chiou HY: **Alzheimer disease and risk of stroke: a population-based cohort study.** *Neurology* 2013, **80:**705-711.

28. Honig LS, Tang MX, Albert S, Costa R, Luchsinger J, Manly J, Stern Y, Mayeux R: **Stroke and the risk of Alzheimer disease.** *Arch Neurol* 2003, **60:**1707-1712.

29. Muir KW: **Glutamate-based therapeutic approaches: clinical trials with NMDA antagonists.** *Curr Opin Pharmacol* 2006, **6:**53-60.

30. Ikonomidou C, Turski L: **Why did NMDA receptor antagonists fail clinical trials for stroke and traumatic brain injury?** *Lancet Neurol* 2002, **1:**383-386.

31. Choi DW: **Excitotoxicity: Still Hammering the Ischemic Brain in 2020.** *Front Neurosci* 2020, **14:**579953.

32. Hardingham GE, Bading H: **The Yin and Yang of NMDA receptor signalling.** *Trends Neurosci* 2003, **26:**81-89.

33. Ricciarelli R, Fedele E: **The Amyloid Cascade Hypothesis in Alzheimer's Disease: It's Time to Change Our Mind.** *Curr Neuropharmacol* 2017, **15:**926-935.

34. Herrup K: **The case for rejecting the amyloid cascade hypothesis.** *Nat Neurosci* 2015, **18:**794-799.

35. Kametani F, Hasegawa M: **Reconsideration of Amyloid Hypothesis and Tau Hypothesis in Alzheimer's Disease.** *Front Neurosci* 2018, **12:**25.

36. Morris GP, Clark IA, Vissel B: **Inconsistencies and controversies surrounding the amyloid hypothesis of Alzheimer's disease.** *Acta Neuropathol Commun* 2014, **2:**135.

37. Vetrivel KS, Zhang YW, Xu H, Thinakaran G: **Pathological and physiological functions of presenilins.** *Mol Neurodegener* 2006, **1:**4.

38. Chen G, Chen KS, Knox J, Inglis J, Bernard A, Martin SJ, Justice A, McConlogue L, Games D, Freedman SB, Morris RG: **A learning deficit related to age and beta-amyloid plaques in a mouse model of Alzheimer's disease.** *Nature* 2000, **408:**975-979.

39. Frautschy SA, Yang F, Irrizarry M, Hyman B, Saido TC, Hsiao K, Cole GM: **Microglial**

**response to amyloid plaques in APPsw transgenic mice.** *Am J Pathol* 1998, **152:**307-317,

PMID: 9422548.

40. Whyte LS, Hemsley KM, Lau AA, Hassiotis S, Saito T, Saido TC, Hopwood JJ, Sargeant TJ: **Reduction in open field activity in the absence of memory deficits in the App(NL-G-F) knock-in mouse model of Alzheimer's disease.** *Behav Brain Res* 2018, **336:**177-181.

41. Mucke L, Masliah E, Johnson WB, Ruppe MD, Alford M, Rockenstein EM, Forss-Petter S, Pietropaolo M, Mallory M, Abraham CR: **Synaptotrophic effects of human amyloid beta protein precursors in the cortex of transgenic mice.** *Brain Res* 1994, **666:**151-167.

42. Kazee AM, Johnson EM: **Alzheimer's Disease Pathology in Non-Demented Elderly.** *J Alzheimers Dis* 1998, **1:**81-89.

43. Schmitt FA, Davis DG, Wekstein DR, Smith CD, Ashford JW, Markesbery WR: **"Preclinical" AD revisited: neuropathology of cognitively normal older adults.** *Neurology* 2000, **55:**370-376.

44. Goldman WP, Price JL, Storandt M, Grant EA, McKeel DW, Jr., Rubin EH, Morris JC: **Absence of cognitive impairment or decline in preclinical Alzheimer's disease.** *Neurology* 2001, **56:**361-367.

45. Mullane K, Williams M: **Alzheimer's disease beyond amyloid: Can the repetitive failures of amyloid-targeted therapeutics inform future approaches to dementia drug discovery?** *Biochem Pharmacol* 2020, **177:**113945.

46. Mullane K, Williams M: **Preclinical Models of Alzheimer's Disease: Relevance and Translational Validity.** *Curr Protoc Pharmacol* 2019, **84:**e57.

47. Aisen PS, Cummings J, Jack CR, Jr., Morris JC, Sperling R, Frolich L, Jones RW, Dowsett SA, Matthews BR, Raskin J, et al: **On the path to 2025: understanding the Alzheimer's disease continuum.** *Alzheimers Res Ther* 2017, **9:**60.

48. Golde TE: **Alzheimer's disease - the journey of a healthy brain into organ failure.** *Mol Neurodegener* 2022, **17:**18.

49. Hachinski V, Avan A: **A new definition of brain reserve.** *Alzheimers Dement* 2022, **18:**535-537.

50. Muller L, Kirschstein T, Kohling R, Kuhla A, Teipel S: **Neuronal Hyperexcitability in APPSWE/PS1dE9 Mouse Models of Alzheimer's Disease.** *J Alzheimers Dis* 2021, **81:**855-869.

51. Toniolo S, Sen A, Husain M: **Modulation of Brain Hyperexcitability: Potential New Therapeutic Approaches in Alzheimer's Disease.** *Int J Mol Sci* 2020, **21:**9318.

52. Parsons CG, Stoffler A, Danysz W: **Memantine: a NMDA receptor antagonist that improves memory by restoration of homeostasis in the glutamatergic system--too little activation is bad, too much is even worse.** *Neuropharmacology* 2007, **53:**699-723.

53. Haberman RP, Branch A, Gallagher M: **Targeting Neural Hyperactivity as a Treatment to Stem Progression of Late-Onset Alzheimer's Disease.** *Neurotherapeutics* 2017, **14:**662-676.

54. Ghatak S, Talantova M, McKercher SR, Lipton SA: **Novel Therapeutic Approach for Excitatory/Inhibitory Imbalance in Neurodevelopmental and Neurodegenerative Diseases.** *Annu Rev Pharmacol Toxicol* 2021, **61:**701-721.

55. Sen A, Akinola M, Tai XY, Symmonds M, Davis Jones G, Mura S, Galloway J, Hallam A, Chan JYC, Koychev I, et al: **An Investigation of Levetiracetam in Alzheimer's Disease (ILiAD): a double-blind, placebo-controlled, randomised crossover proof of concept study.** *Trials* 2021, **22:**508.

56. Hopkins Tanne J: **Approved drugs are to be studied for use in Alzheimer's disease.** *BMJ* 2016, **354:**i5063.

57. Rodriguez GA, Barrett GM, Duff KE, Hussaini SA: **Chemogenetic attenuation of neuronal activity in the entorhinal cortex reduces Abeta and tau pathology in the hippocampus.** *PLoS Biol* 2020, **18:**e3000851.

58. Traynelis SF, Wollmuth LP, McBain CJ, Menniti FS, Vance KM, Ogden KK, Hansen KB, Yuan H, Myers SJ, Dingledine R: **Glutamate receptor ion channels: structure, regulation, and function.** *Pharmacol Rev* 2010, **62:**405-496.

59. Sobolevsky AI: **Structure and gating of tetrameric glutamate receptors.** *J Physiol* 2015, **593:**29-38.

60. Greger IH, Mayer ML: **Structural biology of glutamate receptor ion channels: towards an understanding of mechanism.** *Curr Opin Struct Biol* 2019, **57:**185-195.

61. Morris RG: **NMDA receptors and memory encoding.** *Neuropharmacology* 2013, **74:**32-40.

62. Vyklicky V, Korinek M, Smejkalova T, Balik A, Krausova B, Kaniakova M, Lichnerova K, Cerny J, Krusek J, Dittert I, et al: **Structure, function, and pharmacology of NMDA receptor channels.** *Physiol Res* 2014, **63:**S191-203.

63. Paoletti P: **Molecular basis of NMDA receptor functional diversity.** *Eur J Neurosci* 2011, **33:**1351-1365.

64. Perez-Otano I, Larsen RS, Wesseling JF: **Emerging roles of GluN3-containing NMDA receptors in the CNS.** *Nat Rev Neurosci* 2016, **17:**623-635.

65. Kohr G: **NMDA receptor function: subunit composition versus spatial distribution.** *Cell Tissue Res* 2006, **326:**439-446.

66. Hansen KB, Ogden KK, Yuan H, Traynelis SF: **Distinct functional and pharmacological properties of Triheteromeric GluN1/GluN2A/GluN2B NMDA receptors.** *Neuron* 2014, **81:**1084-1096.

67. Stroebel D, Casado M, Paoletti P: **Triheteromeric NMDA receptors: from structure to synaptic physiology.** *Curr Opin Physiol* 2018, **2:**1-12.

68. Hammond EC: **Smoking in relation to the death rates of one million men and women.** *Natl Cancer Inst Monogr* 1966, **19:**127-204, PMID: 5905667.

69. Pachernegg S, Strutz-Seebohm N, Hollmann M: **GluN3 subunit-containing NMDA receptors: not just one-trick ponies.** *Trends Neurosci* 2012, **35:**240-249.

70. Henson MA, Roberts AC, Perez-Otano I, Philpot BD: **Influence of the NR3A subunit on NMDA receptor functions.** *Prog Neurobiol* 2010, **91:**23-37.

71. Fukumori R, Takarada T, Nakamichi N, Kambe Y, Kawagoe H, Nakazato R, Yoneda Y: **Requirement of both NR3A and NR3B subunits for dominant negative properties on Ca2+ mobilization mediated by acquired N-methyl-D-aspartate receptor channels into mitochondria.** *Neurochem Int* 2010, **57:**730-737.

72. Wada A, Takahashi H, Lipton SA, Chen HS: **NR3A modulates the outer vestibule of the "NMDA" receptor channel.** *J Neurosci* 2006, **26:**13156-13166.

73. Grand T, Abi Gerges S, David M, Diana MA, Paoletti P: **Unmasking GluN1/GluN3A excitatory glycine NMDA receptors.** *Nat Commun* 2018, **9:**4769.

74. Smothers CT, Woodward JJ: **Pharmacological characterization of glycine-activated currents in HEK 293 cells expressing N-methyl-D-aspartate NR1 and NR3 subunits.** *J Pharmacol Exp Ther* 2007, **322:**739-748.

75. Sadat-Shirazi MS, Vousooghi N, Alizadeh B, Makki SM, Zarei SZ, Nazari S, Zarrindast MR: **Expression of NMDA receptor subunits in human blood lymphocytes: A peripheral biomarker in online computer game addiction.** *J Behav Addict* 2018, **7:**260-268.

76. Palygin O, Lalo U, Pankratov Y: **Distinct pharmacological and functional properties of NMDA receptors in mouse cortical astrocytes.** *Br J Pharmacol* 2011, **163:**1755-1766.

77. Sproul A, Steele SL, Thai TL, Yu S, Klein JD, Sands JM, Bell PD: **N-methyl-D-aspartate receptor subunit NR3a expression and function in principal cells of the collecting duct.** *Am J Physiol Renal Physiol* 2011, **301:**F44-54.

78. Frokiaer J: **Collecting duct expression of N-methyl-D-aspartate receptor subtype NR3a regulates urinary concentrating capacity.** *Am J Physiol Renal Physiol* 2011, **301:**F42-43.

79. Hardingham GE, Fukunaga Y, Bading H: **Extrasynaptic NMDARs oppose synaptic NMDARs by triggering CREB shut-off and cell death pathways.** *Nat Neurosci* 2002, **5:**405-414.

80. Papouin T, Oliet SH: **Organization, control and function of extrasynaptic NMDA receptors.** *Philos Trans R Soc Lond B Biol Sci* 2014, **369:**20130601.

81. Zhou Q, Sheng M: **NMDA receptors in nervous system diseases.** *Neuropharmacology* 2013, **74:**69-75.

82. Brickley SG, Misra C, Mok MH, Mishina M, Cull-Candy SG: **NR2B and NR2D subunits coassemble in cerebellar Golgi cells to form a distinct NMDA receptor subtype restricted to extrasynaptic sites.** *J Neurosci* 2003, **23:**4958-4966.

83. Wee KS, Tan FC, Cheong YP, Khanna S, Low CM: **Ontogenic Profile and Synaptic Distribution of GluN3 Proteins in the Rat Brain and Hippocampal Neurons.** *Neurochem Res* 2016, **41:**290-297.

84. Berg LK, Larsson M, Morland C, Gundersen V: **Pre- and postsynaptic localization of NMDA receptor subunits at hippocampal mossy fibre synapses.** *Neuroscience* 2013, **230:**139-150.

85. MacDonald JF, Jackson MF, Beazely MA: **Hippocampal long-term synaptic plasticity and signal amplification of NMDA receptors.** *Crit Rev Neurobiol* 2006, **18:**71-84.

86. Liu L, Wong TP, Pozza MF, Lingenhoehl K, Wang Y, Sheng M, Auberson YP, Wang YT: **Role of NMDA receptor subtypes in governing the direction of hippocampal synaptic plasticity.** *Science* 2004, **304:**1021-1024.

87. Papouin T, Ladepeche L, Ruel J, Sacchi S, Labasque M, Hanini M, Groc L, Pollegioni L, Mothet JP, Oliet SH: **Synaptic and extrasynaptic NMDA receptors are gated by different endogenous coagonists.** *Cell* 2012, **150:**633-646.

88. Massey PV, Johnson BE, Moult PR, Auberson YP, Brown MW, Molnar E, Collingridge GL, Bashir ZI: **Differential roles of NR2A and NR2B-containing NMDA receptors in cortical long-term potentiation and long-term depression.** *J Neurosci* 2004, **24:**7821-7828.

89. Liu DD, Yang Q, Li ST: **Activation of extrasynaptic NMDA receptors induces LTD in rat hippocampal CA1 neurons.** *Brain Res Bull* 2013, **93:**10-16.

90. Yang Q, Zhu G, Liu D, Ju JG, Liao ZH, Xiao YX, Zhang Y, Chao N, Wang J, Li W, et al: **Extrasynaptic NMDA receptor dependent long-term potentiation of hippocampal CA1 pyramidal neurons.** *Sci Rep* 2017, **7:**3045.

91. Paoletti P, Bellone C, Zhou Q: **NMDA receptor subunit diversity: impact on receptor properties, synaptic plasticity and disease.** *Nat Rev Neurosci* 2013, **14:**383-400.

92. von Engelhardt J, Bocklisch C, Tonges L, Herb A, Mishina M, Monyer H: **GluN2D-containing NMDA receptors-mediate synaptic currents in hippocampal interneurons and pyramidal cells in juvenile mice.** *Front Cell Neurosci* 2015, **9:**95.

93. Yao L, Rong Y, Ma X, Li H, Deng D, Chen Y, Yang S, Peng T, Ye T, Liang F, et al: **Extrasynaptic NMDA Receptors Bidirectionally Modulate Intrinsic Excitability of Inhibitory Neurons.** *J Neurosci* 2022, **42:**3066-3079.

94. Riebe I, Seth H, Culley G, Dosa Z, Radi S, Strand K, Frojd V, Hanse E: **Tonically active NMDA receptors--a signalling mechanism critical for interneuronal excitability in the CA1 stratum radiatum.** *Eur J Neurosci* 2016, **43:**169-178.

95. Garst-Orozco J, Malik R, Lanz TA, Weber ML, Xi H, Arion D, Enwright JF, 3rd, Lewis DA, O'Donnell P, Sohal VS, Buhl DL: **GluN2D-mediated excitatory drive onto medial prefrontal cortical PV+ fast-spiking inhibitory interneurons.** *PLoS One* 2020, **15:**e0233895.

96. Garcia-Munoz M, Lopez-Huerta VG, Carrillo-Reid L, Arbuthnott GW: **Extrasynaptic glutamate NMDA receptors: key players in striatal function.** *Neuropharmacology* 2015, **89:**54-63.

97. Morishita W, Lu W, Smith GB, Nicoll RA, Bear MF, Malenka RC: **Activation of NR2B-containing NMDA receptors is not required for NMDA receptor-dependent long-term depression.** *Neuropharmacology* 2007, **52:**71-76.

98. Barria A, Malinow R: **NMDA receptor subunit composition controls synaptic plasticity by regulating binding to CaMKII.** *Neuron* 2005, **48:**289-301.

99. Leveille F, El Gaamouch F, Gouix E, Lecocq M, Lobner D, Nicole O, Buisson A: **Neuronal viability is controlled by a functional relation between synaptic and extrasynaptic NMDA receptors.** *FASEB J* 2008, **22:**4258-4271.

100. Stanika RI, Pivovarova NB, Brantner CA, Watts CA, Winters CA, Andrews SB: **Coupling diverse routes of calcium entry to mitochondrial dysfunction and glutamate excitotoxicity.** *Proc Natl Acad Sci U S A* 2009, **106:**9854-9859.

101. Bordji K, Becerril-Ortega J, Buisson A: **Synapses, NMDA receptor activity and neuronal Abeta production in Alzheimer's disease.** *Rev Neurosci* 2011, **22:**285-294.

102. Lipton SA: **Pathologically activated therapeutics for neuroprotection.** *Nat Rev Neurosci* 2007, **8:**803-808.

103. Bai N, Aida T, Yanagisawa M, Katou S, Sakimura K, Mishina M, Tanaka K: **NMDA receptor subunits have different roles in NMDA-induced neurotoxicity in the retina.** *Mol Brain* 2013, **6:**34.

104. Wroge CM, Hogins J, Eisenman L, Mennerick S: **Synaptic NMDA receptors mediate hypoxic excitotoxic death.** *J Neurosci* 2012, **32:**6732-6742.

105. Zhou X, Ding Q, Chen Z, Yun H, Wang H: **Involvement of the GluN2A and GluN2B subunits in synaptic and extrasynaptic N-methyl-D-aspartate receptor function and neuronal excitotoxicity.** *J Biol Chem* 2013, **288:**24151-24159.

106. Zhou X, Hollern D, Liao J, Andrechek E, Wang H: **NMDA receptor-mediated excitotoxicity depends on the coactivation of synaptic and extrasynaptic receptors.** *Cell Death Dis* 2013, **4:**e560.

107. Parsons MP, Raymond LA: **Extrasynaptic NMDA receptor involvement in central nervous system disorders.** *Neuron* 2014, **82:**279-293.

108. Bading H: **Therapeutic targeting of the pathological triad of extrasynaptic NMDA receptor signaling in neurodegenerations.** *J Exp Med* 2017, **214:**569-578.

109. Okamoto S, Pouladi MA, Talantova M, Yao D, Xia P, Ehrnhoefer DE, Zaidi R, Clemente A, Kaul M, Graham RK, et al: **Balance between synaptic versus extrasynaptic NMDA receptor activity influences inclusions and neurotoxicity of mutant huntingtin.** *Nat Med* 2009, **15:**1407-1413.

110. Choi DW: **Excitotoxic cell death.** *J Neurobiol* 1992, **23:**1261-1276.

111. Rothman SM, Olney JW: **Glutamate and the pathophysiology of hypoxic--ischemic brain damage.** *Ann Neurol* 1986, **19:**105-111.

112. Chung C, Marson JD, Zhang QG, Kim J, Wu WH, Brann DW, Chen BS: **Neuroprotection Mediated through GluN2C-Containing N-methyl-D-aspartate (NMDA) Receptors Following Ischemia.** *Sci Rep* 2016, **6:**37033.

113. Petralia RS: **Distribution of extrasynaptic NMDA receptors on neurons.** *ScientificWorldJournal* 2012, **2012:**267120.

114. Perez-Otano I, Lujan R, Tavalin SJ, Plomann M, Modregger J, Liu XB, Jones EG, Heinemann SF, Lo DC, Ehlers MD: **Endocytosis and synaptic removal of NR3A-containing NMDA receptors by PACSIN1/syndapin1.** *Nat Neurosci* 2006, **9:**611-621.

115. Roberts AC, Diez-Garcia J, Rodriguiz RM, Lopez IP, Lujan R, Martinez-Turrillas R, Pico E, Henson MA, Bernardo DR, Jarrett TM, et al: **Downregulation of NR3A-containing NMDARs is required for synapse maturation and memory consolidation.** *Neuron* 2009, **63:**342-356.

116. Lee JH, Wei L, Deveau TC, Gu X, Yu SP: **Expression of the NMDA receptor subunit GluN3A (NR3A) in the olfactory system and its regulatory role on olfaction in the adult mouse.** *Brain Struct Funct* 2016, **221:**3259-3273.

117. Lee JH, Wei ZZ, Chen D, Gu X, Wei L, Yu SP: **A neuroprotective role of the NMDA receptor subunit GluN3A (NR3A) in ischemic stroke of the adult mouse.** *Am J Physiol Cell Physiol* 2015, **308:**C570-577.

118. Mohamad O, Song M, Wei L, Yu SP: **Regulatory roles of the NMDA receptor GluN3A subunit in locomotion, pain perception and cognitive functions in adult mice.** *J Physiol* 2013, **591:**149-168.

119. Eriksson M, Nilsson A, Froelich-Fabre S, Akesson E, Dunker J, Seiger A, Folkesson R, Benedikz E, Sundstrom E: **Cloning and expression of the human N-methyl-D-aspartate receptor subunit NR3A.** *Neurosci Lett* 2002, **321:**177-181.

120. Nilsson A, Eriksson M, Muly EC, Akesson E, Samuelsson EB, Bogdanovic N, Benedikz E, Sundstrom E: **Analysis of NR3A receptor subunits in human native NMDA receptors.** *Brain Res* 2007, **1186:**102-112.

121. Tong G, Takahashi H, Tu S, Shin Y, Talantova M, Zago W, Xia P, Nie Z, Goetz T, Zhang D, et al: **Modulation of NMDA receptor properties and synaptic transmission by the NR3A subunit in mouse hippocampal and cerebrocortical neurons.** *J Neurophysiol* 2008, **99:**122-132.

122. Martinez-Turrillas R, Puerta E, Chowdhury D, Marco S, Watanabe M, Aguirre N, Perez-Otano I: **The NMDA receptor subunit GluN3A protects against 3-nitroproprionic-induced striatal lesions via inhibition of calpain activation.** *Neurobiol Dis* 2012, **48:**290-298.

123. Sattler R, Xiong Z, Lu WY, MacDonald JF, Tymianski M: **Distinct roles of synaptic and extrasynaptic NMDA receptors in excitotoxicity.** *J Neurosci* 2000, **20:**22-33.

124. Liu Y, Wong TP, Aarts M, Rooyakkers A, Liu L, Lai TW, Wu DC, Lu J, Tymianski M, Craig AM, Wang YT: **NMDA receptor subunits have differential roles in mediating excitotoxic neuronal death both in vitro and in vivo.** *J Neurosci* 2007, **27:**2846-2857.

125. Gladding CM, Raymond LA: **Mechanisms underlying NMDA receptor synaptic/extrasynaptic distribution and function.** *Mol Cell Neurosci* 2011, **48:**308-320.

126. Nakanishi N, Tu S, Shin Y, Cui J, Kurokawa T, Zhang D, Chen HS, Tong G, Lipton SA: **Neuroprotection by the NR3A subunit of the NMDA receptor.** *J Neurosci* 2009, **29:**5260-5265.

127. Zhong W, Wu A, Berglund K, Gu X, Jiang MQ, Talati J, Zhao J, Wei L, Yu SP: **Pathogenesis of sporadic Alzheimer's disease by deficiency of NMDA receptor subunit GluN3A.** *Alzheimers Dement* 2022, **18:**222-239.

128. Durst CD, Wiegert JS, Helassa N, Kerruth S, Coates C, Schulze C, Geeves MA, Torok K, Oertner TG: **High-speed imaging of glutamate release with genetically encoded sensors.** *Nat Protoc* 2019, **14:**1401-1424.

129. Szapiro G, Barbour B: **Parasynaptic signalling by fast neurotransmitters: the cerebellar cortex.** *Neuroscience* 2009, **162:**644-655.

130. Kessler JP: **Control of cleft glutamate concentration and glutamate spill-out by perisynaptic glia: uptake and diffusion barriers.** *PLoS One* 2013, **8:**e70791.

131. van der Zeyden M, Oldenziel WH, Rea K, Cremers TI, Westerink BH: **Microdialysis of GABA and glutamate: analysis, interpretation and comparison with microsensors.** *Pharmacol Biochem Behav* 2008, **90:**135-147.

132. Bezzi P, Gundersen V, Galbete JL, Seifert G, Steinhauser C, Pilati E, Volterra A: **Astrocytes contain a vesicular compartment that is competent for regulated exocytosis of glutamate.** *Nat Neurosci* 2004, **7:**613-620.

133. Hamilton NB, Attwell D: **Do astrocytes really exocytose neurotransmitters?** *Nat Rev Neurosci* 2010, **11:**227-238.

134. Fellin T, Pascual O, Gobbo S, Pozzan T, Haydon PG, Carmignoto G: **Neuronal synchrony mediated by astrocytic glutamate through activation of extrasynaptic NMDA receptors.** *Neuron* 2004, **43:**729-743.

135. Bergersen LH, Gundersen V: **Morphological evidence for vesicular glutamate release from astrocytes.** *Neuroscience* 2009, **158:**260-265.

136. Dash MB, Douglas CL, Vyazovskiy VV, Cirelli C, Tononi G: **Long-term homeostasis of extracellular glutamate in the rat cerebral cortex across sleep and waking states.** *J Neurosci* 2009, **29:**620-629.

137. Oldenziel WH, van der Zeyden M, Dijkstra G, Ghijsen WE, Karst H, Cremers TI, Westerink BH: **Monitoring extracellular glutamate in hippocampal slices with a microsensor.** *J Neurosci Methods* 2007, **160:**37-44.

138. Hascup KN, Hascup ER, Pomerleau F, Huettl P, Gerhardt GA: **Second-by-second measures of L-glutamate in the prefrontal cortex and striatum of freely moving mice.** *J Pharmacol Exp Ther* 2008, **324:**725-731.

139. Hascup ER, Hascup KN, Stephens M, Pomerleau F, Huettl P, Gratton A, Gerhardt GA: **Rapid microelectrode measurements and the origin and regulation of extracellular glutamate in rat prefrontal cortex.** *J Neurochem* 2010, **115:**1608-1620.

140. Herman MA, Jahr CE: **Extracellular glutamate concentration in hippocampal slice.** *J Neurosci* 2007, **27:**9736-9741.

141. Moldavski A, Behr J, Bading H, Bengtson CP: **A novel method using ambient glutamate for the electrophysiological quantification of extrasynaptic NMDA receptor function in acute brain slices.** *J Physiol* 2020, **598:**633-650.

142. Rosenberg PA, Amin S, Leitner M: **Glutamate uptake disguises neurotoxic potency of glutamate agonists in cerebral cortex in dissociated cell culture.** *J Neurosci* 1992, **12:**56-61.

143. Soria FN, Perez-Samartin A, Martin A, Gona KB, Llop J, Szczupak B, Chara JC, Matute C, Domercq M: **Extrasynaptic glutamate release through cystine/glutamate antiporter contributes to ischemic damage.** *J Clin Invest* 2014, **124:**3645-3655.

144. Moghaddam B: **Stress preferentially increases extraneuronal levels of excitatory amino acids in the prefrontal cortex: comparison to hippocampus and basal ganglia.** *J Neurochem* 1993, **60:**1650-1657.

145. Lerma J, Herranz AS, Herreras O, Abraira V, Martin del Rio R: **In vivo determination of extracellular concentration of amino acids in the rat hippocampus. A method based on brain dialysis and computerized analysis.** *Brain Res* 1986, **384:**145-155.

146. Dirnagl U, Iadecola C, Moskowitz MA: **Pathobiology of ischaemic stroke: an integrated view.** *Trends Neurosci* 1999, **22:**391-397.

147. Pal B: **Involvement of extrasynaptic glutamate in physiological and pathophysiological changes of neuronal excitability.** *Cell Mol Life Sci* 2018, **75:**2917-2949.

148. Verma M, Lizama BN, Chu CT: **Excitotoxicity, calcium and mitochondria: a triad in synaptic neurodegeneration.** *Transl Neurodegener* 2022, **11:**3.

149. Brymer KJ, Barnes JR, Parsons MP: **Entering a new era of quantifying glutamate clearance in health and disease.** *J Neurosci Res* 2021, **99:**1598-1617.

150. Arias C, Arrieta I, Tapia R: **beta-Amyloid peptide fragment 25-35 potentiates the calcium-dependent release of excitatory amino acids from depolarized hippocampal slices.** *J Neurosci Res* 1995, **41:**561-566.

151. Gross V, Weiss E, Northemann W, Scheurlen M, Heinrich PC: **Comparative studies of the effects of galactosamine and actinomycin D on nuclear ribonucleoprotein particles from rat liver.** *Exp Cell Res* 1977, **109:**331-339.

152. Fernandez-Tome P, Brera B, Arevalo MA, de Ceballos ML: **Beta-amyloid25-35 inhibits glutamate uptake in cultured neurons and astrocytes: modulation of uptake as a survival mechanism.** *Neurobiol Dis* 2004, **15:**580-589.

153. Talantova M, Sanz-Blasco S, Zhang X, Xia P, Akhtar MW, Okamoto S, Dziewczapolski G, Nakamura T, Cao G, Pratt AE, et al: **Abeta induces astrocytic glutamate release, extrasynaptic NMDA receptor activation, and synaptic loss.** *Proc Natl Acad Sci U S A* 2013, **110:**E2518-2527.

154. Wang R, Reddy PH: **Role of Glutamate and NMDA Receptors in Alzheimer's Disease.** *J Alzheimers Dis* 2017, **57:**1041-1048.

155. Zhou Y, Song WM, Andhey PS, Swain A, Levy T, Miller KR, Poliani PL, Cominelli M, Grover S, Gilfillan S, et al: **Human and mouse single-nucleus transcriptomics reveal TREM2-dependent and TREM2-independent cellular responses in Alzheimer's disease.** *Nat Med* 2020, **26:**131-142.

156. Johnson ECB, Dammer EB, Duong DM, Ping L, Zhou M, Yin L, Higginbotham LA, Guajardo A, White B, Troncoso JC, et al: **Large-scale proteomic analysis of Alzheimer's disease brain and cerebrospinal fluid reveals early changes in energy metabolism associated with microglia and astrocyte activation.** *Nat Med* 2020, **26:**769-780.

157. Kirvell SL, Esiri M, Francis PT: **Down-regulation of vesicular glutamate transporters precedes cell loss and pathology in Alzheimer's disease.** *J Neurochem* 2006, **98:**939-950.

158. Scott HA, Gebhardt FM, Mitrovic AD, Vandenberg RJ, Dodd PR: **Glutamate transporter variants reduce glutamate uptake in Alzheimer's disease.** *Neurobiol Aging* 2011, **32:**553 e551-511.

159. Ludhiadch A, Sharma R, Muriki A, Munshi A: **Role of Calcium Homeostasis in Ischemic Stroke: A Review.** *CNS Neurol Disord Drug Targets* 2022, **21:**52-61.

160. Choi DW: **Calcium-mediated neurotoxicity: relationship to specific channel types and role in ischemic damage.** *Trends Neurosci* 1988, **11:**465-469.

161. Jiang MQ, Zhao YY, Cao W, Wei ZZ, Gu X, Wei L, Yu SP: **Long-term survival and regeneration of neuronal and vasculature cells inside the core region after ischemic stroke in adult mice.** *Brain Pathol* 2017, **27:**480-498.

162. Wei L, Ying DJ, Cui L, Langsdorf J, Yu S: **Necrosis, apoptosis and hybrid death in the cortex and thalamus after barrel cortex ischemia in rats.** *Brain Res* 2004, **1022:**54-61.

163. Bordji K, Becerril-Ortega J, Nicole O, Buisson A: **Activation of extrasynaptic, but not synaptic, NMDA receptors modifies amyloid precursor protein expression pattern and increases amyloid-ss production.** *J Neurosci* 2010, **30:**15927-15942.

164. Amadoro G, Ciotti MT, Costanzi M, Cestari V, Calissano P, Canu N: **NMDA receptor mediates tau-induced neurotoxicity by calpain and ERK/MAPK activation.** *Proc Natl Acad Sci U S A* 2006, **103:**2892-2897.

165. Milnerwood AJ, Gladding CM, Pouladi MA, Kaufman AM, Hines RM, Boyd JD, Ko RW, Vasuta OC, Graham RK, Hayden MR, et al: **Early increase in extrasynaptic NMDA receptor signaling and expression contributes to phenotype onset in Huntington's disease mice.** *Neuron* 2010, **65:**178-190.

166. Butterfield DA, Pocernich CB: **The glutamatergic system and Alzheimer's disease: therapeutic implications.** *CNS Drugs* 2003, **17:**641-652.

167. Yin J, VanDongen AM: **Enhanced Neuronal Activity and Asynchronous Calcium Transients Revealed in a 3D Organoid Model of Alzheimer's Disease.** *ACS Biomater Sci Eng* 2021, **7:**254-264.

168. Stutzmann GE: **The pathogenesis of Alzheimers disease is it a lifelong "calciumopathy"?** *Neuroscientist* 2007, **13:**546-559.

169. Busche MA, Chen X, Henning HA, Reichwald J, Staufenbiel M, Sakmann B, Konnerth A: **Critical role of soluble amyloid-beta for early hippocampal hyperactivity in a mouse model of Alzheimer's disease.** *Proc Natl Acad Sci U S A* 2012, **109:**8740-8745.

170. Dickstein DL, Kabaso D, Rocher AB, Luebke JI, Wearne SL, Hof PR: **Changes in the structural complexity of the aged brain.** *Aging Cell* 2007, **6:**275-284.

171. Lin CH, Huang YJ, Lin CJ, Lane HY, Tsai GE: **NMDA neurotransmission dysfunction in mild cognitive impairment and Alzheimer's disease.** *Curr Pharm Des* 2014, **20:**5169-5179.

172. Angulo SL, Henzi T, Neymotin SA, Suarez MD, Lytton WW, Schwaller B, Moreno H: **Amyloid pathology-produced unexpected modifications of calcium homeostasis in hippocampal subicular dendrites.** *Alzheimers Dement* 2020, **16:**251-261.

173. Sosulina L, Mittag M, Geis HR, Hoffmann K, Klyubin I, Qi Y, Steffen J, Friedrichs D, Henneberg N, Fuhrmann F, et al: **Hippocampal hyperactivity in a rat model of Alzheimer's disease.** *J Neurochem* 2021, **157:**2128-2144.

174. Wang Y, Shi Z, Zhang Y, Yan J, Yu W, Chen L: **Oligomer beta-amyloid Induces Hyperactivation of Ras to Impede NMDA Receptor-Dependent Long-Term Potentiation in Hippocampal CA1 of Mice.** *Front Pharmacol* 2020, **11:**595360.

175. Hector A, Brouillette J: **Hyperactivity Induced by Soluble Amyloid-beta Oligomers in the Early Stages of Alzheimer's Disease.** *Front Mol Neurosci* 2020, **13:**600084.

176. Le Meur K, Galante M, Angulo MC, Audinat E: **Tonic activation of NMDA receptors by ambient glutamate of non-synaptic origin in the rat hippocampus.** *J Physiol* 2007, **580:**373-383.

177. Sah P, Hestrin S, Nicoll RA: **Tonic activation of NMDA receptors by ambient glutamate enhances excitability of neurons.** *Science* 1989, **246:**815-818.

178. Angulo MC, Kozlov AS, Charpak S, Audinat E: **Glutamate released from glial cells synchronizes neuronal activity in the hippocampus.** *J Neurosci* 2004, **24:**6920-6927.

179. Brassai A, Suvanjeiev RG, Ban EG, Lakatos M: **Role of synaptic and nonsynaptic glutamate receptors in ischaemia induced neurotoxicity.** *Brain Res Bull* 2015, **112:**1-6.

180. Vizi ES, Kisfali M, Lorincz T: **Role of nonsynaptic GluN2B-containing NMDA receptors in excitotoxicity: evidence that fluoxetine selectively inhibits these receptors and may have neuroprotective effects.** *Brain Res Bull* 2013, **93:**32-38.

181. Somjen GG: **Mechanisms of spreading depression and hypoxic spreading depression-like depolarization.** *Physiol Rev* 2001, **81:**1065-1096.

182. Abramov AY, Duchen MR: **Mechanisms underlying the loss of mitochondrial membrane potential in glutamate excitotoxicity.** *Biochim Biophys Acta* 2008, **1777:**953-964.

183. Xia P, Chen HS, Zhang D, Lipton SA: **Memantine preferentially blocks extrasynaptic over synaptic NMDA receptor currents in hippocampal autapses.** *J Neurosci* 2010, **30:**11246-11250.

184. Hardingham GE, Bading H: **Synaptic versus extrasynaptic NMDA receptor signalling: implications for neurodegenerative disorders.** *Nat Rev Neurosci* 2010, **11:**682-696.

185. Mira RG, Cerpa W: **Building a Bridge Between NMDAR-Mediated Excitotoxicity and Mitochondrial Dysfunction in Chronic and Acute Diseases.** *Cell Mol Neurobiol* 2021, **41:**1413-1430.

186. Lai TW, Shyu WC, Wang YT: **Stroke intervention pathways: NMDA receptors and beyond.** *Trends Mol Med* 2011, **17:**266-275.

187. Martel MA, Ryan TJ, Bell KF, Fowler JH, McMahon A, Al-Mubarak B, Komiyama NH, Horsburgh K, Kind PC, Grant SG, et al: **The subtype of GluN2 C-terminal domain determines the response to excitotoxic insults.** *Neuron* 2012, **74:**543-556.

188. Karpova A, Mikhaylova M, Bera S, Bar J, Reddy PP, Behnisch T, Rankovic V, Spilker C, Bethge P, Sahin J, et al: **Encoding and transducing the synaptic or extrasynaptic origin of NMDA receptor signals to the nucleus.** *Cell* 2013, **152:**1119-1133.

189. Hardingham GE, Arnold FJ, Bading H: **Nuclear calcium signaling controls CREB-mediated gene expression triggered by synaptic activity.** *Nat Neurosci* 2001, **4:**261-267.

190. Ivanov A, Pellegrino C, Rama S, Dumalska I, Salyha Y, Ben-Ari Y, Medina I: **Opposing role of synaptic and extrasynaptic NMDA receptors in regulation of the extracellular signal-regulated kinases (ERK) activity in cultured rat hippocampal neurons.** *J Physiol* 2006, **572:**789-798.

191. Dick O, Bading H: **Synaptic activity and nuclear calcium signaling protect hippocampal neurons from death signal-associated nuclear translocation of FoxO3a induced by extrasynaptic N-methyl-D-aspartate receptors.** *J Biol Chem* 2010, **285:**19354-19361.

192. Nakazawa H, Murphy TH: **Activation of nuclear calcium dynamics by synaptic stimulation in cultured cortical neurons.** *J Neurochem* 1999, **73:**1075-1083.

193. Zheng S, Eacker SM, Hong SJ, Gronostajski RM, Dawson TM, Dawson VL: **NMDA-induced neuronal survival is mediated through nuclear factor I-A in mice.** *J Clin Invest* 2010, **120:**2446-2456.

194. Grochowska KM, Bar J, Gomes GM, Kreutz MR, Karpova A: **Jacob, a Synapto-Nuclear Protein Messenger Linking N-methyl-D-aspartate Receptor Activation to Nuclear Gene Expression.** *Front Synaptic Neurosci* 2021, **13:**787494.

195. Dieterich DC, Karpova A, Mikhaylova M, Zdobnova I, Konig I, Landwehr M, Kreutz M, Smalla KH, Richter K, Landgraf P, et al: **Caldendrin-Jacob: a protein liaison that couples NMDA receptor signalling to the nucleus.** *PLoS Biol* 2008, **6:**e34.

196. Crivellaro G, Tottene A, Vitale M, Melone M, Casari G, Conti F, Santello M, Pietrobon D: **Specific activation of GluN1-N2B NMDA receptors underlies facilitation of cortical spreading depression in a genetic mouse model of migraine with reduced astrocytic glutamate clearance.** *Neurobiol Dis* 2021, **156:**105419.

197. Wang H, Yu SW, Koh DW, Lew J, Coombs C, Bowers W, Federoff HJ, Poirier GG, Dawson TM, Dawson VL: **Apoptosis-inducing factor substitutes for caspase executioners in NMDA-triggered excitotoxic neuronal death.** *J Neurosci* 2004, **24:**10963-10973.

198. Bahia PK, Pugh V, Hoyland K, Hensley V, Rattray M, Williams RJ: **Neuroprotective effects of phenolic antioxidant tBHQ associate with inhibition of FoxO3a nuclear translocation and activity.** *J Neurochem* 2012, **123:**182-191.

199. Xu J, Kurup P, Zhang Y, Goebel-Goody SM, Wu PH, Hawasli AH, Baum ML, Bibb JA, Lombroso PJ: **Extrasynaptic NMDA receptors couple preferentially to excitotoxicity via calpain-mediated cleavage of STEP.** *J Neurosci* 2009, **29:**9330-9343.

200. Hardingham GE, Bading H: **Coupling of extrasynaptic NMDA receptors to a CREB shut-off pathway is developmentally regulated.** *Biochim Biophys Acta* 2002, **1600:**148-153.

201. Mikhaylova M, Karpova A, Bar J, Bethge P, YuanXiang P, Chen Y, Zuschratter W, Behnisch T, Kreutz MR: **Cellular distribution of the NMDA-receptor activated synapto-nuclear messenger Jacob in the rat brain.** *Brain Struct Funct* 2014, **219:**843-860.

202. Ferreira IL, Ferreiro E, Schmidt J, Cardoso JM, Pereira CM, Carvalho AL, Oliveira CR, Rego AC: **Abeta and NMDAR activation cause mitochondrial dysfunction involving ER calcium release.** *Neurobiol Aging* 2015, **36:**680-692.

203. Picard M, McEwen BS: **Mitochondria impact brain function and cognition.** *Proc Natl Acad Sci U S A* 2014, **111:**7-8.

204. Wang Y, Wu L, Li J, Fang D, Zhong C, Chen JX, Yan SS: **Synergistic exacerbation of mitochondrial and synaptic dysfunction and resultant learning and memory deficit in a mouse model of diabetic Alzheimer's disease.** *J Alzheimers Dis* 2015, **43:**451-463.

205. Papadia S, Soriano FX, Leveille F, Martel MA, Dakin KA, Hansen HH, Kaindl A, Sifringer M, Fowler J, Stefovska V, et al: **Synaptic NMDA receptor activity boosts intrinsic antioxidant defenses.** *Nat Neurosci* 2008, **11:**476-487.

206. Choi DW: **Glutamate neurotoxicity and diseases of the nervous system.** *Neuron* 1988, **1:**623-634.

207. Yu SP, Yeh C, Strasser U, Tian M, Choi DW: **NMDA receptor-mediated K+ efflux and neuronal apoptosis.** *Science* 1999, **284:**336-339.

208. Song M, Yu SP: **Ionic regulation of cell volume changes and cell death after ischemic stroke.** *Transl Stroke Res* 2014, **5:**17-27.

209. Camacho A, Massieu L: **Role of glutamate transporters in the clearance and release of glutamate during ischemia and its relation to neuronal death.** *Arch Med Res* 2006, **37:**11-18.

210. Rossi DJ, Oshima T, Attwell D: **Glutamate release in severe brain ischaemia is mainly by reversed uptake.** *Nature* 2000, **403:**316-321.

211. Rajdev S, Reynolds IJ: **Calcium green-5N, a novel fluorescent probe for monitoring high intracellular free Ca2+ concentrations associated with glutamate excitotoxicity in cultured rat brain neurons.** *Neurosci Lett* 1993, **162:**149-152.

212. Kiedrowski L: **N-methyl-D-aspartate excitotoxicity: relationships among plasma membrane potential, Na(+)/Ca(2+) exchange, mitochondrial Ca(2+) overload, and cytoplasmic concentrations of Ca(2+), H(+), and K(+).** *Mol Pharmacol* 1999, **56:**619-632.

213. Choi DW, Koh JY, Peters S: **Pharmacology of glutamate neurotoxicity in cortical cell culture: attenuation by NMDA antagonists.** *J Neurosci* 1988, **8:**185-196.

214. Tymianski M, Charlton MP, Carlen PL, Tator CH: **Source specificity of early calcium neurotoxicity in cultured embryonic spinal neurons.** *J Neurosci* 1993, **13:**2085-2104.

215. Wei L, Han BH, Li Y, Keogh CL, Holtzman DM, Yu SP: **Cell death mechanism and protective effect of erythropoietin after focal ischemia in the whisker-barrel cortex of neonatal rats.** *J Pharmacol Exp Ther* 2006, **317:**109-116.

216. Xiao AY, Wei L, Xia S, Rothman S, Yu SP: **Ionic mechanism of ouabain-induced concurrent apoptosis and necrosis in individual cultured cortical neurons.** *J Neurosci* 2002, **22:**1350-1362.

217. Fayaz SM, Suvanish Kumar VS, Rajanikant GK: **Necroptosis: who knew there were so many interesting ways to die?** *CNS Neurol Disord Drug Targets* 2014, **13:**42-51.

218. Binvignat O, Olloquequi J: **Excitotoxicity as a Target Against Neurodegenerative Processes.** *Curr Pharm Des* 2020, **26:**1251-1262.

219. Khachaturian ZS: **Calcium hypothesis of Alzheimer's disease and brain aging.** *Ann N Y Acad Sci* 1994, **747:**1-11.

220. Marx J: **Alzheimer's disease. Fresh evidence points to an old suspect: calcium.** *Science* 2007, **318:**384-385.

221. Lipton SA, Rosenberg PA: **Excitatory amino acids as a final common pathway for neurologic disorders.** *N Engl J Med* 1994, **330:**613-622.

222. Alzheimer's Association Calcium Hypothesis W: **Calcium Hypothesis of Alzheimer's disease and brain aging: A framework for integrating new evidence into a comprehensive theory of pathogenesis.** *Alzheimers Dement* 2017, **13:**178-182 e117.

223. Bao Y, Yang X, Fu Y, Li Z, Gong R, Lu W: **NMDAR-dependent somatic potentiation of synaptic inputs is correlated with beta amyloid-mediated neuronal hyperactivity.** *Transl Neurodegener* 2021, **10:**34.

224. Ong WY, Tanaka K, Dawe GS, Ittner LM, Farooqui AA: **Slow excitotoxicity in Alzheimer's disease.** *J Alzheimers Dis* 2013, **35:**643-668.

225. Jiang M, Fidler, J., Berglund, K., Gu, X., Wu, A., Estaba, T., Patel, J., Wei, L., Yu, S.P.: **Delayed and brain region specific deletion of the NMDA receptor subunit GluN3A causes AD-like functional and behavioral changes in mice.** *Society for Neuroscience 2022 Abstrct* 2022, **426:**10603.

226. Simon F, Varela D, Cabello-Verrugio C: **Oxidative stress-modulated TRPM ion channels in cell dysfunction and pathological conditions in humans.** *Cell Signal* 2013, **25:**1614-1624.

227. Vennekens R, Nilius B: **Insights into TRPM4 function, regulation and physiological role.** *Handb Exp Pharmacol* 2007**:**269-285.

228. Wang L, Fu TM, Zhou Y, Xia S, Greka A, Wu H: **Structures and gating mechanism of human TRPM2.** *Science* 2018, **362**.

229. Yan J, Bengtson CP, Buchthal B, Hagenston AM, Bading H: **Coupling of NMDA receptors and TRPM4 guides discovery of unconventional neuroprotectants.** *Science* 2020, **370**.

230. Zong P, Feng J, Yue Z, Li Y, Wu G, Sun B, He Y, Miller B, Yu AS, Su Z, et al: **Functional coupling of TRPM2 and extrasynaptic NMDARs exacerbates excitotoxicity in ischemic brain injury.** *Neuron* 2022, **110:**1944-1958 e1948.

231. Yildizhan K, Naziroglu M: **NMDA Receptor Activation Stimulates Hypoxia-Induced TRPM2 Channel Activation, Mitochondrial Oxidative Stress, and Apoptosis in Neuronal Cell Line: Modular Role of Memantine.** *Brain Res* 2023, **1803:**148232.

232. Jiang LH, Li X, Syed Mortadza SA, Lovatt M, Yang W: **The TRPM2 channel nexus from oxidative damage to Alzheimer's pathologies: An emerging novel intervention target for age-related dementia.** *Ageing Res Rev* 2018, **47:**67-79.

233. Ferreira IL, Bajouco LM, Mota SI, Auberson YP, Oliveira CR, Rego AC: **Amyloid beta peptide 1-42 disturbs intracellular calcium homeostasis through activation of GluN2B-containing N-methyl-d-aspartate receptors in cortical cultures.** *Cell Calcium* 2012, **51:**95-106.

234. Sinnen BL, Bowen AB, Gibson ES, Kennedy MJ: **Local and Use-Dependent Effects of beta-Amyloid Oligomers on NMDA Receptor Function Revealed by Optical Quantal Analysis.** *J Neurosci* 2016, **36:**11532-11543.

235. Taniguchi K, Yamamoto F, Amamo A, Tamaoka A, Sanjo N, Yokota T, Kametani F, Araki W: **Amyloid-beta oligomers interact with NMDA receptors containing GluN2B subunits and metabotropic glutamate receptor 1 in primary cortical neurons: Relevance to the synapse pathology of Alzheimer's disease.** *Neurosci Res* 2022, **180:**90-98.

236. Olajide OJ, Chapman CA: **Amyloid-beta (1-42) peptide induces rapid NMDA receptor-dependent alterations at glutamatergic synapses in the entorhinal cortex.** *Neurobiol Aging* 2021, **105:**296-309.

237. De Felice FG, Velasco PT, Lambert MP, Viola K, Fernandez SJ, Ferreira ST, Klein WL: **Abeta oligomers induce neuronal oxidative stress through an N-methyl-D-aspartate receptor-dependent mechanism that is blocked by the Alzheimer drug memantine.** *J Biol Chem* 2007, **282:**11590-11601.

238. Lacor PN, Buniel MC, Furlow PW, Clemente AS, Velasco PT, Wood M, Viola KL, Klein WL: **Abeta oligomer-induced aberrations in synapse composition, shape, and density provide a molecular basis for loss of connectivity in Alzheimer's disease.** *J Neurosci* 2007, **27:**796-807.

239. Venkitaramani DV, Chin J, Netzer WJ, Gouras GK, Lesne S, Malinow R, Lombroso PJ: **Beta-amyloid modulation of synaptic transmission and plasticity.** *J Neurosci* 2007, **27:**11832-11837.

240. Malinow R: **New developments on the role of NMDA receptors in Alzheimer's disease.** *Curr Opin Neurobiol* 2012, **22:**559-563.

241. Liu J, Chang L, Song Y, Li H, Wu Y: **The Role of NMDA Receptors in Alzheimer's Disease.** *Front Neurosci* 2019, **13:**43.

242. Cline EN, Bicca MA, Viola KL, Klein WL: **The Amyloid-beta Oligomer Hypothesis: Beginning of the Third Decade.** *J Alzheimers Dis* 2018, **64:**S567-S610.

243. Parameshwaran K, Dhanasekaran M, Suppiramaniam V: **Amyloid beta peptides and glutamatergic synaptic dysregulation.** *Exp Neurol* 2008, **210:**7-13.

244. Texido L, Martin-Satue M, Alberdi E, Solsona C, Matute C: **Amyloid beta peptide oligomers directly activate NMDA receptors.** *Cell Calcium* 2011, **49:**184-190.

245. Snyder EM, Nong Y, Almeida CG, Paul S, Moran T, Choi EY, Nairn AC, Salter MW, Lombroso PJ, Gouras GK, Greengard P: **Regulation of NMDA receptor trafficking by amyloid-beta.** *Nat Neurosci* 2005, **8:**1051-1058.

246. Noda M, Nakanishi H, Akaike N: **Glutamate release from microglia via glutamate transporter is enhanced by amyloid-beta peptide.** *Neuroscience* 1999, **92:**1465-1474.

247. Rudy CC, Hunsberger HC, Weitzner DS, Reed MN: **The role of the tripartite glutamatergic synapse in the pathophysiology of Alzheimer's disease.** *Aging Dis* 2015, **6:**131-148.

248. Zhang Y, Li P, Feng J, Wu M: **Dysfunction of NMDA receptors in Alzheimer's disease.** *Neurol Sci* 2016, **37:**1039-1047.

249. Pinheiro L, Faustino C: **Therapeutic Strategies Targeting Amyloid-beta in Alzheimer's Disease.** *Curr Alzheimer Res* 2019, **16:**418-452.

250. Sun XY, Tuo QZ, Liuyang ZY, Xie AJ, Feng XL, Yan X, Qiu M, Li S, Wang XL, Cao FY, et al: **Extrasynaptic NMDA receptor-induced tau overexpression mediates neuronal death through suppressing survival signaling ERK phosphorylation.** *Cell Death Dis* 2016, **7:**e2449.

251. Miao Y, Dong LD, Chen J, Hu XC, Yang XL, Wang Z: **Involvement of calpain/p35-p25/Cdk5/NMDAR signaling pathway in glutamate-induced neurotoxicity in cultured rat retinal neurons.** *PLoS One* 2012, **7:**e42318.

252. Hoshi M, Sato M, Matsumoto S, Noguchi A, Yasutake K, Yoshida N, Sato K: **Spherical aggregates of beta-amyloid (amylospheroid) show high neurotoxicity and activate tau protein kinase I/glycogen synthase kinase-3beta.** *Proc Natl Acad Sci U S A* 2003, **100:**6370-6375.

253. Tackenberg C, Grinschgl S, Trutzel A, Santuccione AC, Frey MC, Konietzko U, Grimm J, Brandt R, Nitsch RM: **NMDA receptor subunit composition determines beta-amyloid-induced neurodegeneration and synaptic loss.** *Cell Death Dis* 2013, **4:**e608.

254. Decker H, Lo KY, Unger SM, Ferreira ST, Silverman MA: **Amyloid-beta peptide oligomers disrupt axonal transport through an NMDA receptor-dependent mechanism that is mediated by glycogen synthase kinase 3beta in primary cultured hippocampal neurons.** *J Neurosci* 2010, **30:**9166-9171.

255. Pallas-Bazarra N, Draffin J, Cuadros R, Antonio Esteban J, Avila J: **Tau is required for the function of extrasynaptic NMDA receptors.** *Sci Rep* 2019, **9:**9116.

256. Lesne S, Ali C, Gabriel C, Croci N, MacKenzie ET, Glabe CG, Plotkine M, Marchand-Verrecchia C, Vivien D, Buisson A: **NMDA receptor activation inhibits alpha-secretase and promotes neuronal amyloid-beta production.** *J Neurosci* 2005, **25:**9367-9377.

257. Cirrito JR, Yamada KA, Finn MB, Sloviter RS, Bales KR, May PC, Schoepp DD, Paul SM, Mennerick S, Holtzman DM: **Synaptic activity regulates interstitial fluid amyloid-beta levels in vivo.** *Neuron* 2005, **48:**913-922.

258. Ronicke R, Mikhaylova M, Ronicke S, Meinhardt J, Schroder UH, Fandrich M, Reiser G, Kreutz MR, Reymann KG: **Early neuronal dysfunction by amyloid beta oligomers depends on activation of NR2B-containing NMDA receptors.** *Neurobiol Aging* 2011, **32:**2219-2228.

259. Blurton-Jones M, Kitazawa M, Martinez-Coria H, Castello NA, Muller FJ, Loring JF, Yamasaki TR, Poon WW, Green KN, LaFerla FM: **Neural stem cells improve cognition via BDNF in a transgenic model of Alzheimer disease.** *Proc Natl Acad Sci U S A* 2009, **106:**13594-13599.

260. Fernandez-Garcia S, Sancho-Balsells A, Longueville S, Herve D, Gruart A, Delgado-Garcia JM, Alberch J, Giralt A: **Astrocytic BDNF and TrkB regulate severity and neuronal activity in mouse models of temporal lobe epilepsy.** *Cell Death Dis* 2020, **11:**411.

261. Caccamo A, Maldonado MA, Bokov AF, Majumder S, Oddo S: **CBP gene transfer increases BDNF levels and ameliorates learning and memory deficits in a mouse model of Alzheimer's disease.** *Proc Natl Acad Sci U S A* 2010, **107:**22687-22692.

262. Hoey SE, Williams RJ, Perkinton MS: **Synaptic NMDA receptor activation stimulates alpha-secretase amyloid precursor protein processing and inhibits amyloid-beta production.** *J Neurosci* 2009, **29:**4442-4460.

263. Hetman M, Kharebava G: **Survival signaling pathways activated by NMDA receptors.** *Curr Top Med Chem* 2006, **6:**787-799.

264. Javitt DC, Zukin SR: **Recent advances in the phencyclidine model of schizophrenia.** *Am J Psychiatry* 1991, **148:**1301-1308.

265. Krystal JH, Karper LP, Seibyl JP, Freeman GK, Delaney R, Bremner JD, Heninger GR, Bowers MB, Jr., Charney DS: **Subanesthetic effects of the noncompetitive NMDA antagonist, ketamine, in humans. Psychotomimetic, perceptual, cognitive, and neuroendocrine responses.** *Arch Gen Psychiatry* 1994, **51:**199-214.

266. Lahti AC, Holcomb HH, Medoff DR, Tamminga CA: **Ketamine activates psychosis and alters limbic blood flow in schizophrenia.** *Neuroreport* 1995, **6:**869-872.

267. Parsons CG, Danysz W, Quack G: **Memantine is a clinically well tolerated N-methyl-D-aspartate (NMDA) receptor antagonist--a review of preclinical data.** *Neuropharmacology* 1999, **38:**735-767.

268. Lipton SA: **Paradigm shift in neuroprotection by NMDA receptor blockade: memantine and beyond.** *Nat Rev Drug Discov* 2006, **5:**160-170.

269. Wu YN, Johnson SW: **Memantine selectively blocks extrasynaptic NMDA receptors in rat substantia nigra dopamine neurons.** *Brain Res* 2015, **1603:**1-7.

270. Aluclu MU, Arslan S, Acar A, Guzel A, Bahceci S, Yaldiz M: **Evaluation of effects of memantine on cerebral ischemia in rats.** *Neurosciences (Riyadh)* 2008, **13:**113-116, PMID: 21063302.

271. Seif el Nasr M, Peruche B, Rossberg C, Mennel HD, Krieglstein J: **Neuroprotective effect of memantine demonstrated in vivo and in vitro.** *Eur J Pharmacol* 1990, **185:**19-24.

272. Kilic U, Yilmaz B, Reiter RJ, Yuksel A, Kilic E: **Effects of memantine and melatonin on signal transduction pathways vascular leakage and brain injury after focal cerebral ischemia in mice.** *Neuroscience* 2013, **237:**268-276.

273. Chen B, Wang G, Li W, Liu W, Lin R, Tao J, Jiang M, Chen L, Wang Y: **Memantine attenuates cell apoptosis by suppressing the calpain-caspase-3 pathway in an experimental model of ischemic stroke.** *Exp Cell Res* 2017, **351:**163-172.

274. Culmsee C, Junker V, Kremers W, Thal S, Plesnila N, Krieglstein J: **Combination therapy in ischemic stroke: synergistic neuroprotective effects of memantine and clenbuterol.** *Stroke* 2004, **35:**1197-1202.

275. Trotman M, Vermehren P, Gibson CL, Fern R: **The dichotomy of memantine treatment for ischemic stroke: dose-dependent protective and detrimental effects.** *J Cereb Blood Flow Metab* 2015, **35:**230-239.

276. Lapchak PA: **Memantine, an uncompetitive low affinity NMDA open-channel antagonist improves clinical rating scores in a multiple infarct embolic stroke model in rabbits.** *Brain Res* 2006, **1088:**141-147.

277. Beconi MG, Howland D, Park L, Lyons K, Giuliano J, Dominguez C, Munoz-Sanjuan I, Pacifici R: **Pharmacokinetics of memantine in rats and mice.** *PLoS Curr* 2011, **3:**RRN1291.

278. Martinez-Coria H, Green KN, Billings LM, Kitazawa M, Albrecht M, Rammes G, Parsons CG, Gupta S, Banerjee P, LaFerla FM: **Memantine improves cognition and reduces Alzheimer's-like neuropathology in transgenic mice.** *Am J Pathol* 2010, **176:**870-880.

279. Lopez-Valdes HE, Clarkson AN, Ao Y, Charles AC, Carmichael ST, Sofroniew MV, Brennan KC: **Memantine enhances recovery from stroke.** *Stroke* 2014, **45:**2093-2100.

280. Stazi M, Wirths O: **Chronic Memantine Treatment Ameliorates Behavioral Deficits, Neuron Loss, and Impaired Neurogenesis in a Model of Alzheimer's Disease.** *Mol Neurobiol* 2021, **58:**204-216.

281. Atri A: **The Alzheimer's Disease Clinical Spectrum: Diagnosis and Management.** *Med Clin North Am* 2019, **103:**263-293.

282. Jin BR, Liu HY: **Comparative efficacy and safety of cognitive enhancers for treating vascular cognitive impairment: systematic review and Bayesian network meta-analysis.** *Neural Regen Res* 2019, **14:**805-816.

283. Tricco AC, Ashoor HM, Soobiah C, Rios P, Veroniki AA, Hamid JS, Ivory JD, Khan PA, Yazdi F, Ghassemi M, et al: **Comparative Effectiveness and Safety of Cognitive Enhancers for Treating Alzheimer's Disease: Systematic Review and Network Metaanalysis.** *J Am Geriatr Soc* 2018, **66:**170-178.

284. Farlow MR, Graham SM, Alva G: **Memantine for the treatment of Alzheimer's disease: tolerability and safety data from clinical trials.** *Drug Saf* 2008, **31:**577-585.

285. Wilcock G, Mobius HJ, Stoffler A, group MMM: **A double-blind, placebo-controlled multicentre study of memantine in mild to moderate vascular dementia (MMM500).** *Int Clin Psychopharmacol* 2002, **17:**297-305.

286. Chen HS, Pellegrini JW, Aggarwal SK, Lei SZ, Warach S, Jensen FE, Lipton SA: **Open-channel block of N-methyl-D-aspartate (NMDA) responses by memantine: therapeutic advantage against NMDA receptor-mediated neurotoxicity.** *J Neurosci* 1992, **12:**4427-4436.

287. Kuns B, Rosani A, D. V: **Mematine.** In *StatPearls [Internet].* *Volume* Jan-. FL: StatPearls Publishing; 2022: PMID: 29763201

288. Ditzler K: **Efficacy and tolerability of memantine in patients with dementia syndrome. A double-blind, placebo controlled trial.** *Arzneimittelforschung* 1991, **41:**773-780, PMID: 1781796.

289. Matsunaga S, Kishi T, Iwata N: **Memantine monotherapy for Alzheimer's disease: a systematic review and meta-analysis.** *PLoS One* 2015, **10:**e0123289.

290. Folch J, Busquets O, Ettcheto M, Sanchez-Lopez E, Castro-Torres RD, Verdaguer E, Garcia ML, Olloquequi J, Casadesus G, Beas-Zarate C, et al: **Memantine for the Treatment of Dementia: A Review on its Current and Future Applications.** *J Alzheimers Dis* 2018, **62:**1223-1240.

291. Souchet B, Audrain M, Alves S, Fol R, Tada S, Orefice NS, Potier B, Dutar P, Billard JM, Cartier N, Braudeau J: **Evaluation of Memantine in AAV-AD Rat: A Model of Late-Onset Alzheimer's Disease Predementia.** *J Prev Alzheimers Dis* 2022, **9:**338-347, PMID: 35543008.

292. Post SG, Whitehouse PJ, Binstock RH, Bird TD, Eckert SK, Farrer LA, Fleck LM, Gaines AD, Juengst ET, Karlinsky H, et al: **The clinical introduction of genetic testing for Alzheimer disease. An ethical perspective.** *JAMA* 1997, **277:**832-836.

293. Kamboh MI: **Genomics and Functional Genomics of Alzheimer's Disease.** *Neurotherapeutics* 2022, **19:**152-172.

294. Hardy J, Escott-Price V: **Genes, pathways and risk prediction in Alzheimer's disease.** *Hum Mol Genet* 2019, **28:**R235-R240.

295. Malik R, Chauhan G, Traylor M, Sargurupremraj M, Okada Y, Mishra A, Rutten-Jacobs L, Giese AK, van der Laan SW, Gretarsdottir S, et al: **Multiancestry genome-wide association study of 520,000 subjects identifies 32 loci associated with stroke and stroke subtypes.** *Nat Genet* 2018, **50:**524-537.

296. Schmidt R, Ropele S, Pendl B, Ofner P, Enzinger C, Schmidt H, Berghold A, Windisch M, Kolassa H, Fazekas F: **Longitudinal multimodal imaging in mild to moderate Alzheimer disease: a pilot study with memantine.** *J Neurol Neurosurg Psychiatry* 2008, **79:**1312-1317.

297. Mobius HJ, Stoffler A: **Memantine in vascular dementia.** *Int Psychogeriatr* 2003, **15 Suppl 1:**207-213.

298. Zhang N, Wei C, Du H, Shi FD, Cheng Y: **The Effect of Memantine on Cognitive Function and Behavioral and Psychological Symptoms in Mild-to-Moderate Alzheimer's Disease Patients.** *Dement Geriatr Cogn Disord* 2015, **40:**85-93.

299. Pomara N, Ott BR, Peskind E, Resnick EM: **Memantine treatment of cognitive symptoms in mild to moderate Alzheimer disease: secondary analyses from a placebo-controlled randomized trial.** *Alzheimer Dis Assoc Disord* 2007, **21:**60-64.

300. Orgogozo JM, Rigaud AS, Stoffler A, Mobius HJ, Forette F: **Efficacy and safety of memantine in patients with mild to moderate vascular dementia: a randomized, placebo-controlled trial (MMM 300).** *Stroke* 2002, **33:**1834-1839.

301. Ilhan Algin D, Dagli Atalay S, Ozkan S, Ozbabalik Adapinar D, Ak Sivrioz I: **Memantine improves semantic memory in patients with amnestic mild cognitive impairment: A single-photon emission computed tomography study.** *J Int Med Res* 2017, **45:**2053-2064.

302. Bakchine S, Loft H: **Memantine treatment in patients with mild to moderate Alzheimer's disease: results of a randomised, double-blind, placebo-controlled 6-month study.** *J Alzheimers Dis* 2007, **11:**471-479.

303. Roberts JS, Karlawish JH, Uhlmann WR, Petersen RC, Green RC: **Mild cognitive impairment in clinical care: a survey of American Academy of Neurology members.** *Neurology* 2010, **75:**425-431.

304. Schneider LS, Dagerman KS, Higgins JP, McShane R: **Lack of evidence for the efficacy of memantine in mild Alzheimer disease.** *Arch Neurol* 2011, **68:**991-998.

305. Pribish A, Wood N, Kalava A: **A Review of Nonanesthetic Uses of Ketamine.** *Anesthesiol Res Pract* 2020, **2020:**5798285.

306. Ballard ED, Zarate CA, Jr.: **The role of dissociation in ketamine's antidepressant effects.** *Nat Commun* 2020, **11:**6431.

307. Berman RM, Cappiello A, Anand A, Oren DA, Heninger GR, Charney DS, Krystal JH: **Antidepressant effects of ketamine in depressed patients.** *Biol Psychiatry* 2000, **47:**351-354.

308. Choudhury D, Autry AE, Tolias KF, Krishnan V: **Ketamine: Neuroprotective or Neurotoxic?** *Front Neurosci* 2021, **15:**672526.

309. Lozupone M, La Montagna M, D'Urso F, Piccininni C, Sardone R, Dibello V, Giannelli G, Solfrizzi V, Greco A, Daniele A, et al: **Pharmacotherapy for the treatment of depression in patients with alzheimer's disease: a treatment-resistant depressive disorder.** *Expert Opin Pharmacother* 2018, **19:**823-842.

310. Souza-Marques B, Santos-Lima C, Araujo-de-Freitas L, Vieira F, Jesus-Nunes AP, Quarantini LC, Sampaio AS: **Neurocognitive Effects of Ketamine and Esketamine for Treatment-Resistant Major Depressive Disorder: A Systematic Review.** *Harv Rev Psychiatry* 2021, **29:**340-350.

311. Zheng W, Zhou YL, Liu WJ, Wang CY, Zhan YN, Li HQ, Chen LJ, Li MD, Ning YP: **Neurocognitive performance and repeated-dose intravenous ketamine in major depressive disorder.** *J Affect Disord* 2019, **246:**241-247.

312. Iqbal SZ, Mathew SJ: **Ketamine for depression clinical issues.** *Adv Pharmacol* 2020, **89:**131-162.

313. Wu QJ, Tymianski M: **Targeting NMDA receptors in stroke: new hope in neuroprotection.** *Mol Brain* 2018, **11:**15.

314. Li S, Hafeez A, Noorulla F, Geng X, Shao G, Ren C, Lu G, Zhao H, Ding Y, Ji X: **Preconditioning in neuroprotection: From hypoxia to ischemia.** *Prog Neurobiol* 2017, **157:**79-91.

315. Stevens SL, Vartanian KB, Stenzel-Poore MP: **Reprogramming the response to stroke by preconditioning.** *Stroke* 2014, **45:**2527-2531.

316. Gonzales-Portillo B, Lippert T, Nguyen H, Lee JY, Borlongan CV: **Hyperbaric oxygen therapy: A new look on treating stroke and traumatic brain injury.** *Brain Circ* 2019, **5:**101-105.

317. Gu X, Jiang, M.Q., Lin, T., Shah, N., Wei, L., Yu, S.P.: **Preventive memantine treatment for the comorbidity of stroke and Alzheimer’s disease.** *Annual Meeting of Society for Neuroscience* 2022, **10922:**2022-S-10922-SfN.

318. Wang Y, Eu J, Washburn M, Gong T, Chen HS, James WL, Lipton SA, Stamler JS, Went GT, Porter S: **The pharmacology of aminoadamantane nitrates.** *Curr Alzheimer Res* 2006, **3:**201-204.

319. Chen BK, Luna VM, Shannon ME, Hunsberger HC, Mastrodonato A, Stackmann M, McGowan JC, Rubinstenn G, Denny CA: **Fluoroethylnormemantine, a Novel NMDA Receptor Antagonist, for the Prevention and Treatment of Stress-Induced Maladaptive Behavior.** *Biol Psychiatry* 2021, **90:**458-472.

320. Chen BK, Le Pen G, Eckmier A, Rubinstenn G, Jay TM, Denny CA: **Fluoroethylnormemantine, A Novel Derivative of Memantine, Facilitates Extinction Learning Without Sensorimotor Deficits.** *Int J Neuropsychopharmacol* 2021, **24:**519-531.

321. Wu L, Zhou X, Cao Y, Mak SH, Zha L, Li N, Su Z, Han Y, Wang Y, Man Hoi MP, et al: **Therapeutic efficacy of novel memantine nitrate MN-08 in animal models of Alzheimer's disease.** *Aging Cell* 2021, **20:**e13371.

322. Trudler D, Sanz-Blasco S, Eisele YS, Ghatak S, Bodhinathan K, Akhtar MW, Lynch WP, Pina-Crespo JC, Talantova M, Kelly JW, Lipton SA: **alpha-Synuclein Oligomers Induce Glutamate Release from Astrocytes and Excessive Extrasynaptic NMDAR Activity in Neurons, Thus Contributing to Synapse Loss.** *J Neurosci* 2021, **41:**2264-2273.

323. Ghatak S, Dolatabadi N, Gao R, Wu Y, Scott H, Trudler D, Sultan A, Ambasudhan R, Nakamura T, Masliah E, et al: **NitroSynapsin ameliorates hypersynchronous neural network activity in Alzheimer hiPSC models.** *Mol Psychiatry* 2021, **26:**5751-5765.

324. Savchenko A, Braun GB, Molokanova E: **Nanostructured Antagonist of Extrasynaptic NMDA Receptors.** *Nano Lett* 2016, **16:**5495-5502.

325. Zoladz PR, Campbell AM, Park CR, Schaefer D, Danysz W, Diamond DM: **Enhancement of long-term spatial memory in adult rats by the noncompetitive NMDA receptor antagonists, memantine and neramexane.** *Pharmacol Biochem Behav* 2006, **85:**298-306.

326. Rammes G: **Neramexane: a moderate-affinity NMDA receptor channel blocker: new prospects and indications.** *Expert Rev Clin Pharmacol* 2009, **2:**231-238.

327. Suckfull M, Althaus M, Ellers-Lenz B, Gebauer A, Gortelmeyer R, Jastreboff PJ, Moebius HJ, Rosenberg T, Russ H, Wirth Y, Krueger H: **A randomized, double-blind, placebo-controlled clinical trial to evaluate the efficacy and safety of neramexane in patients with moderate to severe subjective tinnitus.** *BMC Ear Nose Throat Disord* 2011, **11:**1.

328. Zhang J, Wang C, Deng T, Xue Z, Chen X, Chang L, Wang Q: **The preventive effect of NR2B and NR2D-containing NMDAR antagonists on Abeta-induced LTP disruption in the dentate gyrus of rats.** *Metab Brain Dis* 2013, **28:**697-704.

329. Acker TM, Yuan H, Hansen KB, Vance KM, Ogden KK, Jensen HS, Burger PB, Mullasseril P, Snyder JP, Liotta DC, Traynelis SF: **Mechanism for noncompetitive inhibition by novel GluN2C/D N-methyl-D-aspartate receptor subunit-selective modulators.** *Mol Pharmacol* 2011, **80:**782-795.

330. Hamstra SI, Roy BD, Tiidus P, MacNeil AJ, Klentrou P, MacPherson REK, Fajardo VA: **Beyond its psychiatric use: the benefits of low dose lithium supplementation.** *Curr Neuropharmacol,* 2022, **Online ahead of print:**doi: 10.2174/1570159X20666220302151224.

331. Haussmann R, Noppes F, Brandt MD, Bauer M, Donix M: **Lithium: A therapeutic option in Alzheimer's disease and its prodromal stages?** *Neurosci Lett* 2021, **760:**136044.

332. Basselin M, Chang L, Bell JM, Rapoport SI: **Chronic lithium chloride administration attenuates brain NMDA receptor-initiated signaling via arachidonic acid in unanesthetized rats.** *Neuropsychopharmacology* 2006, **31:**1659-1674.

333. Monaco SA, Ferguson BR, Gao WJ: **Lithium Inhibits GSK3beta and Augments GluN2A Receptor Expression in the Prefrontal Cortex.** *Front Cell Neurosci* 2018, **12:**16.

334. Mohammad Jafari R, Ghahremani MH, Rahimi N, Shadboorestan A, Rashidian A, Esmaeili J, Ejtemaei Mehr S, Dehpour AR: **The anticonvulsant activity and cerebral protection of chronic lithium chloride via NMDA receptor/nitric oxide and phospho-ERK.** *Brain Res Bull* 2018, **137:**1-9.

335. Bosche B, Schafer M, Graf R, Hartel FV, Schafer U, Noll T: **Lithium prevents early cytosolic calcium increase and secondary injurious calcium overload in glycolytically inhibited endothelial cells.** *Biochem Biophys Res Commun* 2013, **434:**268-272.

336. Shim S.S. LAM, Kapecki N., Briggs C.A., Stutzmann G.E: **Lithium suppresses calcium signaling, nitrosative stress and tauopathy and enhances synaptic plasticity in 3xTg-AD mice.** *Alzheimer’s Association International Conference* 2019, **Abstract:**32321.

337. Shim SS, Stutzmann GE: **Inhibition of Glycogen Synthase Kinase-3: An Emerging Target in the Treatment of Traumatic Brain Injury.** *J Neurotrauma* 2016, **33:**2065-2076.

338. Hoque A, Hossain MI, Ameen SS, Ang CS, Williamson N, Ng DC, Chueh AC, Roulston C, Cheng HC: **A beacon of hope in stroke therapy-Blockade of pathologically activated cellular events in excitotoxic neuronal death as potential neuroprotective strategies.** *Pharmacol Ther* 2016, **160:**159-179.

339. Lee JH, Zhang JY, Wei ZZ, Yu SP: **Impaired social behaviors and minimized oxytocin signaling of the adult mice deficient in the N-methyl-d-aspartate receptor GluN3A subunit.** *Exp Neurol* 2018, **305:**1-12.

340. Yashiro K, Philpot BD: **Regulation of NMDA receptor subunit expression and its implications for LTD, LTP, and metaplasticity.** *Neuropharmacology* 2008, **55:**1081-1094.

341. Sanz-Clemente A, Nicoll RA, Roche KW: **Diversity in NMDA receptor composition: many regulators, many consequences.** *Neuroscientist* 2013, **19:**62-75.

342. Arrazola MS, Varela-Nallar L, Colombres M, Toledo EM, Cruzat F, Pavez L, Assar R, Aravena A, Gonzalez M, Montecino M, et al: **Calcium/calmodulin-dependent protein kinase type IV is a target gene of the Wnt/beta-catenin signaling pathway.** *J Cell Physiol* 2009, **221:**658-667.

343. Hardingham GE, Arnold FJ, Bading H: **A calcium microdomain near NMDA receptors: on switch for ERK-dependent synapse-to-nucleus communication.** *Nat Neurosci* 2001, **4:**565-566.

344. Wu GY, Deisseroth K, Tsien RW: **Activity-dependent CREB phosphorylation: convergence of a fast, sensitive calmodulin kinase pathway and a slow, less sensitive mitogen-activated protein kinase pathway.** *Proc Natl Acad Sci U S A* 2001, **98:**2808-2813.

345. Screaton RA, Conkright MD, Katoh Y, Best JL, Canettieri G, Jeffries S, Guzman E, Niessen S, Yates JR, 3rd, Takemori H, et al: **The CREB coactivator TORC2 functions as a calcium- and cAMP-sensitive coincidence detector.** *Cell* 2004, **119:**61-74.

346. Lau D, Bading H: **Synaptic activity-mediated suppression of p53 and induction of nuclear calcium-regulated neuroprotective genes promote survival through inhibition of mitochondrial permeability transition.** *J Neurosci* 2009, **29:**4420-4429.

347. Leveille F, Papadia S, Fricker M, Bell KF, Soriano FX, Martel MA, Puddifoot C, Habel M, Wyllie DJ, Ikonomidou C, et al: **Suppression of the intrinsic apoptosis pathway by synaptic activity.** *J Neurosci* 2010, **30:**2623-2635.

348. Chrivia JC, Kwok RP, Lamb N, Hagiwara M, Montminy MR, Goodman RH: **Phosphorylated CREB binds specifically to the nuclear protein CBP.** *Nature* 1993, **365:**855-859.

349. Al-Mubarak B, Soriano FX, Hardingham GE: **Synaptic NMDAR activity suppresses FOXO1 expression via a cis-acting FOXO binding site: FOXO1 is a FOXO target gene.** *Channels (Austin)* 2009, **3:**233-238.

350. Nucifora FC, Jr., Sasaki M, Peters MF, Huang H, Cooper JK, Yamada M, Takahashi H, Tsuji S, Troncoso J, Dawson VL, et al: **Interference by huntingtin and atrophin-1 with cbp-mediated transcription leading to cellular toxicity.** *Science* 2001, **291:**2423-2428.

351. Lu HT, Feng RQ, Tang JK, Zhou JJ, Gao F, Ren J: **CaMKII/calpain interaction mediates ischemia/reperfusion injury in isolated rat hearts.** *Cell Death Dis* 2020, **11:**388.

352. Van Laar VS, Roy N, Liu A, Rajprohat S, Arnold B, Dukes AA, Holbein CD, Berman SB: **Glutamate excitotoxicity in neurons triggers mitochondrial and endoplasmic reticulum accumulation of Parkin, and, in the presence of N-acetyl cysteine, mitophagy.** *Neurobiol Dis* 2015, **74:**180-193.

353. Uddin MS, Stachowiak A, Mamun AA, Tzvetkov NT, Takeda S, Atanasov AG, Bergantin LB, Abdel-Daim MM, Stankiewicz AM: **Autophagy and Alzheimer's Disease: From Molecular Mechanisms to Therapeutic Implications.** *Front Aging Neurosci* 2018, **10:**04.

354. Nguyen D, Alavi MV, Kim KY, Kang T, Scott RT, Noh YH, Lindsey JD, Wissinger B, Ellisman MH, Weinreb RN, et al: **A new vicious cycle involving glutamate excitotoxicity, oxidative stress and mitochondrial dynamics.** *Cell Death Dis* 2011, **2:**e240.

355. Xia Y, Wang ZH, Zhang Z, Liu X, Yu SP, Wang JZ, Wang XC, Ye K: **Delta- and beta- secretases crosstalk amplifies the amyloidogenic pathway in Alzheimer's disease.** *Prog Neurobiol* 2021, **204:**102113.

356. Jack CR, Jr., Knopman DS, Jagust WJ, Petersen RC, Weiner MW, Aisen PS, Shaw LM, Vemuri P, Wiste HJ, Weigand SD, et al: **Tracking pathophysiological processes in Alzheimer's disease: an updated hypothetical model of dynamic biomarkers.** *Lancet Neurol* 2013, **12:**207-216.

357. Reisberg B, Doody R, Stoffler A, Schmitt F, Ferris S, Mobius HJ, Memantine Study G: **Memantine in moderate-to-severe Alzheimer's disease.** *N Engl J Med* 2003, **348:**1333-1341.

358. Doody R, Wirth Y, Schmitt F, Mobius HJ: **Specific functional effects of memantine treatment in patients with moderate to severe Alzheimer's disease.** *Dement Geriatr Cogn Disord* 2004, **18:**227-232.

359. Profyri E, Leung P, Huntley J, Orgeta V: **Effectiveness of treatments for people living with severe dementia: A systematic review and meta-analysis of randomised controlled clinical trials.** *Ageing Res Rev* 2022, **82:**101758.

360. Hellweg R, Wirth Y, Janetzky W, Hartmann S: **Efficacy of memantine in delaying clinical worsening in Alzheimer's disease (AD): responder analyses of nine clinical trials with patients with moderate to severe AD.** *Int J Geriatr Psychiatry* 2012, **27:**651-656.

361. Luo F, Wu L, Zhang Z, Zhu Z, Liu Z, Guo B, Li N, Ju J, Zhou Q, Li S, et al: **The dual-functional memantine nitrate MN-08 alleviates cerebral vasospasm and brain injury in experimental subarachnoid haemorrhage models.** *Br J Pharmacol* 2019, **176:**3318-3335.

362. Duthie A, van Aalten L, MacDonald C, McNeilly A, Gallagher J, Geddes J, Lovestone S, Sutherland C: **Recruitment, Retainment, and Biomarkers of Response; A Pilot Trial of Lithium in Humans With Mild Cognitive Impairment.** *Front Mol Neurosci* 2019, **12:**163.

363. Aprahamian I, Santos FS, dos Santos B, Talib L, Diniz BS, Radanovic M, Gattaz WF, Forlenza OV: **Long-term, low-dose lithium treatment does not impair renal function in the elderly: a 2-year randomized, placebo-controlled trial followed by single-blind extension.** *J Clin Psychiatry* 2014, **75:**e672-678.

364. Forlenza OV, Diniz BS, Radanovic M, Santos FS, Talib LL, Gattaz WF: **Disease-modifying properties of long-term lithium treatment for amnestic mild cognitive impairment: randomised controlled trial.** *Br J Psychiatry* 2011, **198:**351-356.

365. Berthier ML, Green C, Lara JP, Higueras C, Barbancho MA, Davila G, Pulvermuller F: **Memantine and constraint-induced aphasia therapy in chronic poststroke aphasia.** *Ann Neurol* 2009, **65:**577-585.

366. Kafi H, Salamzadeh J, Beladimoghadam N, Sistanizad M, Kouchek M: **Study of the neuroprotective effects of memantine in patients with mild to moderate ischemic stroke.** *Iran J Pharm Res* 2014, **13:**591-598.

367. Babak Bakhshayesh-Eghbali MH, Seyed-Mohammad Seyed-Saadat, Seyed-Nazanin Seyed-Saadat, Ehsan Kazemnezhad-Leili, Melina Rouhi-Rad: **Comparing the Effect of Memantine and Placebo on Clinical Outcome of Intracranial Hemorrhage: A Randomized Double Blind Clinical Trial.** *Caspian J Neurological Sciences* 2015, **1:**11-18.

368. Beladi Moghadam N, Pourheidar E, Ahmadpour F, Kafi H, Salamzadeh J, Nasiri S, Sistanizad M: **The effects of memantine on the serum concentrations of matrix metalloproteinases and neurologic function of patients with ischemic stroke.** *J Clin Neurosci* 2021, **90:**268-272.

369. Hong JM, Choi MH, Sohn SI, Hwang YH, Ahn SH, Lee YB, Shin DI, Chamorro A, Choi DW, on the behalf of the Si: **Safety and Optimal Neuroprotection of neu2000 in acute Ischemic stroke with reCanalization: study protocol for a randomized, double-blinded, placebo-controlled, phase-II trial.** *Trials* 2018, **19:**375.

370. Lan CC, Liu CC, Lin CH, Lan TY, McInnis MG, Chan CH, Lan TH: **A reduced risk of stroke with lithium exposure in bipolar disorder: a population-based retrospective cohort study.** *Bipolar Disord* 2015, **17:**705-714.

371. Pichardo-Rojas D, Pichardo-Rojas PS, Cornejo-Bravo JM, Serrano-Medina A: **Memantine as a neuroprotective agent in ischemic stroke: Preclinical and clinical analysis.** *Front Neurosci* 2023, **17:**1096372.
