# Supplementary material for: Extrasynaptic NMDA receptors in acute and chronic excitotoxicity: implications for preventive treatments of ischemic stroke and late-onset Alzheimer’s disease
Source: Mol Neurodegener. 2023 Jul 3;18:43. doi: 10.1186/s13024-023-00636-1 (PMC10318843; doi:10.1186/s13024-023-00636-1)
Supplement: Supplementary file 2 — Supplementary Material 2 [file 13024_2023_636_MOESM2_ESM.docx]

May 15, 2023

Dr. Lucy Job
Molecular Neurodegeneration

Dear Editor Lucy Job:

I would like to thank you and Reviewers for the effort in reviewing our submitted paper: “Extrasynaptic NMDA Receptors in Acute and Chronic Excitotoxicity: Implications for Preventive Treatments Against Comorbidity of Ischemic Stroke and Late-onset Alzheimer’s Disease”. In the second review, Reviewer 2 expressed full satisfaction with the revised manuscript. Reviewer 1 provides additional comments and suggestions, which are helpful for further improvements of the paper. We have made all revisions following proposals from Reviewer 2.

In the decision letter, it was mentioned that the revision should also include rewriting the Abstract and adding references suggested by a third Reviewer. Unfortunately, we could not identify this Reviewer’s comments in the letter nor on the journal website. In any case, we have revised the Abstract according to the Journal guideline (350 word limit) and added additional references related to the regulatory role of GluN3 in NMDAR activity. For “not validated” papers in the reference list specified by the editing Editor, we now provide the PMID numbers to facilitate validations of these papers.

A clean final manuscript, a copy of this manuscript showing all tracked changes, and a point-by-point response to the Reviewers’ comments are included in the resubmission. The revised Figure 3 replaced the previous figure.

Thank you for your consideration. I hope these revisions have significantly improved the quality and accuracy of this timely summary needed for both basic and clinical neuroscience research.

Sincerely,

Shan Ping Yu

Emory University School of Medicine
